# Health problems in children with profound intellectual and multiple disabilities: a scoping review

**DOI:** 10.1007/s00431-024-05876-x

**Published:** 2024-12-06

**Authors:** Lian M. Zandbelt, Esther J. Bakker-van Gijssel, Catelijne H. Coppens, Jos M. T. Draaisma, Joyce M. Geelen

**Affiliations:** 1 Department of Paediatrics, Radboud Institute for Health Sciences, Radboud University Medical Centre, Amalia Children’s Hospital, Nijmegen, The Netherlands; 2 Department of Primary and Community Care, Radboud University Medical Centre, Nijmegen, The Netherlands; 3 Siza, Residential Care Facility for People With Disabilities, Arnhem, The Netherlands

**Keywords:** Children, Clinical conditions, Health problems, Profound intellectual and multiple disabilities, Scoping review

## Abstract

This study aims to investigate and categorize health problems in children with profound intellectual and multiple disabilities (PIMD). We conducted a scoping review in five electronic databases (PubMed, Embase, Medline, PsychInfo, and Web of Science), including peer-reviewed studies that describe health problems in children with PIMD. Articles on PIMD were included when mentioning children with severe or profound intellectual disability (IQ/DQ < 35 or developmental age < 24 months) AND severe/profound motor impairment (GMFCS IV/V, non-ambulatory/not walking without support or gross motor function measure (GMFM-66) score < 40). Health problems were divided into 11 different categories based on literature and the experiences of the authors. Both health problems reported by caregivers and diagnosed by health professionals were included. A data extraction tool was developed using the JBI checklist recommendations. The review included 22 studies, with a total of 894 patients across all the articles. The most reported health problems in children with PIMD were epilepsy, respiratory infections, feeding difficulties, GERD, scoliosis, and visual impairment. There was no uniformity in the definition and terminology of PIMD. *Conclusion*: The studies in this review covered a wide range of health problems, with only a few focusing on the entire range. Given the lack of knowledge about children with PIMD, further research is required on the presence of health problems, the age of onset, and appropriate treatments. Awareness, early detection, and treatment of the health problems could optimize the medical care and quality of life of children with PIMD.

**Table:** 

**What is Known:** *• Children with profound intellectual and multiple disabilities (PIMD) have severe intellectual and motor disabilities and various comorbidities.* *• Children often visit the hospital and provided care is complex and fragmented.*
**What is New:** *• Children with PIMD develop a wide range of health problems; most reported are epilepsy, respiratory infections, and feeding difficulties.* *• Little is known about treatment of health problems in children with PIMD.*

**Supplementary Information:**

The online version contains supplementary material available at 10.1007/s00431-024-05876-x.

## Introduction

Children with profound intellectual and multiple disabilities (PIMD) are severely limited in all aspects of their lives. According to various sources, the definition of PIMD can be described as a combination of a motor disability classified as gross motor function classification system (GMFCS) level IV or V and an intellectual disability classified as intellectual quotient (IQ) < 25–35 or developmental age of up to 24–36 months [1–4]. Also, this group experiences sensory problems, and they have intensive support needs. However, there is no uniformity in definition [5]. The etiological causes of the disabilities can be divided into congenital (genetic, metabolic, and neurologic) and acquired (complications during pregnancy and delivery, diseases, or toxic exposure) [3]. It is known that the PIMD group experiences many health problems, like constipation, visual impairment, epilepsy, spasticity, deformations, incontinence, and reflux [6]. However, limited research has been performed on children with PIMD; their characteristics and health needs have not been extensively studied so far. Children with PIMD often have multiple medical problems and generally visit the hospital more often than their peers without PIMD [7]. Due to the variety and complexity of medical conditions and health problems, many different medical specialists (e.g. pediatricians, rehabilitation physicians) and other medical professionals (e.g. physiotherapists, speech therapists, and occupational therapists) are involved. As such, the care for children with PIMD is fragmented; it is not always clear who coordinates the care of these children [8].

Health problems have a burden on both children and their care system [9]. Families with a child with PIMD struggle with the strain of frequent hospital appointments. Seliner et al. showed that in addition to the emotional strain, there are also financial consequences for families with a child with PIMD [10]. For example, parents may have to take time off work, arrange transportation, and provide care for other children. This puts a heavy burden on both the child and their care system [9].

Due to the complexity of their needs, children with PIMD require a comprehensive approach to their care. Early detection of health problems (medical, psychological, and behavioural) is essential to improve or sustain their health and quality of life [11]. Proactive care is needed to prevent unscheduled outpatient care appointments and hospitalizations [12]. Undiagnosed and untreated health problems can lead to developing secondary health problems [13]. Therefore, active screening for health problems at the relevant ages is important.

In this scoping review, we investigate and categorize which health problems are reported in children with PIMD. Therefore, we address the following main question: What are the health problems that occur in children with PIMD? We also address the sub-questions: Can we designate age periods in which the various health problems occur? What is known about the treatment of health problems in children with PIMD?

## Methods

This scoping review was conducted in accordance with the standards of the Preferred Reporting Items for Systematic Reviews and Meta-Analyses extension for Scoping Reviews (PRISMA-ScR, Online Resource 1) [14], according to the protocol available at https://osf.io/vzumj. The study methodology draws on the framework proposed by Arksey and O’Malley, which was further developed by Levac, Colquhoun, and O’Brien [15, 16]. This approach involves five key stages: identifying the research question; identifying relevant studies; selecting the studies; extracting the data; and collecting, summarizing, and reporting the results. The protocol for the study can be found in Online Resource 2. In this scoping review, we aimed to provide an overview of the available literature on health problems, rather than attempting to create empirical evidence on this topic.

### Search strategy

We conducted searches in PubMed, Embase, Medline, PsychInfo, and Web of Science. The search strategy was based on the patient group of children with PIMD and health problems. The intellectual disability part was adjusted by Wissing et al. [17]. The full search strategy can be found in Online Resource 3. The last search was conducted on July 24, 2024. The inclusion criteria are shown in Box 1. We set IQ/DQ at < 35 and developmental age at < 24 months, since we expected the level of functioning to still be relatively low; we wanted to make the search as broad as possible. We used studies published in English or Dutch, with no limitations regarding publication year. In order to gain substantive knowledge on this specific patient group while ensuring the quality of included studies, only peer-reviewed articles were included. Studies of various designs were included: case–control studies, cohort studies, randomized controlled trials, and case reports or case series, as well as studies with clinical data as parents’ questionnaires were included. Systematic reviews, meta-analyses, and letters were excluded. We scanned the reference lists of included studies to supplement the search, using the same methods and criteria.

### Selection of studies

The search results were collected and deduplicated in Endnote and then exported into the Rayyan software for ease of management [18]. One reviewer (CC, JD, JG, or LZ) screened the abstracts for eligibility, consulting a second reviewer (JG) when necessary. Two reviewers (LZ and JG) screened the first 200 abstracts together to gain a sense of the process. Two reviewers (CC, EB, JG, and LZ) independently assessed the full text of selected records in detail against the inclusion criteria. Any disagreement between the reviewers was resolved through discussion or by consulting a third independent reviewer (JD).

**Box 1** Inclusion criteria for full text screening.

**Table:** 

Inclusion criteria - PIMD consists of: o Severe or profound intellectual disability/mental retardation (IQ/DQ < 35 or developmental age < 24 months) AND o Severe/profound motor impairment; cerebral palsy GMFCS IV or V; non-ambulatory/not walking without support; or gross motor function measure (GMFM-66) score < 40 - > 75% of the study population was diagnosed with PIMD - > 90% of the study population were children aged ≤ 18 years - Description of (the prevalence of) health problems	

### Data extraction

One reviewer (LZ) extracted the data from the papers included in this scoping review with the supervision of JG to ensure accuracy. The authors (LZ, JG, EB) developed a data extraction tool to consider the JBI checklist recommendations [19]. Data extraction included specific details about the participant characteristics, the health problems divided into different categories, the age of health problem onset, and the treatment. The following items were also noted for each selected article: title, first author, DOI, year of publication, region, study type, age, and critical appraisal (inclusion and exclusion criteria of the included article).

### Data analysis and presentation

The health problems were initially divided into nine different categories (cardiac, respiratory, oropharyngeal, infectious, gastro-intestinal, neurologic, musculoskeletal, psychological (behavioural), and skin and sensory disorders, see also the study protocol) based on adult literature [6] and personal experiences; however, during the study process, this was expanded to 11 different categories. Within the categories, the prevalence of each health problem per total patient with a health problem in the included studies was calculated. Therefore, the number of patients per health problem can be different.

## Results

### Study selection process

**Fig. 1:**
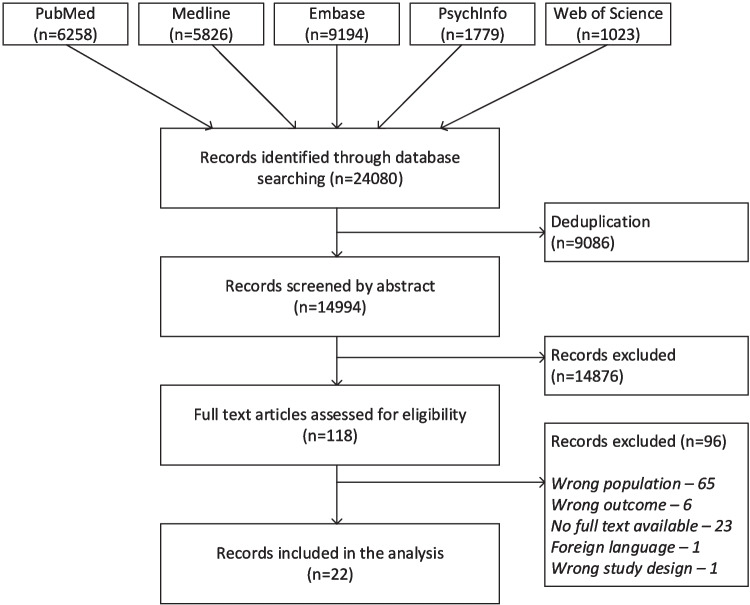
Flowchart of study selection process

The search identified 24,080 records (see Fig. 1). After removing duplicates (*n* = 9,086), an initial screening of abstracts of the 14,994 remaining records was performed. A second reviewer was consulted for approximately 200 cases. This screening excluded 14,876 records. The full texts of the remaining 118 records were assessed by two independent reviewers. A third reviewer was consulted for 15 of these articles to resolve any discrepancies or uncertainties. Based on the inclusion criteria shown in Box 1, 96 records were excluded. Finally, 22 publications were included and used for the review. Checking the references did not result in the inclusion of extra studies. The study selection process and exclusion criteria are shown in Fig. 1.

### Description of included studies

Table 1 shows the general characteristics of the studies. The number of participants ranged from 1 to 217, with a total of 894 children in all the studies together. The mean age of the children in 20 out of 22 of the studies was 7 years and 2 months (SD, 2 years and 10 months). In two studies with no available mean age, the medians were 21 months and 12 years [20, 21]. Both males and females were included in 18 out of 22 of the studies. In the study of O’Brien et al. [22], two sisters were included with the same frameshift mutation. A total of 519 males and 375 females were included. The publication years of the included studies ranged from 2005 to 2024.

**Table 1:** Characteristics of studies included in this scoping review

References	Region	Study type	Study aim	Study population	Definition/classification
Al Mutairi et al. [23]	Asia	Retrospective chart review	Review the clinical and molecular diagnostic findings of 23 Arab children with Aicardi-Goutières syndrome (AGS) in an Arab population	24 children with Aicardi-Goutières syndrome	Profound intellectual disability Spasticity and truncal hypotonia
Borgione et al. [24]	Europe	Case report	Report a novel m.7484A > G homoplasmic mutation in the tRNASer(UCN) gene affecting the third base of the anticodon triplet in a girl and her mother presenting with mitochondrial encephalomyopathy and sensorineural hearing loss	1 child with mitochondrial encephalomyopathy	Profound intellectual disability Spastic tetraplegia
Campanozzi et al. [25]	Europe	Prospective clinical data	Assess the possible relationship between malnutrition and gastrointestinal problems and to evaluate the role of nutrition on their gross motor abilities	21 children with cerebral palsy and severe mental retardation	Severe mental retardation Cerebral palsy (GMFM of 4.13 and 13.7)
Fattal-Valevski et al. [26]	Asia	Retrospective chart review	To delineate the phenotype and developmental outcomes in patients diagnosed with this mutation	15 children with *MED17*-related disease in Caucasus-Jewish families	Profound intellectual disability Progressive spasticity and spastic quadriplegia (wheelchair bound)
Holenweg-Gross et al. [27]	Europe	Prospective clinical data	Determine the prevalence of undernutrition and explore its influence on QOL	72 children with PIMD	Severe to profound mental retardation (DQ < 35) GMFCS IV or V
Kubota et al. [28]	North America	Case report	Report the spontaneous remission of epileptic seizures following norovirus-induced viral gastroenteritis in a patient carrying a DNM1 variant	1 child with *DNM1* encephalopathy	Profound psychomotor developmental delay Unable to walk
Lancioni et al. [29]	Europe	Prospective clinical data	Supporting the already available evidence about effectiveness of the approach in motivating and promoting children’s ambulation	5 children with severe or profound intellectual and motor disabilities (SPIMD)	Severe or profound intellectual disability Walk a few steps with extensive physical support
Li et al. [30]	Asia	Case report	Identify a novel splicing variant, c.935-6C > G, of PIGS, and report a ventricular septal defect in patients with PIGS variants and review the clinical and genetic features of PIGS	1 child with a *PIGS* variant	Profound intellectual disability Unable to sit
Mensch et al. [31]	Europe	Prospective clinical data	Investigate the construct validity of Movakic and responsiveness to change	60 children with severe multiple disabilities (SMD)	Profound intellectual disability (IQ < 25) GMFCS IV or V
Mol-Bakker et al. [32]	Europe	Prospective clinical data	To gain insight into the prevalence of physical health conditions and associations between these conditions in young children with PIMD	51 young children with PIMD	Significant cognitive developmental delay less than a quarter of their chronological age GMFCS level IV or V
Nakamoto et al. [33]	Asia	Retrospective chart review	To investigate whether corticosteroid therapy was associated with better clinical outcomes for SMID children with LRTIs	217 children with severe motor and intellectual disabilities (SMID)	IQ or DQ < 20 Bedridden or unable to sit, crawl, or walk with support
O’Brien et al. [22]	North America	Case reports	Describing the clinical features, serial neuroimaging and long-term follow-up of two sisters with a severe neurodegenerative phenotype who are homozygous for a novel frameshift variant in *CSTB*	2 children with a homozygous frameshift mutation in *CSTB*	Profound global developmental delay Unable to independently sit and non-ambulatory
Onoyama et al. [20]	Asia	Retrospective case–control study	Investigate the severity of respiratory synctial virus-LRTI in children with SMID	18 children with severe motor intellectual disability (SMID) (43 healthy controls)	IQ or DQ < 20 Bedridden or unable to sit, crawl, or walk with support
Proesmans et al. [21]	Europe	Prospective clinical data	Assess the respiratory morbidity in a broad group of children with profound intellectual and multiple disabilities (PIMD) AND assess possible risk factors including skeletal, neuro-motor- and feeding-related parameters	127 children with PIMD	Profound cognitive disability (IQ < 20–25 or developmental age < 2 years) Severe sensory and/or motor impairments
Ramirez et al. [34]	North America	Retrospective chart review	The purpose of this study is to present the natural history and musculoskeletal manifestations of a neonatal nonketotic hyperglycinaemia (NKH) patient group	12 children with neonatal nonketotic hyperglycinaemia	Severe global mental retardation Non-ambulatory
Sabanathan et al. [35]	Multi-regional	Retrospective chart review	Describing clinico-radiological and diagnostic details and thereby supporting the clinician faced with a hypotonic infant, to develop a phenotypic approach to *TBCK*-related disorders	6 children with biallelic *TBCK* (TBC1 domain-containing kinase) pathogenic variants	Significant cognitive impairment Not able to sit, (severe) generalized hypotonia
Sadleir et al. [36]	Oceania	Case reports	To define a distinct *SCN1A* developmental and epileptic encephalopathy with early onset, profound impairment, and movement disorder	9 children with profound developmental and epileptic encephalopathy and *SCN1A* mutation	Profound intellectual disability Non-ambulatory
Tadema et al. [37]	Europe	Parents questionnaire	The type of basic needs caring tasks of parents in the NL on a broad range of topics related to health and basic needs and considering the perceptions of parents concerning the care load	Parents of 133 children with PIMD	Profound intellectual disability (IQ < 20) Motor disabilities (the children are non-ambulant and cannot use their hands and arms or only to a limited extent)
Ten Brug et al. [38]	Europe	Prospective clinical study	Describe the knowledge teachers have available about the contextual, motor and, sensory abilities and preferences of a child with PIMDs and to specifically focus on changes when applying this knowledge during storytelling with multi-sensory storytelling	3 children with PIMD	IQ < 25 Severe or profound motor disabilities
Tsuji et al. [39]	Asia	Case reports	Report two cases with GNB1 variants who developed acute encephalopathies at different ages with severe neurological sequelae	2 children with *GNB1* variants	GMFCS 5 (severe spastic tetraplegia) IQ < 20 (severe intellectual disabilities)
Wakamoto et al. [40]	Asia	Prospective clinical study	Serum KL-6 levels in children with SMIDS undergoing video fluorography evaluation were examined to ascertain whether or not KL-6 is a clinically useful indicator for detecting the presence of chronic aspiration	66 children with severe motor and intellectual disabilities (SMID)	IQ < 35 GMFCS IV or V
Zijlstra et al. [41]	Europe	Retrospective chart review	To collect data on the type and frequency of the medical conditions the children with profound intellectual and multiple disabilities encounter and the impact of these problems on the performance of planned therapies and activities	48 children with PIMD	Profound intellectual disability Profound motor disability

*DQ* developmental quotient, *GMFCS* gross motor function classification system,* GMFM* gross motor function measure, *IQ* intellectual quotient, *LRTI* lower respiratory tract infection, *PIMD* profound intellectual and multiple disabilities

### Reported health problems in children with PIMD

Table 2 shows the described health problems divided into the different clinical conditions. Both neurologic and gastro-intestinal conditions together were noted in 15 of the 22 studies. Seven studies found both oropharyngeal and respiratory conditions together.

**Table 2:** Reported health problems in children with PIMD described in the included studies divided into clinical conditions

	*N* = 22 studies	*N* = 894 patients
**Neurologic** Epilepsy [21–24, 26–32, 34–41] Hypotonia [21–24, 28, 30, 32, 34–36, 39] Spasticity [22, 23, 26, 28, 29, 32, 34, 38] Divergent MRI findings [22–24, 30, 36, 39] Encephalopathy [22, 23, 29, 39]	19	479/638 171/236 82/113 32/39 22/33
**Respiratory** Respiratory infection [20, 26, 32, 33, 35] Unspecified respiratory problems [24, 31, 32, 34, 37, 39, 41] Lobar of segmental consolidation [33] Aspiration [26, 40] Asthma [32, 33] Pneumonia [20, 40] Pneumothorax [39]	13	238/307 130/287 115/127 107/208 45/268 27/84 1/2
**Sensory disorders** Visual impairment [22–24, 26, 28–32, 35, 38, 41] Other sensory disability (tactile) [41] Hearing impairment [24, 29, 32, 35, 38, 41]	12	142/217 26/48 20/114
**Psychological/behavioural** Self-injurious behaviour [37] Behaviour problems [41] Crying [37] Poor feeding in relation to irritability [22, 23, 26] Sleeping difficulties [41]	5	40/133 24/48 23/133 22/41 15/48
**Skin disorders** Skin problems [37]	1	35/133
**Gastro-intestinal** GERD[21, 25, 27, 32, 34, 37, 40] Feeding difficulties^a^[21, 22, 26, 27, 31, 33–35, 38, 39, 41] Obstipation/constipation [25, 32, 37, 38] GI problems[25, 28, 41] Undernutrition [25, 27] Obesity[27] Abdominal pain[22]	17	183/482 177/564 90/208 52/120 39/93 7/72 1/2
**Musculoskeletal** Scoliosis[21, 24, 27, 31, 32, 34–36] Skeletal deformation[24, 32, 37, 39] Contractures[22, 31, 32, 34] Kyphoscoliosis[36] Osteoporotic/pathologic fractures[24, 27, 34, 35] Hip dislocation[24, 34]	11	113/216 51/136 31/75 1/9 9/91 6/13
**Oropharyngeal** Dysphagia[21] Tracheostomy[20, 33, 35] Dental problems[37, 41] Drinking problems[38]	8	69/127 50/243 19/181 1/3
**Infectious/haematologic/ endocrinologic** Hepato(spleno)megaly[23, 30] Thrombocytopenia[23] Hypothyroidism[35] Primary or secondary immunodeficiency	3	6/25 4/24 1/6 0/18
**Cardiac** Congenital heart disease[20, 30, 32]	3	9/70
**Urogenital** Undescended testes[35, 39] Bilateral hydronephrosis[39]	2	3/8 1/2

*GERD* gastroesophageal reflux disease, *GI* gastro-intestinal

^a^Feeding difficulties also consist of gastrostomy, regurgitating, PEG tube, nasogastric fed, and gastro-jejunal tube

In terms of neurologic conditions, epilepsy was the most reported problem, diagnosed in 479 of 638 children, followed by hypotonia in 171 of 236 children. Within the gastro-intestinal condition category, gastroesophageal reflux disease (GERD) was identified in seven studies and 183 of 482 children. Feeding difficulties were found in 177 of 564 children. Respiratory infections were mentioned in five studies, with a total of 238 of 307 children, and respiratory infections due to aspiration were mentioned in three studies. Eight studies mentioned scoliosis. Twelve of the studies indicated visual impairment. Primary or secondary immunodeficiency was not reported in any patient. Skin problems were reported in 35 of 133 children in one study. Hypothyroidism, pneumothorax, bilateral hydronephrosis, and undescended testes were reported as being less prevalent.

### Definition of PIMD

The used definition of PIMD in the international literature was heterogenous. Five studies used a description other than PIMD: severe or profound intellectual and motor disabilities (SPIMD), severe multiple disabilities (SMD), and severe motor and intellectual disabilities (SMID). In six studies, the patient group was referred to as PIMD. The definition was variably interpretated, with IQ varying between < 20 and < 35. The used terminology for cognitive functioning was severe/profound intellectual disability, (global) mental retardation, cognitive disability, and developmental delay. Five of the included studies mentioned the GMFCS score. Other terms to classify motor function were cerebral palsy; GMFM; ability to sit, walk, or crawl; non-ambulatory; severe/profound motor impairment; and severe/profound motor disabilities.

### Age periods and treatment

No included studies indicated specific age stages for the development of health problems. Ten studies addressed some information for the research question regarding effective treatment. The studies mentioned the efficacy of the treatment of epilepsy in their patient [24, 26, 28, 30, 36, 39], treatment of fractures [20, 34], and respiratory treatment including ventilation, systemic corticosteroids, and home oxygen therapy [33, 35].

## Discussion

In this scoping review, 22 included studies were about health problems in children with PIMD. The most frequently reported health problems in children with PIMD were epilepsy, respiratory infections, feeding difficulties, GERD, scoliosis, and visual impairment. In the literature, no widely accepted definition and terminology were used for this specific patient group. This study found no substantial information about the age of health problem onset nor about specific treatment for health problems in children.

### Comparison with the literature

Despite our extensive search, only 22 articles were included. This highlights the need for more systematic research on health problems in children with PIMD. Children with PIMD are an understudied population with a need for complex and multidisciplinary medical care [5].

Some studies included both children and adults; however, these studies were not eligible because of our “age” exclusion criteria [3, 42–45]. In the study of Rousseau et al. [44], the research group consisted of both children and adults (*n* = 133 patients) with a polyhandicap (IQ < 20 and GMFCS IV or V). The most frequent comorbidities included chronic respiratory insufficiency, chronic digestive problems, pulmonary infection, epilepsy, and scoliosis. Poor oral hygiene status and severe behavioural problems were less detected. These results are in line with our study. In our analysis, GERD and visual impairment were highly prevalent, but this was not reported in the article by Rousseau et al.

Van Timmeren et al. [46] systematically reviewed health problems in people with SPIMD (*n* = 20 studies and 3177 patients). Their study population consisted of adults. The high prevalence of epilepsy, visual impairment, and dysphagia in their study is consistent with our results. Hearing impairment was described in both the study of Van Timmeren et al. and our study. In our study, GERD was more reported compared to Van Timmeren et al. We hypothesize that this could be related to our age group.

Our results aligned closely with the results of Nelson et al. [47]. The population in their study consisted of children (*n* = 102 studies) with severe neurological impairment (SNI). PIMD can be interpreted as a subgroup of SNI. The most reported problem in this study was GERD/vomiting, while epilepsy was the problem with the highest prevalence in our study. Skin and cardiac problems were not frequently reported in the study of Nelson et al. or our own study. Van Timmeren et al. [46] found that 16 in a population of 254 adults had a congenital heart defect, while Nelson et al. [47] found congenital heart defects in three different studies on children.

Interesting additional results could be extracted from a Delphi study about research priorities for children with neurological impairment and medical complexity [48]. Caregivers prioritized behaviour, acute lower respiratory tract infections, and enteral feeding tubes, while clinicians prioritized irritability and pain, mental health, and disorders of tone. However, these topics were not the most often reported in our results. The most reported item in our study, epilepsy, was not a clinical topic of prioritization for either caregivers or clinicians. Infection control was also more highly prioritized for caregivers than for clinicians.

What is striking is the fact that we could find only 17 articles published in the period 2005–2023, while 5 articles published in the last 1.5 years were found. It seems that children with PIMD have received more scientific attention in the last few years.

### Strength and limitations

To our knowledge, this is the first review specifically conducted to assess health problems in children with PIMD. Our study’s detailed search strategy, which included a broad range of search terms, including older terminology, was one of its strengths. The most thorough search was conducted using five databases with the assistance of an information specialist from the library of the academic medical university. Consequently, our initial search produced a large number of results. Two reviewers independently assessed the full text of the articles.

The most important limitation in our search was the definition and terminology of PIMD. The definition and terminology of PIMD were not uniform in the scientific literature. Therefore, a very broad search strategy was needed. However, due to the lack of uniform terminology, there was still a chance that relevant publications were overlooked. For SNI, a Delphi consensus-based definition was established [49]. A consensus definition would similarly be conducive to PIMD. Children with PIMD are a very specific population with different causes for their impairments; they do not have one etiological diagnosis, as PIMD is a “care diagnosis.”

One reviewer screened most of the abstracts, which could be a limitation. However, at the beginning, the reviewer screened together with the consulting second reviewer to gain a sense of the process. Also, if necessary, abstracts were discussed with a consulting second reviewer throughout the entire screening process. Another limitation was that the quality of studies included in this scoping review was not extensively assessed, though the selection of studies from only peer-reviewed journals suggests that we should be fine with the quality of the studies. However, we are aware of the effect it could have on the validity of the findings.

We also considered the age of health problem onset and the treatment. It was difficult to interpret from the scarce information on treatment whether this is related to PIMD-specific health problems or etiological diagnoses. Therefore, there is so little information available; no inferences can be made. An explanation could be that the population is heterogeneous, which makes it challenging to find information on specific problems.

### Further research

Based on our findings, it is highly desirable that there should be more studies on health problems, the age of onset of these health problems, and the appropriate treatments for children with PIMD. To improve the accessibility of research on children/people with PIMD, we recommend using a uniform definition and terminology for this patient group.

Behaviour was not an item in our search strategy; however, it was included in the extraction form. We can imagine that if this had been included in the search strategy, we would have found more articles. It is advisable to include this in further research.

The goal of our research was to gather reliable data for establishing a multidisciplinary care pathway by creating a patient journey that ensures that healthcare professionals are involved when needed and that a good diagnostic workup is used. Further research on this is still necessary, as a concrete patient journey has not yet been found.

## Conclusions

This scoping review investigated the health problems of children with PIMD. The main reported problems in children with PIMD were epilepsy, respiratory infections, feeding difficulties, GERD, scoliosis, and visual impairment. Awareness, early detection, and treatment of the health problems could optimize the medical care and quality of life of children with PIMD. To conclude, this review demonstrated the urgent need for additional systematic research on health problems in children with PIMD.

## Supplementary Information

Below is the link to the electronic supplementary material.

Supplementary file1 (DOCX 32 KB)

Supplementary file2 (DOCX 33 KB)

Supplementary file3 (DOCX 44 KB)

## Data Availability

No datasets were generated or analysed during the current study.

## Abbreviations

DQ: Developmental quotient
IQ: Intellectual quotient
PIMD: Profound intellectual and multiple disabilities
GERD: Gastroesophageal reflux disease
GMFCS: Gross motor function classification system
SPIMD: Severe or profound intellectual and motor disabilities
