# Supplementary material for: Health problems in children with profound intellectual and multiple disabilities: a scoping review
Source: Eur J Pediatr. 2024 Dec 6;184(1):67. doi: 10.1007/s00431-024-05876-x (PMC11624250; doi:10.1007/s00431-024-05876-x)
Supplement: Supplementary file 2 — Supplementary file2 (DOCX 33 KB) [file 431_2024_5876_MOESM2_ESM.docx]

**Appendix 2: Study Protocol**

**Background**

Children with profound intellectual and multiple disabilities (PIMD) have severe intellectual and motor disabilities. These children have intensive needs for care and generally visit the hospital more often than peers without PIMD [1]. In this study, PIMD is defined as a motor disability classified as gross motor function classification system (GMFCS) IV or V, and intellectual disability classified as IQ <30 or developmental age < 2 years [2]. Different etiological origins can be the cause of the disabilities, divided into genetic, metabolic, acquired, and neurologic causes [3]. Health problems that occur in these children consist of pulmonary/respiratory problems, dysphagia, epilepsy, spasticity, hearing and visual problems, gastrointestinal problems, and cardiovascular problems [4]. Due to the variety and complexity of medical conditions and health problems, different (sub)specialties and paramedics are involved. Therefore, the care for children with PIMD is fragmented. As a result, it is not always clear who coordinates the care of these children [5].

Families with a child with PIMD struggle with the strain of frequent hospital appointments. Earlier research showed that children with PIMD have many outpatient clinic appointments, namely an average of 18.4 per year compared to 3.7 in the control group. In addition, they were admitted longer compared to peers without PIMD, namely 12.0 compared to 6.8 days [1]. In addition to the emotional strain, there are also financial consequences [6]. For example, parents may have to take time off work, arrange transportation and provide care for any other children. This puts a heavy burden on both the child and the system.

Due to the complexity of their needs, children with PIMD require a comprehensive approach to care. Early detection of physical health issues is essential to improve or sustain health and quality of life [7]. Proactive care is needed to prevent unscheduled outpatient care appointments and hospitalizations [8]**.** Health problems remaining undiagnosed and untreated may lead to developing secondary health problems [9]. Therefore, screening for health problems at the right age is important.

**Aim**

This study aims to provide scientific depth on medical outpatient care for children with PIMD. This scoping review will lead to the possibility of organizing integrated proactive care for children with intensive needs for care.

**Main question**

- Which health problems occur in children with PIMD?

Sub questions

- At what age do medical problems arise and how do these symptoms develop in terms of severity and progress?
- What is known about the treatment of these medical problems in children with PIMD?
- Does our definition of PIMD correspond to the definition found in the literature?

**Study design**

Scoping review of medical problems described in research about children/people with profound intellectual and multiple disabilities.

**Eligibility criteria**

Participants

- Inclusion
  - PIMD
  - ID and motor impairment
  - Age between 0-18 years
- Exclusion
  - Age > 18 years

Concept

- Occurrence and course of medical problems
  - Natural course (by age)
- Treatment information

Context

- Outpatient clinic care
- Hospitalisation
- Care system

Outcome

- Cardiac
- Respiratory
- Oropharyngeal
- Infectious
- Gastro-intestinal
- Neurologic
- Musculoskeletal
- Psychological (behavioural)
- Skin and sensory disorders

Type of sources

This scoping review will consider both experimental and quasi-experimental study designs including randomized controlled trials, non-randomized controlled trials, before and after studies and interrupted time-series studies. In addition, analytical observational studies including prospective and retrospective cohort studies, case-control studies and analytical cross-sectional studies will be considered for inclusion. This review will also consider descriptive observational study designs including case series, individual case reports and descriptive cross-sectional studies for inclusion.

**Methods**

Search strategy

Databases for search are PubMed, MEDLINE, Embase, Web of Science and PsychInfo. Studies published in English and Dutch will be included.

**Study/source of evidence selection**

Following the search, all identified citations will be collated and uploaded into Endnote and duplicates removed. The citations will be uploaded in Rayyan to screen titles and abstract by one reviewer. The full text of the selected citations will be screened by two independent reviewers. Reasons for exclusion of sources of evidence at full text that do not meet the inclusion criteria will be recorded and reported in the scoping review. Any disagreements that arise between the reviewers at each stage of the selection process will be resolved through discussion with an additional reviewer. The results of the search and the study inclusion process will be reported in full in the final scoping review and presented in a Preferred Reporting Items for Systematic Reviews and Meta-analyses extension for scoping review (PRISMA-ScR) flow diagram.

**Data collection form/data extraction**

| **General information** |  |
| --- | --- |
| Study ID |  |
| Last name of first author |  |
| Year of publication |  |
| Title |  |
| Country in which the study was conducted |  |
| Study subcategory |  |
| What does the study call PIMD/ID? |  |
| **Methods** |  |
| Aim of the study/purpose |  |
| Study design |  |
| Study population and sample size (ia) |  |
| Intervention type/duration, comparator, outcome measures (ia) |  |
| Data source (chart review, prospective clinical data, parent/caregiver, clinical trials) |  |
| **Findings** |  |
| Physical health problems |  |
| Definition |  |
| Treatment options |  |
| Age of onset |  |

**Implication**

This study will fill a gap in current research by aiming to describe the health problems in children with PIMD. A clear understanding of the patient journey can pave way for future research efforts.

**References**

1. van Lienden R. De zorg voor kinderen met ernstige meervoudige beperkingen in het Radboudumc Amalia Kinderziekenhuis in kaart gebracht. Bezit van Vakgroep Kindergeneekunde2020.

2. Nakken H, Vlaskamp C. A Need for a Taxonomy for Profound Intellectual and Multiple Disabilities. J Policy Pract Intellect Disabil. 2007;4(2):83-7.

3. Gehandicapten PEEM. Personen met EMB [Available from: [https://www.platformemg.nl/algemeen/over-emb/personen-met-emb/#](https://www.platformemg.nl/algemeen/over-emb/personen-met-emb/).

4. van Timmeren EA, van der Putten AA, van Schrojenstein Lantman-de Valk HM, van der Schans CP, Waninge A. Prevalence of reported physical health problems in people with severe or profound intellectual and motor disabilities: a cross-sectional study of medical records and care plans. J Intellect Disabil Res. 2016;60(11):1109-18.

5. Daamen JC, Derksen-Lubsen G, Rake JP. Netwerkzorg voor kinderen met een complexe zorgbehoefte. Praktische pediatrie. 2018;12(4).

6. Seliner B, Latal B, Spirig R. When children with profound multiple disabilities are hospitalized: A cross-sectional survey of parental burden of care, quality of life of parents and their hospitalized children, and satisfaction with family-centered care. J Spec Pediatr Nurs. 2016;21(3):147-57.

7. Robertson J, Hatton C, Emerson E, Baines S. The impact of health checks for people with intellectual disabilities: an updated systematic review of evidence. Res Dev Disabil. 2014;35(10):2450-62.

8. Durbin J, Selick A, Casson I, Green L, Spassiani N, Perry A, Lunsky Y. Evaluating the Implementation of Health Checks for Adults With Intellectual and Developmental Disabilities in Primary Care: The Importance of Organizational Context. Intellect Dev Disabil. 2016;54(2):136-50.

9. May ME, Kennedy CH. Health and problem behavior among people with intellectual disabilities. Behav Anal Pract. 2010;3(2):4-12.
