# Supplementary material for: Health problems in children with profound intellectual and multiple disabilities: a scoping review
Source: Eur J Pediatr. 2024 Dec 6;184(1):67. doi: 10.1007/s00431-024-05876-x (PMC11624250; doi:10.1007/s00431-024-05876-x)
Supplement: Supplementary file 3 — Supplementary file3 (DOCX 44 KB) [file 431_2024_5876_MOESM3_ESM.docx]

**Appendix 3: Search strategies**

**Embase Search**

((Complex adj2 **developmental abnormalit**y).ti,ab,kf. OR (Multiple adj2 developmental abnormality).ti,ab,kf. OR (Profound adj2 developmental abnormality).ti,ab,kf. OR (Serious adj2 developmental abnormality).ti,ab,kf. OR (Severe adj2 developmental abnormality).ti,ab,kf. OR (Complex adj2 **developmental abnormalities**).ti,ab,kf. OR (Multiple adj2 developmental abnormalities).ti,ab,kf. OR (Profound adj2 developmental abnormalities).ti,ab,kf. OR (Serious adj2 developmental abnormalities).ti,ab,kf. OR (Severe adj2 developmental abnormalities).ti,ab,kf. OR (**Complex adj2 intellectual abnormality**).ti,ab,kf. OR (Multiple adj2 intellectual abnormality).ti,ab,kf. OR (Profound adj2 intellectual abnormality).ti,ab,kf. OR (Serious adj2 intellectual abnormality).ti,ab,kf. OR (Severe adj2 intellectual abnormality).ti,ab,kf. OR (**Complex adj2 intellectual abnormalities**).ti,ab,kf. OR (Multiple adj2 intellectual abnormalities).ti,ab,kf. OR (Profound adj2 intellectual abnormalities).ti,ab,kf. OR (Serious adj2 intellectual abnormalities).ti,ab,kf. OR (Severe adj2 intellectual abnormalities).ti,ab,kf. OR (Complex adj2 **mental abnormality**).ti,ab,kf. OR (Multiple adj2 mental abnormality).ti,ab,kf. OR (Profound adj2 mental abnormality).ti,ab,kf. OR (Serious adj2 mental abnormality).ti,ab,kf. OR (Severe adj2 mental abnormality).ti,ab,kf. OR (Complex adj2 **mental abnormalities**).ti,ab,kf. OR (Multiple adj2 mental abnormalities).ti,ab,kf. OR (Profound adj2 mental abnormalities).ti,ab,kf. OR (Serious adj2 mental abnormalities).ti,ab,kf. OR (Severe adj2 mental abnormalities).ti,ab,kf. OR (Complex adj2 **neurodevelopmental abnormality**).ti,ab,kf. OR (Multiple adj2 neurodevelopmental abnormality).ti,ab,kf. OR (Profound adj2 neurodevelopmental abnormality).ti,ab,kf. OR (Serious adj2 neurodevelopmental abnormality).ti,ab,kf. OR (Severe adj2 neurodevelopmental abnormality).ti,ab,kf. OR (Complex adj2 **neurodevelopmental abnormalities**).ti,ab,kf. OR (Multiple adj2 neurodevelopmental abnormalities).ti,ab,kf. OR (Profound adj2 neurodevelopmental abnormalities).ti,ab,kf. OR (Serious adj2 neurodevelopmental abnormalities).ti,ab,kf. OR (Severe adj2 neurodevelopmental abnormalities).ti,ab,kf. OR (Complex adj2 **cognitive defect**).ti,ab,kf. OR (Multiple adj2 cognitive defect).ti,ab,kf. OR (Profound adj2 cognitive defect).ti,ab,kf. OR (Serious adj2 cognitive defect).ti,ab,kf. OR (Severe adj2 cognitive defect).ti,ab,kf. OR (Complex adj2 **cognitive defect**s).ti,ab,kf. OR (Multiple adj2 cognitive defects).ti,ab,kf. OR (Profound adj2 cognitive defects).ti,ab,kf. OR (Serious adj2 cognitive defects).ti,ab,kf. OR (Severe adj2 cognitive defects).ti,ab,kf. OR (Complex adj2 **developmental defect**).ti,ab,kf. OR (Multiple adj2 developmental defect).ti,ab,kf. OR (Profound adj2 developmental defect).ti,ab,kf. OR (Serious adj2 developmental defect).ti,ab,kf. OR (Severe adj2 developmental defect*).ti,ab,kf. OR (Complex adj2 **developmental defects**).ti,ab,kf. OR (Multiple adj2 developmental defects).ti,ab,kf. OR (Profound adj2 developmental defects).ti,ab,kf. OR (Serious adj2 developmental defects).ti,ab,kf. OR (Severe adj2 developmental defects).ti,ab,kf. OR (Complex adj2 **intellectual defect**).ti,ab,kf. OR (Multiple adj2 intellectual defect).ti,ab,kf. OR (Profound adj2 intellectual defect).ti,ab,kf. OR (Serious adj2 intellectual defect).ti,ab,kf. OR (Severe adj2 intellectual defect).ti,ab,kf. OR (Complex adj2 **intellectual defects**).ti,ab,kf. OR (Multiple adj2 intellectual defects).ti,ab,kf. OR (Profound adj2 intellectual defects).ti,ab,kf. OR (Serious adj2 intellectual defects).ti,ab,kf. OR (Severe adj2 intellectual defects).ti,ab,kf. OR (Complex adj2 **mental defect**).ti,ab,kf. OR (Multiple adj2 mental defect).ti,ab,kf. OR (Profound adj2 mental defect).ti,ab,kf. OR (Serious adj2 mental defect).ti,ab,kf. OR (Severe adj2 mental defect).ti,ab,kf. OR (Complex adj2 **mental defects**).ti,ab,kf. OR (Multiple adj2 mental defects).ti,ab,kf. OR (Profound adj2 mental defects).ti,ab,kf. OR (Serious adj2 mental defects).ti,ab,kf. OR (Severe adj2 mental defects).ti,ab,kf. OR (Complex adj2 **neurodevelopmental defect**).ti,ab,kf. OR (Multiple adj2 neurodevelopmental defect).ti,ab,kf. OR (Profound adj2 neurodevelopmental defect).ti,ab,kf. OR (Serious adj2 neurodevelopmental defect).ti,ab,kf. OR (Severe adj2 neurodevelopmental defect).ti,ab,kf. OR (Complex adj2 **neurodevelopmental defect**s).ti,ab,kf. OR (Multiple adj2 neurodevelopmental defects).ti,ab,kf. OR (Profound adj2 neurodevelopmental defects).ti,ab,kf. OR (Serious adj2 neurodevelopmental defects).ti,ab,kf. OR (Severe adj2 neurodevelopmental defects).ti,ab,kf. OR (Multiply adj2 **cognitively defective**).ti,ab,kf. OR (Profoundly adj2 cognitively defective).ti,ab,kf. OR (Seriously adj2 cognitively defective).ti,ab,kf. OR (Severely adj2 cognitively defective).ti,ab,kf. OR (Multiply adj2 **developmentally defective**).ti,ab,kf. OR (Profoundly adj2 developmentally defective).ti,ab,kf. OR (Seriously adj2 developmentally defective).ti,ab,kf. OR (Severely adj2 developmentally defective).ti,ab,kf. OR (Multiply adj2 **intellectually defective**).ti,ab,kf. OR (Profoundly adj2 intellectually defective).ti,ab,kf. OR (Seriously adj2 intellectually defective).ti,ab,kf. OR (Severely adj2 intellectually defective).ti,ab,kf. OR (Profoundly adj2 **mentally defective**).ti,ab,kf. OR (Seriously adj2 mentally defective).ti,ab,kf. OR (Severely adj2 mentally defective).ti,ab,kf. OR (Multiply adj2 **neurodevelopmentally defectiv**e).ti,ab,kf. OR (Profoundly adj2 neurodevelopmentally defective).ti,ab,kf. OR (Seriously adj2 neurodevelopmentally defective).ti,ab,kf. OR (Severely adj2 neurodevelopmentally defective).ti,ab,kf. OR (Complex adj2 **cognitive deficienc***).ti,ab,kf. OR (Multiple adj2 cognitive deficienc*).ti,ab,kf. OR (Profound adj2 cognitive deficienc*).ti,ab,kf. OR (Serious adj2 cognitive deficienc*).ti,ab,kf. OR (Severe adj2 cognitive deficienc*).ti,ab,kf. OR (Complex adj2 **developmental deficienc***).ti,ab,kf. OR (Multiple adj2 developmental deficienc*).ti,ab,kf. OR (Profound adj2 developmental deficienc*).ti,ab,kf. OR (Serious adj2 developmental deficienc*).ti,ab,kf. OR (Severe adj2 developmental deficienc*).ti,ab,kf. OR (Complex adj2 **intellectual deficienc***).ti,ab,kf. OR (Multiple adj2 intellectual deficienc*).ti,ab,kf. OR (Profound adj2 intellectual deficienc*).ti,ab,kf. OR (Serious adj2 intellectual deficienc*).ti,ab,kf. OR (Severe adj2 intellectual deficienc*).ti,ab,kf. OR (Complex adj2 **mental deficienc***).ti,ab,kf. OR (Multiple adj2 mental deficienc*).ti,ab,kf. OR (Profound adj2 mental deficienc*).ti,ab,kf. OR (Serious adj2 mental deficienc*).ti,ab,kf. OR (Severe adj2 mental deficienc*).ti,ab,kf. OR (Complex adj2 **neurodevelopmental deficienc***).ti,ab,kf. OR (Multiple adj2 neurodevelopmental deficienc*).ti,ab,kf. OR (Profound adj2 neurodevelopmental deficienc*).ti,ab,kf. OR (Serious adj2 neurodevelopmental deficienc*).ti,ab,kf. OR (Severe adj2 neurodevelopmental deficienc*).ti,ab,kf. OR (Multiply adj2 **cognitively deficient**).ti,ab,kf. OR (Profoundly adj2 cognitively deficient).ti,ab,kf. OR (Seriously adj2 cognitively deficient).ti,ab,kf. OR (Severely adj2 cognitively deficient).ti,ab,kf. OR (Multiply adj2 **developmentally deficient).ti,ab,kf. OR (**Profoundly adj2 developmentally deficient).ti,ab,kf. OR (Seriously adj2 developmentally deficient).ti,ab,kf. OR (Severely adj2 developmentally deficient).ti,ab,kf. OR (Multiply adj2 **intellectually deficient**).ti,ab,kf. OR (Profoundly adj2 intellectually deficient).ti,ab,kf. OR (Seriously adj2 intellectually deficient).ti,ab,kf. OR (Severely adj2 intellectually deficient).ti,ab,kf. OR (Multiply adj2 **mentally deficient**).ti,ab,kf. OR (Profoundly adj2 mentally deficient).ti,ab,kf. OR (Seriously adj2 mentally deficient).ti,ab,kf. OR (Severely adj2 mentally deficient).ti,ab,kf. OR (Multiply adj2 **neurodevelopmentally deficient**).ti,ab,kf. OR (Profoundly adj2 neurodevelopmentally deficient).ti,ab,kf. OR (Seriously adj2 neurodevelopmentally deficient).ti,ab,kf. OR (Severely adj2 neurodevelopmentally deficient).ti,ab,kf. OR (**Complex adj2 developmental deficit***).ti,ab,kf. OR (Multiple adj2 developmental deficit*).ti,ab,kf. OR (Profound adj2 developmental deficit*).ti,ab,kf. OR (Serious adj2 developmental deficit*).ti,ab,kf. OR (Severe adj2 developmental deficit*).ti,ab,kf. OR (Complex adj2 **intellectual deficit***).ti,ab,kf. OR (Multiple adj2 intellectual deficit*).ti,ab,kf. OR (Profound adj2 intellectual deficit*).ti,ab,kf. OR (Serious adj2 intellectual deficit*).ti,ab,kf. OR (Severe adj2 intellectual deficit*).ti,ab,kf. OR (Complex adj2 **mental deficit*).ti,ab,kf. OR (**Multiple adj2 mental deficit*).ti,ab,kf. OR (Profound adj2 mental deficit*).ti,ab,kf. OR (Serious adj2 mental deficit*).ti,ab,kf. OR (Severe adj2 mental deficit*).ti,ab,kf.

OR (Complex adj2 **neurodevelopmental deficit***).ti,ab,kf. OR (Multiple adj2 neurodevelopmental deficit*).ti,ab,kf. OR (Profound adj2 neurodevelopmental deficit*).ti,ab,kf. OR (Serious adj2 neurodevelopmental deficit*).ti,ab,kf. OR (Severe adj2 neurodevelopmental deficit*).ti,ab,kf. OR (Complex adj2 **cognitive delay**).ti,ab,kf. OR (Multiple adj2 cognitive delay).ti,ab,kf. OR (Profound adj2 cognitive delay).ti,ab,kf. OR (Serious adj2 cognitive delay).ti,ab,kf. OR (Severe adj2 cognitive delay).ti,ab,kf. OR (Complex adj2 **developmental delay**).ti,ab,kf. OR (Multiple adj2 developmental delay).ti,ab,kf. OR (Profound adj2 developmental delay).ti,ab,kf. OR (Serious adj2 developmental delay).ti,ab,kf. OR (Severe adj2 developmental delay).ti,ab,kf. OR (Complex adj2 **intellectual delay**).ti,ab,kf. OR (Multiple adj2 intellectual delay).ti,ab,kf. OR (Profound adj2 intellectual delay).ti,ab,kf. OR (Serious adj2 intellectual delay).ti,ab,kf. OR (Severe adj2 intellectual delay).ti,ab,kf. OR (Complex adj2 **mental delay**).ti,ab,kf. OR (Multiple adj2 mental delay).ti,ab,kf. OR (Profound adj2 mental delay).ti,ab,kf. OR (Serious adj2 mental delay).ti,ab,kf. OR (Severe adj2 mental delay).ti,ab,kf. OR (Complex adj2 **neurodevelopmental delay**).ti,ab,kf. OR (Multiple adj2 neurodevelopmental delay).ti,ab,kf. OR (Profound adj2 neurodevelopmental delay).ti,ab,kf. OR (Serious adj2 neurodevelopmental delay).ti,ab,kf. OR (Severe adj2 neurodevelopmental delay).ti,ab,kf. OR (Multiply adj2 **cognitively delayed**).ti,ab,kf. OR (Profoundly adj2 cognitively delayed).ti,ab,kf. OR (Seriously adj2 cognitively delayed).ti,ab,kf. OR (Severely adj2 cognitively delayed).ti,ab,kf. OR (Multiply adj2 **developmentally delayed**).ti,ab,kf. OR (Profoundly adj2 developmentally delayed).ti,ab,kf. OR (Seriously adj2 developmentally delayed).ti,ab,kf. OR (Severely adj2 developmentally delayed).ti,ab,kf. OR (Multiply adj2 **intellectually delayed**).ti,ab,kf. OR (Profoundly adj2 intellectually delayed).ti,ab,kf. OR (Seriously adj2 intellectually delayed).ti,ab,kf. OR (Severely adj2 intellectually delayed).ti,ab,kf. OR (Multiply adj2 **mentally delayed).ti,ab,kf. OR (**Profoundly adj2 mentally delayed).ti,ab,kf. OR (Seriously adj2 mentally delayed).ti,ab,kf. OR (Severely adj2 mentally delayed).ti,ab,kf. OR (Multiply adj2 **neurodevelopmentally delayed).ti,ab,kf. OR (**Profoundly adj2 neurodevelopmentally delayed).ti,ab,kf. OR (Seriously adj2 neurodevelopmentally delayed).ti,ab,kf. OR (Severely adj2 neurodevelopmentally delayed).ti,ab,kf. OR (Multiply adj2 **differently-abled**).ti,ab,kf. OR (Profoundly adj2 differently-abled).ti,ab,kf. OR (Seriously adj2 differently-abled).ti,ab,kf. OR (Severely adj2 differently-abled).ti,ab,kf. OR (Multiple adj2 **developmental difficult**y).ti,ab,kf. OR (Complex adj2 developmental difficulty).ti,ab,kf. OR (Profound adj2 developmental difficulty).ti,ab,kf. OR (Serious adj2 developmental difficulty).ti,ab,kf. OR (Severe adj2 developmental difficulty).ti,ab,kf. OR (Multiple adj2 **developmental difficult**ies).ti,ab,kf. OR (Complex adj2 developmental difficulties).ti,ab,kf. OR (Profound adj2 developmental difficulties).ti,ab,kf. OR (Serious adj2 developmental difficulties).ti,ab,kf. OR (Severe adj2 developmental difficulties).ti,ab,kf. OR (**Multiple adj2 intellectual difficulty**).ti,ab,kf. OR (Complex adj2 intellectual difficulty).ti,ab,kf. OR (Profound adj2 intellectual difficulty).ti,ab,kf. OR (Serious adj2 intellectual difficulty).ti,ab,kf. OR (Severe adj2 intellectual difficulty).ti,ab,kf. OR (**Multiple adj2 intellectual difficulties**).ti,ab,kf. OR (Complex adj2 intellectual difficulties).ti,ab,kf. OR (Profound adj2 intellectual difficulties).ti,ab,kf. OR (Serious adj2 intellectual difficulties).ti,ab,kf. OR (Severe adj2 intellectual difficulties).ti,ab,kf. OR (Multiple adj2 **mental difficulty**).ti,ab,kf. OR (Complex adj2 mental difficulty).ti,ab,kf. OR (Profound adj2 mental difficulty).ti,ab,kf. OR (Serious adj2 mental difficulty).ti,ab,kf. OR (Severe adj2 mental difficulty).ti,ab,kf. OR (Multiple adj2 **mental difficulties**).ti,ab,kf. OR (Complex adj2 mental difficulties).ti,ab,kf. OR (Profound adj2 mental difficulties).ti,ab,kf. OR (Serious adj2 mental difficulties).ti,ab,kf. OR (Severe adj2 mental difficulties).ti,ab,kf. OR (Complex adj2 **neurodevelopmental difficulty**).ti,ab,kf. OR (Multiple adj2 neurodevelopmental difficulty).ti,ab,kf. OR (Profound adj2 neurodevelopmental difficulty).ti,ab,kf. OR (Serious adj2 neurodevelopmental difficulty).ti,ab,kf. OR (Severe adj2 neurodevelopmental difficulty).ti,ab,kf. OR (Complex adj2 **neurodevelopmental difficulties**).ti,ab,kf. OR (Multiple adj2 neurodevelopmental difficulties).ti,ab,kf. OR (Profound adj2 neurodevelopmental difficulties).ti,ab,kf. OR (Serious adj2 neurodevelopmental difficulties).ti,ab,kf. OR (Severe adj2 neurodevelopmental difficulties).ti,ab,kf. OR (Complex adj2 **cognitive disability**).ti,ab,kf. OR (Multiple adj2 cognitive disability).ti,ab,kf. OR (Profound adj2 cognitive disability).ti,ab,kf. OR (Serious adj2 cognitive disability).ti,ab,kf. OR (Severe adj2 cognitive disability).ti,ab,kf. OR (Complex adj2 **cognitive disabilities**).ti,ab,kf. OR (Multiple adj2 cognitive disabilities).ti,ab,kf. OR (Profound adj2 cognitive disabilities).ti,ab,kf. OR (Serious adj2 cognitive disabilities).ti,ab,kf. OR (Severe adj2 cognitive disabilities).ti,ab,kf. OR (Complex adj2 **developmental disability**).ti,ab,kf. OR (Multiple adj2 developmental disability).ti,ab,kf. OR (Multiple adj2 developmental disability).ti,ab,kf. OR (Serious adj2 developmental disability).ti,ab,kf. OR (Severe adj2 developmental disability).ti,ab,kf. OR (Complex adj2 **developmental disabilities**).ti,ab,kf. OR (Multiple adj2 developmental disabilities).ti,ab,kf. OR (Multiple adj2 developmental disabilities).ti,ab,kf. OR (Serious adj2 developmental disabilities).ti,ab,kf. OR (Severe adj2 developmental disabilities).ti,ab,kf. OR (Trainable adj2 **intellectual disabilit**y).ti,ab,kf. OR (Complex adj2 intellectual disability).ti,ab,kf. OR (Multiple adj2 intellectual disability).ti,ab,kf. OR (Profound adj2 intellectual disability).ti,ab,kf. OR (Serious adj2 intellectual disability).ti,ab,kf. OR (Severe adj2 intellectual disability).ti,ab,kf. OR (Trainable adj2 **intellectual disabilit**ies).ti,ab,kf. OR (Complex adj2 intellectual disabilities).ti,ab,kf. OR (Multiple adj2 intellectual disabilities).ti,ab,kf. OR (Profound adj2 intellectual disabilities).ti,ab,kf. OR (Serious adj2 intellectual disabilities).ti,ab,kf. OR (Severe adj2 intellectual disabilities).ti,ab,kf. OR (**Severe adj2 Profound adj2 intellectual motor disability**).ti,ab,kf. OR (**Severe adj2 Profound adj2 intellectual motor disabilities)**.ti,ab,kf. OR (Complex adj2 **mental disabilit**y).ti,ab,kf. OR (Multiple adj2 mental disability).ti,ab,kf. OR (Profound adj2 mental disability).ti,ab,kf. OR (Serious adj2 mental disability).ti,ab,kf. OR (Severe adj2 mental disability).ti,ab,kf. OR (Trainable adj2 mental disability).ti,ab,kf. OR (Complex adj2 **mental disabilit**ies).ti,ab,kf. OR (Multiple adj2 mental disabilities).ti,ab,kf. OR (Profound adj2 mental disabilities).ti,ab,kf. OR (Serious adj2 mental disabilities).ti,ab,kf. OR (Severe adj2 mental disabilities).ti,ab,kf. OR (Trainable adj2 mental disabilities).ti,ab,kf. OR (Complex adj2 **neurodevelopmental disabilit**y).ti,ab,kf. OR (Multiple adj2 neurodevelopmental disability).ti,ab,kf. OR (Profound adj2 neurodevelopmental disability).ti,ab,kf. OR (Serious adj2 neurodevelopmental disability).ti,ab,kf. OR (Severe adj2 neurodevelopmental disability).ti,ab,kf. OR (Complex adj2 **neurodevelopmental disabilit**ies).ti,ab,kf. OR (Multiple adj2 neurodevelopmental disabilities).ti,ab,kf. OR (Profound adj2 neurodevelopmental disabilities).ti,ab,kf. OR (Serious adj2 neurodevelopmental disabilities).ti,ab,kf. OR (Severe adj2 neurodevelopmental disabilities).ti,ab,kf. OR (Multiple adj2 disability).ti,ab,kf. OR (Multiple adj2 disabilities).ti,ab,kf. OR (Multiply adj2 **cognitively disabled**).ti,ab,kf. OR (Profoundly adj2 cognitively disabled).ti,ab,kf. OR (Seriously adj2 cognitively disabled).ti,ab,kf. OR (Severely adj2 cognitively disabled).ti,ab,kf. OR (Multiply adj2 **developmentally disabled**).ti,ab,kf. OR (Profoundly adj2 developmentally disabled).ti,ab,kf. OR (Seriously adj2 developmentally disabled).ti,ab,kf. OR (Severely adj2 developmentally disabled).ti,ab,kf. OR (Trainable adj2 developmentally disabled).ti,ab,kf. OR (Multiply adj2 **intellectually disabled**).ti,ab,kf. OR (Profoundly adj2 intellectually disabled).ti,ab,kf. OR (Seriously adj2 intellectually disabled).ti,ab,kf. OR (Severely adj2 intellectually disabled).ti,ab,kf. OR (Trainable adj2 intellectually disabled).ti,ab,kf. OR (Multiply adj2 **mentally disabled**).ti,ab,kf. OR (Profoundly adj2 mentally disabled).ti,ab,kf. OR (Seriously adj2 mentally disabled).ti,ab,kf. OR (Severely adj2 mentally disabled).ti,ab,kf. OR (Multiply adj2 **neurodevelopmentally disabled**).ti,ab,kf. OR (Profoundly adj2 neurodevelopmentally disabled).ti,ab,kf. OR (Seriously adj2 neurodevelopmentally disabled).ti,ab,kf. OR (Severely adj2 neurodevelopmentally disabled).ti,ab,kf. OR (Multiply adj2 disabled).ti,ab,kf. OR (Trainable adj2 **mentally disabled)**.ti,ab,kf. OR (Complex adj2 **developmental disorder***).ti,ab,kf. OR (Multiple adj2 developmental disorder*).ti,ab,kf. OR (Profound adj2 developmental disorder*).ti,ab,kf. OR (Serious adj2 developmental disorder*).ti,ab,kf. OR (Severe adj2 developmental disorder*).ti,ab,kf. OR (Complex adj2 **intellectual disorder**).ti,ab,kf. OR (Multiple adj2 intellectual disorder).ti,ab,kf. OR (Profound adj2 intellectual disorder).ti,ab,kf. OR (Serious adj2 intellectual disorder).ti,ab,kf. OR (Severe adj2 intellectual disorder).ti,ab,kf. OR (Complex adj2 **intellectual disorders**).ti,ab,kf. OR (Multiple adj2 intellectual disorders).ti,ab,kf. OR (Profound adj2 intellectual disorders).ti,ab,kf. OR (Serious adj2 intellectual disorders).ti,ab,kf. OR (Severe adj2 intellectual disorders).ti,ab,kf. OR (Complex adj2 **neurodevelopmental disorder**).ti,ab,kf. OR (Multiple adj2 neurodevelopmental disorder).ti,ab,kf. OR (Profound adj2 neurodevelopmental disorder).ti,ab,kf. OR (Serious adj2 neurodevelopmental disorder).ti,ab,kf. OR (Severe adj2 neurodevelopmental disorder).ti,ab,kf. OR (Complex adj2 **neurodevelopmental disorder**s).ti,ab,kf. OR (Multiple adj2 neurodevelopmental disorders).ti,ab,kf. OR (Profound adj2 neurodevelopmental disorders).ti,ab,kf. OR (Serious adj2 neurodevelopmental disorders).ti,ab,kf. OR (Severe adj2 neurodevelopmental disorders).ti,ab,kf. OR (Complex adj2 **cognitive handicap***).ti,ab,kf. OR (Multiple adj2 cognitive handicap*).ti,ab,kf. OR (Profound adj2 cognitive handicap*).ti,ab,kf. OR (Serious adj2 cognitive handicap*).ti,ab,kf. OR (Severe adj2 cognitive handicap*).ti,ab,kf. OR (Complex adj2 **developmental handicap***).ti,ab,kf. OR (Multiple adj2 developmental handicap*).ti,ab,kf. OR (Profound adj2 developmental handicap*).ti,ab,kf. OR (Serious adj2 developmental handicap*).ti,ab,kf. OR (Severe adj2 developmental handicap*).ti,ab,kf. OR (Complex adj2 **intellectual handicap***).ti,ab,kf. OR (Multiple adj2 intellectual handicap*).ti,ab,kf. OR (Profound adj2 intellectual handicap*).ti,ab,kf. OR (Serious adj2 intellectual handicap*).ti,ab,kf. OR (Severe adj2 intellectual handicap*).ti,ab,kf. OR (Complex adj2 **mental handicap***).ti,ab,kf. OR (Multiple adj2 mental handicap*).ti,ab,kf. OR (Profound adj2 mental handicap*).ti,ab,kf. OR (Serious adj2 mental handicap*).ti,ab,kf. OR (Severe adj2 mental handicap*).ti,ab,kf. OR (Trainable adj2 mental handicap*).ti,ab,kf. OR (Complex adj2 **neurodevelopmental handicap***).ti,ab,kf. OR (Multiple adj2 neurodevelopmental handicap*).ti,ab,kf. OR (Profound adj2 neurodevelopmental handicap*).ti,ab,kf. OR (Serious adj2 neurodevelopmental handicap*).ti,ab,kf. OR (Severe adj2 neurodevelopmental handicap*).ti,ab,kf. OR (Multiply adj2 **cognitively handicapped**).ti,ab,kf. OR (Profoundly adj2 cognitively handicapped).ti,ab,kf. OR (Seriously adj2 cognitively handicapped).ti,ab,kf. OR (Severely adj2 cognitively handicapped).ti,ab,kf. OR (Multiply adj2 **developmentally handicapped**).ti,ab,kf. OR (Profoundly adj2 developmentally handicapped).ti,ab,kf. OR (Seriously adj2 developmentally handicapped).ti,ab,kf. OR (Severely adj2 developmentally handicapped).ti,ab,kf. OR (**Multiply adj2 intellectually handicapped**).ti,ab,kf. OR (Profoundly adj2 intellectually handicapped).ti,ab,kf. OR (Seriously adj2 intellectually handicapped).ti,ab,kf. OR (Severely adj2 intellectually handicapped).ti,ab,kf. OR (Multiply adj2 mentally handicapped).ti,ab,kf. OR (Profoundly adj2 **mentally handicapped**).ti,ab,kf. OR (Seriously adj2 mentally handicapped).ti,ab,kf. OR (Severely adj2 mentally handicapped).ti,ab,kf. OR (Multiply adj2 **neurodevelopmentally handicapped**).ti,ab,kf. OR (Profoundly adj2 neurodevelopmentally handicapped).ti,ab,kf. OR (Seriously adj2 neurodevelopmentally handicapped).ti,ab,kf. OR (Severely adj2 neurodevelopmentally handicapped).ti,ab,kf. OR (Multiply adj2 handicapped).ti,ab,kf. OR (Trainable adj2 mentally handicapped).ti,ab,kf. OR (Multiply adj2 **developmentally impaired**).ti,ab,kf. OR (Profoundly adj2 developmentally impaired).ti,ab,kf. OR (Seriously adj2 developmentally impaired).ti,ab,kf. OR (Severely adj2 developmentally impaired).ti,ab,kf. OR (Multiply adj2 **intellectually impaired**).ti,ab,kf. OR (Profoundly adj2 intellectually impaired).ti,ab,kf. OR (Seriously adj2 intellectually impaired).ti,ab,kf. OR (Severely adj2 intellectually impaired).ti,ab,kf. OR (Profoundly adj2 **mentally impaired**).ti,ab,kf. OR (Seriously adj2 mentally impaired).ti,ab,kf. OR (Severely adj2 mentally impaired).ti,ab,kf. OR (Multiply adj2 **neurodevelopmentally impaired**).ti,ab,kf. OR (Profoundly adj2 neurodevelopmentally impaired).ti,ab,kf. OR (Seriously adj2 neurodevelopmentally impaired).ti,ab,kf. OR (Severely adj2 neurodevelopmentally impaired).ti,ab,kf. OR (Trainable adj2 mentally impaired).ti,ab,kf. OR (Complex adj2 developmental impairment*).ti,ab,kf. OR (Multiple adj2 **developmental impairment***).ti,ab,kf. OR (Profound adj2 developmental impairment*).ti,ab,kf. OR (Serious adj2 developmental impairment*).ti,ab,kf. OR (Severe adj2 developmental impairment*).ti,ab,kf. OR (Complex adj2 **intellectual impairment***).ti,ab,kf. OR (Multiple adj2 intellectual impairment*).ti,ab,kf. OR (Profound adj2 intellectual impairment*).ti,ab,kf. OR (Serious adj2 intellectual impairment*).ti,ab,kf. OR (Severe adj2 intellectual impairment*).ti,ab,kf. OR (Complex adj2 **mental impairment***).ti,ab,kf. OR (Multiple adj2 mental impairment*).ti,ab,kf. OR (Profound adj2 mental impairment*).ti,ab,kf. OR (Serious adj2 mental impairment*).ti,ab,kf. OR (Severe adj2 mental impairment*).ti,ab,kf. OR (Complex adj2 **neurodevelopmental impairment***).ti,ab,kf. OR (Multiple adj2 neurodevelopmental impairment*).ti,ab,kf. OR (Profound adj2 neurodevelopmental impairment*).ti,ab,kf. OR (Serious adj2 neurodevelopmental impairment*).ti,ab,kf. OR (Severe adj2 neurodevelopmental impairment*).ti,ab,kf. OR (Trainable adj2 mental impairment*).ti,ab,kf. OR (Complex adj2 **cognitive incapacit***).ti,ab,kf. OR (Multiple adj2 cognitive incapacit*).ti,ab,kf. OR (Profound adj2 cognitive incapacit*).ti,ab,kf. OR (Serious adj2 cognitive incapacit*).ti,ab,kf. OR (Severe adj2 cognitive incapacit*).ti,ab,kf. OR (Complex adj2 **developmental incapacit***).ti,ab,kf. OR (Multiple adj2 developmental incapacit*).ti,ab,kf. OR (Profound adj2 developmental incapacit*).ti,ab,kf. OR (Serious adj2 developmental incapacit*).ti,ab,kf. OR (Severe adj2 developmental incapacit*).ti,ab,kf. OR (**Complex adj2 intellectual incapacit***).ti,ab,kf. OR (Multiple adj2 intellectual incapacit*).ti,ab,kf. OR (Profound adj2 intellectual incapacit*).ti,ab,kf. OR (Serious adj2 intellectual incapacit*).ti,ab,kf. OR (Severe adj2 intellectual incapacit*).ti,ab,kf. OR (Complex adj2 **mental incapacit***).ti,ab,kf. OR (Multiple adj2 mental incapacit*).ti,ab,kf. OR (Profound adj2 mental incapacit*).ti,ab,kf. OR (Serious adj2 mental incapacit*).ti,ab,kf. OR (Severe adj2 mental incapacit*).ti,ab,kf. OR (Complex adj2 **neurodevelopmental incapacit***).ti,ab,kf. OR (Multiple adj2 neurodevelopmental incapacit*).ti,ab,kf. OR (Profound adj2 neurodevelopmental incapacit*).ti,ab,kf. OR (Serious adj2 neurodevelopmental incapacit*).ti,ab,kf. OR (Severe adj2 neurodevelopmental incapacit*).ti,ab,kf. OR (Multiply adj2 **cognitively incapacitated**).ti,ab,kf. OR (Profoundly adj2 cognitively incapacitated).ti,ab,kf. OR (Seriously adj2 cognitively incapacitated).ti,ab,kf. OR (Severely adj2 cognitively incapacitated).ti,ab,kf. OR (Multiply adj2 **developmentally incapacitated**).ti,ab,kf. OR (Profoundly adj2 developmentally incapacitated).ti,ab,kf. OR (Seriously adj2 developmentally incapacitated).ti,ab,kf. OR (Severely adj2 developmentally incapacitated).ti,ab,kf. OR (Multiply adj2 **intellectually incapacitated**).ti,ab,kf. OR (Profoundly adj2 intellectually incapacitated).ti,ab,kf. OR (Seriously adj2 intellectually incapacitated).ti,ab,kf. OR (Severely adj2 intellectually incapacitated).ti,ab,kf. OR (Multiply adj2 **mentally incapacitated**).ti,ab,kf. OR (Profoundly adj2 mentally incapacitated).ti,ab,kf. OR (Seriously adj2 mentally incapacitated).ti,ab,kf. OR (Severely adj2 mentally incapacitated).ti,ab,kf. OR (Multiply adj2 **neurodevelopmentally incapacitated**).ti,ab,kf. OR (Profoundly adj2 neurodevelopmentally incapacitated).ti,ab,kf. OR (Seriously adj2 neurodevelopmentally incapacitated).ti,ab,kf. OR (Severely adj2 neurodevelopmentally incapacitated).ti,ab,kf. OR (Polyhandicap).ti,ab,kf. OR (Polyhandicaps).ti,ab,kf. OR (Trainable adj2 mentally retardate*).ti,ab,kf. OR (Complex adj2 **cognitive retardation**).ti,ab,kf. OR (Multiple adj2 cognitive retardation).ti,ab,kf. OR (Profound adj2 cognitive retardation).ti,ab,kf. OR (Serious adj2 cognitive retardation).ti,ab,kf. OR (Severe adj2 cognitive retardation).ti,ab,kf. OR (Complex adj2 **developmental retardation**).ti,ab,kf. OR (Multiple adj2 developmental retardation).ti,ab,kf. OR (Profound adj2 developmental retardation).ti,ab,kf. OR (Serious adj2 developmental retardation).ti,ab,kf. OR (Severe adj2 developmental retardation).ti,ab,kf. OR (Complex adj2 **intellectual retardation**).ti,ab,kf. OR (Multiple adj2 intellectual retardation).ti,ab,kf. OR (Profound adj2 intellectual retardation).ti,ab,kf. OR (Serious adj2 intellectual retardation).ti,ab,kf. OR (Severe adj2 intellectual retardation).ti,ab,kf. OR (Complex adj2 **mental retardation**).ti,ab,kf. OR (Multiple adj2 mental retardation).ti,ab,kf. OR (Profound adj2 mental retardation).ti,ab,kf. OR (Serious adj2 mental retardation).ti,ab,kf. OR (Severe adj2 mental retardation).ti,ab,kf. OR (Trainable adj2 mental retardation).ti,ab,kf. OR (Complex adj2 **neurodevelopmental retardation**).ti,ab,kf. OR (Multiple adj2 neurodevelopmental retardation).ti,ab,kf. OR (Profound adj2 neurodevelopmental retardation).ti,ab,kf. OR (Serious adj2 neurodevelopmental retardation).ti,ab,kf. OR (Severe adj2 neurodevelopmental retardation).ti,ab,kf. OR (Multiply adj2 **cognitively retarded**).ti,ab,kf. OR (Profoundly adj2 cognitively retarded).ti,ab,kf. OR (Seriously adj2 cognitively retarded).ti,ab,kf. OR (Severely adj2 cognitively retarded).ti,ab,kf. OR (Multiply adj2 **developmentally retarded**).ti,ab,kf. OR (Profoundly adj2 developmentally retarded).ti,ab,kf. OR (Seriously adj2 developmentally retarded).ti,ab,kf. OR (Severely adj2 developmentally retarded).ti,ab,kf. OR (**Multiply adj2 intellectually retarded**).ti,ab,kf. OR (Profoundly adj2 intellectually retarded).ti,ab,kf. OR (Seriously adj2 intellectually retarded).ti,ab,kf. OR (Severely adj2 intellectually retarded).ti,ab,kf. OR (Multiply adj2 **mentally retarded**).ti,ab,kf. OR (Profoundly adj2 mentally retarded).ti,ab,kf. OR (Seriously adj2 mentally retarded).ti,ab,kf. OR (Severely adj2 mentally retarded).ti,ab,kf. OR (Multiply adj2 **neurodevelopmentally retarded**).ti,ab,kf. OR (Profoundly adj2 neurodevelopmentally retarded).ti,ab,kf. OR (Seriously adj2 neurodevelopmentally retarded).ti,ab,kf. OR (Severely adj2 neurodevelopmentally retarded).ti,ab,kf. OR (Trainable adj2 mentally retarded).ti,ab,kf. OR (Trainable adj2 retarded).ti,ab,kf.)

AND

(exp Patient Acuity/ OR (patient adj2 acuity).ti,ab,kf. OR (Severity adj2 illness).ti,ab,kf. OR (illness adj2 severit*).ti,ab,kf. OR (disease adj2 severit*).ti,ab,kf. OR (sickness).ti,ab,kf. OR exp Patient-Reported Outcome/ OR (patient adj1 reported adj1 outcome*).ti,ab,kf.OR exp Pain/ OR pain*.ti,ab,kf. OR exp Comorbidity/ OR comorbidit*.ti,ab,kf. OR (medical adj2 condition*).ti,ab,kf. OR (clinical adj2 characteristic*).ti,ab,kf. OR (health adj2 status).ti,ab,kf. OR (physical adj2 condition*).ti,ab,kf. OR (chronic adj2 disease*).ti,ab,kf. OR (health adj2 problem*).ti,ab,kf. OR (health adj2 indicator*).ti,ab,kf. OR (health adj2 issue*).ti,ab,kf. OR (secondary adj2 condition*).ti,ab,kf. OR (physical adj2 health*).ti,ab,kf. OR exp Enteral nutrition/ OR (enteral adj2 nutrition).ti,ab,kf. OR (nutritional adj2 deficienc*).ti,ab,kf. OR (tube feed*).ti,ab,kf. OR (tube fed*).ti,ab,kf. OR (enteral feeding).ti,ab,kf. OR exp gastrostomy/ OR gastrostom*.ti,ab,kf. OR exp musculoskeletal disease/ OR (orthopedic adj2 disorder*).ti,ab,kf. OR (orthopaedic adj2 disorder*).ti,ab,kf. OR exp scoliosis/ OR (scoliosis).ti,ab,kf. OR exp Epilepsy/ OR (epilepsy).ti,ab,kf. OR (seizures).ti,ab,kf. OR exp osteoporosis/ OR osteoporos*.ti,ab,kf. OR exp Sleep Disorder/ OR (sleep adj2 problem*).ti,ab,kf. OR (sleep adj2 disorder*).ti,ab,kf. OR exp Vision Disorders/ OR (visual adj2 impairment).ti,ab,kf. OR exp Hearing Loss/ OR (hearing adj2 impairment).ti,ab,kf. OR (Hearing adj2 disorder*).ti,ab,kf. OR exp Dental Care/ OR (dental adj2 care).ti,ab,kf. OR (dental adj2 health).ti,ab,kf. OR (dental adj2 problem*).ti,ab,kf. OR exp Urinary Incontinence/ OR (urine adj2 incontinence).ti,ab,kf. OR exp Constipation/ OR (constipation).ti,ab,kf. OR exp Respiratory Tract Infections/ OR (respiratory adj2 infection*).ti,ab,kf. OR exp Body Size/ OR underweight.ti,ab,kf. OR overweight.ti,ab,kf. OR exp Pressure Ulcer/ OR decubitus.ti,ab,kf. OR (pressure adj2 ulcer).ti,ab,kf. OR exp Muscle Spasticity/ OR (muscle adj2 spasticit*).ti,ab,kf. OR (muscle adj2 spasm*).ti,ab,kf.OR exp gastroesophageal reflux/ OR (gastroesophageal adj2 reflux) .ti,ab,kf. OR dysphagia.ti,ab,kf. OR exp Sialorrhea/ OR Sialorrhea.ti,ab,kf. OR drooling.ti,ab,kf. OR exp Hypertension/ OR hypertension.ti,ab,kf. OR exp Endocrine System Diseases/ OR (endocrine adj2 disorder*).ti,ab,kf. OR exp Metabolic Syndrome/ OR (metabolic syndrome).ti,ab,kf. OR exp Menstrual Cycle/ OR (menstrual cycle).ti,ab,kf. OR exp Heart Defects, Congenital/ OR (congenital adj2 heart defect*).ti,ab,kf.)

**Medline Search**

((Complex adj2 **developmental abnormalit**y).ti,ab,kf. OR (Multiple adj2 developmental abnormality).ti,ab,kf. OR (Profound adj2 developmental abnormality).ti,ab,kf. OR (Serious adj2 developmental abnormality).ti,ab,kf. OR (Severe adj2 developmental abnormality).ti,ab,kf. OR (Complex adj2 **developmental abnormalit**ies).ti,ab,kf. OR (Multiple adj2 developmental abnormalities).ti,ab,kf. OR (Profound adj2 developmental abnormalities).ti,ab,kf. OR (Serious adj2 developmental abnormalities).ti,ab,kf. OR (Severe adj2 developmental abnormalities).ti,ab,kf.

OR (**Complex adj2** **intellectual abnormality**).ti,ab,kf. OR (Multiple adj2 intellectual abnormality).ti,ab,kf. OR (Profound adj2 intellectual abnormality).ti,ab,kf. OR (Serious adj2 intellectual abnormality).ti,ab,kf. OR (Severe adj2 intellectual abnormality).ti,ab,kf. OR (**Complex adj2 intellectual abnormalities**).ti,ab,kf. OR (Multiple adj2 intellectual abnormalities).ti,ab,kf. OR (Profound adj2 intellectual abnormalities).ti,ab,kf. OR (Serious adj2 intellectual abnormalities).ti,ab,kf. OR (Severe adj2 intellectual abnormalities).ti,ab,kf.

OR (Complex adj2 **mental abnormality**).ti,ab,kf. OR (Multiple adj2 mental abnormality).ti,ab,kf. OR (Profound adj2 mental abnormality).ti,ab,kf. OR (Serious adj2 mental abnormality).ti,ab,kf. OR (Severe adj2 mental abnormality).ti,ab,kf. OR (Complex adj2 **mental abnormalities**).ti,ab,kf. OR (Multiple adj2 mental abnormalities).ti,ab,kf. OR (Profound adj2 mental abnormalities).ti,ab,kf. OR (Serious adj2 mental abnormalities).ti,ab,kf. OR (Severe adj2 mental abnormalities).ti,ab,kf.

OR (Complex adj2 **neurodevelopmental abnormality**).ti,ab,kf. OR (Multiple adj2 neurodevelopmental abnormality).ti,ab,kf. OR (Profound adj2 neurodevelopmental abnormality).ti,ab,kf. OR (Serious adj2 neurodevelopmental abnormality).ti,ab,kf. OR (Severe adj2 neurodevelopmental abnormality).ti,ab,kf. OR (Complex adj2 **neurodevelopmental abnormalities**).ti,ab,kf. OR (Multiple adj2 neurodevelopmental abnormalities).ti,ab,kf. OR (Profound adj2 neurodevelopmental abnormalities).ti,ab,kf. OR (Serious adj2 neurodevelopmental abnormalities).ti,ab,kf. OR (Severe adj2 neurodevelopmental abnormalities).ti,ab,kf.

OR (Complex adj2 **cognitive defect**).ti,ab,kf. OR (Multiple adj2 cognitive defect).ti,ab,kf. OR (Profound adj2 cognitive defect).ti,ab,kf. OR (Serious adj2 cognitive defect).ti,ab,kf. OR (Severe adj2 cognitive defect).ti,ab,kf. OR (Complex adj2 **cognitive defect**s).ti,ab,kf. OR (Multiple adj2 cognitive defects).ti,ab,kf. OR (Profound adj2 cognitive defects).ti,ab,kf. OR (Serious adj2 cognitive defects).ti,ab,kf. OR (Severe adj2 cognitive defects).ti,ab,kf.

OR (Complex adj2 **developmental defect**).ti,ab,kf. OR (Multiple adj2 developmental defect).ti,ab,kf. OR (Profound adj2 developmental defect).ti,ab,kf. OR (Serious adj2 developmental defect).ti,ab,kf. OR (Severe adj2 developmental defect*).ti,ab,kf. OR (Complex adj2 **developmental defects**).ti,ab,kf. OR (Multiple adj2 developmental defects).ti,ab,kf. OR (Profound adj2 developmental defects).ti,ab,kf. OR (Serious adj2 developmental defects).ti,ab,kf. OR (Severe adj2 developmental defects).ti,ab,kf.

OR (Complex adj2 **intellectual defect**).ti,ab,kf. OR (Multiple adj2 intellectual defect).ti,ab,kf. OR (Profound adj2 intellectual defect).ti,ab,kf. OR (Serious adj2 intellectual defect).ti,ab,kf. OR (Severe adj2 intellectual defect).ti,ab,kf. OR (Complex adj2 **intellectual defects**).ti,ab,kf. OR (Multiple adj2 intellectual defects).ti,ab,kf. OR (Profound adj2 intellectual defects).ti,ab,kf. OR (Serious adj2 intellectual defects).ti,ab,kf. OR (Severe adj2 intellectual defects).ti,ab,kf.

OR (Complex adj2 **mental defect**).ti,ab,kf. OR (Multiple adj2 mental defect).ti,ab,kf. OR (Profound adj2 mental defect).ti,ab,kf. OR (Serious adj2 mental defect).ti,ab,kf. OR (Severe adj2 mental defect).ti,ab,kf. OR (Complex adj2 **mental defects**).ti,ab,kf. OR (Multiple adj2 mental defects).ti,ab,kf. OR (Profound adj2 mental defects).ti,ab,kf. OR (Serious adj2 mental defects).ti,ab,kf. OR (Severe adj2 mental defects).ti,ab,kf.

OR (Complex adj2 **neurodevelopmental defect**).ti,ab,kf. OR (Multiple adj2 neurodevelopmental defect).ti,ab,kf. OR (Profound adj2 neurodevelopmental defect).ti,ab,kf. OR (Serious adj2 neurodevelopmental defect).ti,ab,kf. OR (Severe adj2 neurodevelopmental defect).ti,ab,kf. OR (Complex adj2 **neurodevelopmental defect**s).ti,ab,kf. OR (Multiple adj2 neurodevelopmental defects).ti,ab,kf. OR (Profound adj2 neurodevelopmental defects).ti,ab,kf. OR (Serious adj2 neurodevelopmental defects).ti,ab,kf. OR (Severe adj2 neurodevelopmental defects).ti,ab,kf.

OR (Multiply adj2 **cognitively defective**).ti,ab,kf. OR (Profoundly adj2 cognitively defective).ti,ab,kf. OR (Seriously adj2 cognitively defective).ti,ab,kf. OR (Severely adj2 cognitively defective).ti,ab,kf.

OR (Multiply adj2 **developmentally defective**).ti,ab,kf. OR (Profoundly adj2 developmentally defective).ti,ab,kf. OR (Seriously adj2 developmentally defective).ti,ab,kf. OR (Severely adj2 developmentally defective).ti,ab,kf.

OR (Multiply adj2 **intellectually defective**).ti,ab,kf. OR (Profoundly adj2 intellectually defective).ti,ab,kf. OR (Seriously adj2 intellectually defective).ti,ab,kf. OR (Severely adj2 intellectually defective).ti,ab,kf.

OR (Profoundly adj2 **mentally defective**).ti,ab,kf. OR (Seriously adj2 mentally defective).ti,ab,kf. OR (Severely adj2 mentally defective).ti,ab,kf.

OR (Multiply adj2 **neurodevelopmentally defectiv**e).ti,ab,kf. OR (Profoundly adj2 neurodevelopmentally defective).ti,ab,kf. OR (Seriously adj2 neurodevelopmentally defective).ti,ab,kf. OR (Severely adj2 neurodevelopmentally defective).ti,ab,kf.

OR (Complex adj2 **cognitive deficienc***).ti,ab,kf. OR (Multiple adj2 cognitive deficienc*).ti,ab,kf. OR (Profound adj2 cognitive deficienc*).ti,ab,kf. OR (Serious adj2 cognitive deficienc*).ti,ab,kf. OR (Severe adj2 cognitive deficienc*).ti,ab,kf.

OR (Complex adj2 **developmental deficienc***).ti,ab,kf. OR (Multiple adj2 developmental deficienc*).ti,ab,kf. OR (Profound adj2 developmental deficienc*).ti,ab,kf. OR (Serious adj2 developmental deficienc*).ti,ab,kf. OR (Severe adj2 developmental deficienc*).ti,ab,kf.

OR (Complex adj2 **intellectual deficienc***).ti,ab,kf. OR (Multiple adj2 intellectual deficienc*).ti,ab,kf. OR (Profound adj2 intellectual deficienc*).ti,ab,kf. OR (Serious adj2 intellectual deficienc*).ti,ab,kf. OR (Severe adj2 intellectual deficienc*).ti,ab,kf.

OR (Complex adj2 **mental deficienc***).ti,ab,kf. OR (Multiple adj2 mental deficienc*).ti,ab,kf. OR (Profound adj2 mental deficienc*).ti,ab,kf. OR (Serious adj2 mental deficienc*).ti,ab,kf. OR (Severe adj2 mental deficienc*).ti,ab,kf.

OR (Complex adj2 **neurodevelopmental deficienc***).ti,ab,kf. OR (Multiple adj2 neurodevelopmental deficienc*).ti,ab,kf. OR (Profound adj2 neurodevelopmental deficienc*).ti,ab,kf. OR (Serious adj2 neurodevelopmental deficienc*).ti,ab,kf. OR (Severe adj2 neurodevelopmental deficienc*).ti,ab,kf.

OR (Multiply adj2 **cognitively deficient**).ti,ab,kf. OR (Profoundly adj2 cognitively deficient).ti,ab,kf. OR (Seriously adj2 cognitively deficient).ti,ab,kf. OR (Severely adj2 cognitively deficient).ti,ab,kf.

OR (Multiply adj2 **developmentally deficient).ti,ab,kf. OR (**Profoundly adj2 developmentally deficient).ti,ab,kf. OR (Seriously adj2 developmentally deficient).ti,ab,kf. OR (Severely adj2 developmentally deficient).ti,ab,kf.

OR (Multiply adj2 **intellectually deficient**).ti,ab,kf. OR (Profoundly adj2 intellectually deficient).ti,ab,kf. OR (Seriously adj2 intellectually deficient).ti,ab,kf. OR (Severely adj2 intellectually deficient).ti,ab,kf.

OR (Multiply adj2 **mentally deficient**).ti,ab,kf. OR (Profoundly adj2 mentally deficient).ti,ab,kf. OR (Seriously adj2 mentally deficient).ti,ab,kf. OR (Severely adj2 mentally deficient).ti,ab,kf.

OR (Multiply adj2 **neurodevelopmentally deficient**).ti,ab,kf. OR (Profoundly adj2 neurodevelopmentally deficient).ti,ab,kf. OR (Seriously adj2 neurodevelopmentally deficient).ti,ab,kf. OR (Severely adj2 neurodevelopmentally deficient).ti,ab,kf.

OR (**Complex adj2 developmental deficit***).ti,ab,kf. OR (Multiple adj2 developmental deficit*).ti,ab,kf. OR (Profound adj2 developmental deficit*).ti,ab,kf. OR (Serious adj2 developmental deficit*).ti,ab,kf. OR (Severe adj2 developmental deficit*).ti,ab,kf.

OR (Complex adj2 **intellectual deficit***).ti,ab,kf. OR (Multiple adj2 intellectual deficit*).ti,ab,kf. OR (Profound adj2 intellectual deficit*).ti,ab,kf. OR (Serious adj2 intellectual deficit*).ti,ab,kf. OR (Severe adj2 intellectual deficit*).ti,ab,kf.

OR (Complex adj2 **mental deficit*).ti,ab,kf. OR (**Multiple adj2 mental deficit*).ti,ab,kf. OR (Profound adj2 mental deficit*).ti,ab,kf. OR (Serious adj2 mental deficit*).ti,ab,kf. OR (Severe adj2 mental deficit*).ti,ab,kf.

OR (Complex adj2 **neurodevelopmental deficit***).ti,ab,kf. OR (Multiple adj2 neurodevelopmental deficit*).ti,ab,kf. OR (Profound adj2 neurodevelopmental deficit*).ti,ab,kf. OR (Serious adj2 neurodevelopmental deficit*).ti,ab,kf. OR (Severe adj2 neurodevelopmental deficit*).ti,ab,kf.

OR (Complex adj2 **cognitive delay**).ti,ab,kf. OR (Multiple adj2 cognitive delay).ti,ab,kf. OR (Profound adj2 cognitive delay).ti,ab,kf. OR (Serious adj2 cognitive delay).ti,ab,kf. OR (Severe adj2 cognitive delay).ti,ab,kf.

OR (Complex adj2 **developmental delay**).ti,ab,kf. OR (Multiple adj2 developmental delay).ti,ab,kf. OR (Profound adj2 developmental delay).ti,ab,kf. OR (Serious adj2 developmental delay).ti,ab,kf. OR (Severe adj2 developmental delay).ti,ab,kf.

OR (Complex adj2 **intellectual delay**).ti,ab,kf. OR (Multiple adj2 intellectual delay).ti,ab,kf. OR (Profound adj2 intellectual delay).ti,ab,kf. OR (Serious adj2 intellectual delay).ti,ab,kf. OR (Severe adj2 intellectual delay).ti,ab,kf.

OR (Complex adj2 **mental delay**).ti,ab,kf. OR (Multiple adj2 mental delay).ti,ab,kf. OR (Profound adj2 mental delay).ti,ab,kf. OR (Serious adj2 mental delay).ti,ab,kf. OR (Severe adj2 mental delay).ti,ab,kf.

OR (Complex adj2 **neurodevelopmental delay**).ti,ab,kf. OR (Multiple adj2 neurodevelopmental delay).ti,ab,kf. OR (Profound adj2 neurodevelopmental delay).ti,ab,kf. OR (Serious adj2 neurodevelopmental delay).ti,ab,kf. OR (Severe adj2 neurodevelopmental delay).ti,ab,kf.

OR (Multiply adj2 **cognitively delayed**).ti,ab,kf. OR (Profoundly adj2 cognitively delayed).ti,ab,kf. OR (Seriously adj2 cognitively delayed).ti,ab,kf. OR (Severely adj2 cognitively delayed).ti,ab,kf.

OR (Multiply adj2 **developmentally delayed**).ti,ab,kf. OR (Profoundly adj2 developmentally delayed).ti,ab,kf. OR (Seriously adj2 developmentally delayed).ti,ab,kf. OR (Severely adj2 developmentally delayed).ti,ab,kf.

OR (Multiply adj2 **intellectually delayed**).ti,ab,kf. OR (Profoundly adj2 intellectually delayed).ti,ab,kf. OR (Seriously adj2 intellectually delayed).ti,ab,kf. OR (Severely adj2 intellectually delayed).ti,ab,kf.

OR (Multiply adj2 **mentally delayed).ti,ab,kf. OR (**Profoundly adj2 mentally delayed).ti,ab,kf. OR (Seriously adj2 mentally delayed).ti,ab,kf. OR (Severely adj2 mentally delayed).ti,ab,kf.

OR (Multiply adj2 **neurodevelopmentally delayed).ti,ab,kf. OR (**Profoundly adj2 neurodevelopmentally delayed).ti,ab,kf. OR (Seriously adj2 neurodevelopmentally delayed).ti,ab,kf. OR (Severely adj2 neurodevelopmentally delayed).ti,ab,kf.

OR (Multiply adj2 **differently-abled**).ti,ab,kf. OR (Profoundly adj2 differently-abled).ti,ab,kf. OR (Seriously adj2 differently-abled).ti,ab,kf. OR (Severely adj2 differently-abled).ti,ab,kf.

OR (Multiple adj2 **developmental difficult**y).ti,ab,kf. OR (Complex adj2 developmental difficulty).ti,ab,kf. OR (Profound adj2 developmental difficulty).ti,ab,kf. OR (Serious adj2 developmental difficulty).ti,ab,kf. OR (Severe adj2 developmental difficulty).ti,ab,kf. OR (Multiple adj2 **developmental difficult**ies).ti,ab,kf. OR (Complex adj2 developmental difficulties).ti,ab,kf. OR (Profound adj2 developmental difficulties).ti,ab,kf. OR (Serious adj2 developmental difficulties).ti,ab,kf. OR (Severe adj2 developmental difficulties).ti,ab,kf.

OR (**Multiple adj2 intellectual difficulty**).ti,ab,kf. OR (Complex adj2 intellectual difficulty).ti,ab,kf. OR (Profound adj2 intellectual difficulty).ti,ab,kf. OR (Serious adj2 intellectual difficulty).ti,ab,kf. OR (Severe adj2 intellectual difficulty).ti,ab,kf. OR (**Multiple adj2 intellectual difficulties**).ti,ab,kf. OR (Complex adj2 intellectual difficulties).ti,ab,kf. OR (Profound adj2 intellectual difficulties).ti,ab,kf. OR (Serious adj2 intellectual difficulties).ti,ab,kf. OR (Severe adj2 intellectual difficulties).ti,ab,kf.

OR (Multiple adj2 **mental difficulty**).ti,ab,kf. OR (Complex adj2 mental difficulty).ti,ab,kf. OR (Profound adj2 mental difficulty).ti,ab,kf. OR (Serious adj2 mental difficulty).ti,ab,kf. OR (Severe adj2 mental difficulty).ti,ab,kf. OR (Multiple adj2 **mental difficulties**).ti,ab,kf. OR (Complex adj2 mental difficulties).ti,ab,kf. OR (Profound adj2 mental difficulties).ti,ab,kf. OR (Serious adj2 mental difficulties).ti,ab,kf. OR (Severe adj2 mental difficulties).ti,ab,kf.

OR (Complex adj2 **neurodevelopmental difficulty**).ti,ab,kf. OR (Multiple adj2 neurodevelopmental difficulty).ti,ab,kf. OR (Profound adj2 neurodevelopmental difficulty).ti,ab,kf. OR (Serious adj2 neurodevelopmental difficulty).ti,ab,kf. OR (Severe adj2 neurodevelopmental difficulty).ti,ab,kf. OR (Complex adj2 **neurodevelopmental difficulties**).ti,ab,kf. OR (Multiple adj2 neurodevelopmental difficulties).ti,ab,kf. OR (Profound adj2 neurodevelopmental difficulties).ti,ab,kf. OR (Serious adj2 neurodevelopmental difficulties).ti,ab,kf. OR (Severe adj2 neurodevelopmental difficulties).ti,ab,kf.

OR (Complex adj2 **cognitive disability**).ti,ab,kf. OR (Multiple adj2 cognitive disability).ti,ab,kf. OR (Profound adj2 cognitive disability).ti,ab,kf. OR (Serious adj2 cognitive disability).ti,ab,kf. OR (Severe adj2 cognitive disability).ti,ab,kf. OR (Complex adj2 **cognitive disabilities**).ti,ab,kf. OR (Multiple adj2 cognitive disabilities).ti,ab,kf. OR (Profound adj2 cognitive disabilities).ti,ab,kf. OR (Serious adj2 cognitive disabilities).ti,ab,kf. OR (Severe adj2 cognitive disabilities).ti,ab,kf.

OR (Complex adj2 **developmental disability**).ti,ab,kf. OR (Multiple adj2 developmental disability).ti,ab,kf. OR (Multiple adj2 developmental disability).ti,ab,kf. OR (Serious adj2 developmental disability).ti,ab,kf. OR (Severe adj2 developmental disability).ti,ab,kf. OR (Complex adj2 **developmental disabilities**).ti,ab,kf. OR (Multiple adj2 developmental disabilities).ti,ab,kf. OR (Multiple adj2 developmental disabilities).ti,ab,kf. OR (Serious adj2 developmental disabilities).ti,ab,kf. OR (Severe adj2 developmental disabilities).ti,ab,kf.

OR (Trainable adj2 **intellectual disabilit**y).ti,ab,kf. OR (Complex adj2 intellectual disability).ti,ab,kf. OR (Multiple adj2 intellectual disability).ti,ab,kf. OR (Profound adj2 intellectual disability).ti,ab,kf. OR (Serious adj2 intellectual disability).ti,ab,kf. OR (Severe adj2 intellectual disability).ti,ab,kf. OR (Trainable adj2 **intellectual disabilit**ies).ti,ab,kf. OR (Complex adj2 intellectual disabilities).ti,ab,kf. OR (Multiple adj2 intellectual disabilities).ti,ab,kf. OR (Profound adj2 intellectual disabilities).ti,ab,kf. OR (Serious adj2 intellectual disabilities).ti,ab,kf. OR (Severe adj2 intellectual disabilities).ti,ab,kf.

OR (**Severe adj2 Profound adj2 intellectual motor disability**).ti,ab,kf. OR (**Severe adj2 Profound adj2 intellectual motor disabilities)**.ti,ab,kf.

OR (Complex adj2 **mental disabilit**y).ti,ab,kf. OR (Multiple adj2 mental disability).ti,ab,kf. OR (Profound adj2 mental disability).ti,ab,kf. OR (Serious adj2 mental disability).ti,ab,kf. OR (Severe adj2 mental disability).ti,ab,kf. OR (Trainable adj2 mental disability).ti,ab,kf. OR (Complex adj2 **mental disabilit**ies).ti,ab,kf. OR (Multiple adj2 mental disabilities).ti,ab,kf. OR (Profound adj2 mental disabilities).ti,ab,kf. OR (Serious adj2 mental disabilities).ti,ab,kf. OR (Severe adj2 mental disabilities).ti,ab,kf. OR (Trainable adj2 mental disabilities).ti,ab,kf.

OR (Complex adj2 **neurodevelopmental disabilit**y).ti,ab,kf. OR (Multiple adj2 neurodevelopmental disability).ti,ab,kf. OR (Profound adj2 neurodevelopmental disability).ti,ab,kf. OR (Serious adj2 neurodevelopmental disability).ti,ab,kf. OR (Severe adj2 neurodevelopmental disability).ti,ab,kf. OR (Complex adj2 **neurodevelopmental disabilit**ies).ti,ab,kf. OR (Multiple adj2 neurodevelopmental disabilities).ti,ab,kf. OR (Profound adj2 neurodevelopmental disabilities).ti,ab,kf. OR (Serious adj2 neurodevelopmental disabilities).ti,ab,kf. OR (Severe adj2 neurodevelopmental disabilities).ti,ab,kf.

OR (Multiple adj2 disability).ti,ab,kf. OR (Multiple adj2 disabilities).ti,ab,kf.

OR (Multiply adj2 **cognitively disabled**).ti,ab,kf. OR (Profoundly adj2 cognitively disabled).ti,ab,kf. OR (Seriously adj2 cognitively disabled).ti,ab,kf. OR (Severely adj2 cognitively disabled).ti,ab,kf.

OR (Multiply adj2 **developmentally disabled**).ti,ab,kf. OR (Profoundly adj2 developmentally disabled).ti,ab,kf. OR (Seriously adj2 developmentally disabled).ti,ab,kf. OR (Severely adj2 developmentally disabled).ti,ab,kf. OR (Trainable adj2 developmentally disabled).ti,ab,kf.

OR (Multiply adj2 **intellectually disabled**).ti,ab,kf. OR (Profoundly adj2 intellectually disabled).ti,ab,kf. OR (Seriously adj2 intellectually disabled).ti,ab,kf. OR (Severely adj2 intellectually disabled).ti,ab,kf. OR (Trainable adj2 intellectually disabled).ti,ab,kf.

OR (Multiply adj2 **mentally disabled**).ti,ab,kf. OR (Profoundly adj2 mentally disabled).ti,ab,kf. OR (Seriously adj2 mentally disabled).ti,ab,kf. OR (Severely adj2 mentally disabled).ti,ab,kf.

OR (Multiply adj2 **neurodevelopmentally disabled**).ti,ab,kf. OR (Profoundly adj2 neurodevelopmentally disabled).ti,ab,kf. OR (Seriously adj2 neurodevelopmentally disabled).ti,ab,kf. OR (Severely adj2 neurodevelopmentally disabled).ti,ab,kf. OR (Multiply adj2 disabled).ti,ab,kf. OR (Trainable adj2 **mentally disabled)**.ti,ab,kf.

OR (Complex adj2 **developmental disorder***).ti,ab,kf. OR (Multiple adj2 developmental disorder*).ti,ab,kf. OR (Profound adj2 developmental disorder*).ti,ab,kf. OR (Serious adj2 developmental disorder*).ti,ab,kf. OR (Severe adj2 developmental disorder*).ti,ab,kf.

OR (Complex adj2 **intellectual disorder**).ti,ab,kf. OR (Multiple adj2 intellectual disorder).ti,ab,kf. OR (Profound adj2 intellectual disorder).ti,ab,kf. OR (Serious adj2 intellectual disorder).ti,ab,kf. OR (Severe adj2 intellectual disorder).ti,ab,kf. OR (Complex adj2 **intellectual disorders**).ti,ab,kf. OR (Multiple adj2 intellectual disorders).ti,ab,kf. OR (Profound adj2 intellectual disorders).ti,ab,kf. OR (Serious adj2 intellectual disorders).ti,ab,kf. OR (Severe adj2 intellectual disorders).ti,ab,kf.

OR (Complex adj2 **neurodevelopmental disorder**).ti,ab,kf. OR (Multiple adj2 neurodevelopmental disorder).ti,ab,kf. OR (Profound adj2 neurodevelopmental disorder).ti,ab,kf. OR (Serious adj2 neurodevelopmental disorder).ti,ab,kf. OR (Severe adj2 neurodevelopmental disorder).ti,ab,kf. OR (Complex adj2 **neurodevelopmental disorder**s).ti,ab,kf. OR (Multiple adj2 neurodevelopmental disorders).ti,ab,kf. OR (Profound adj2 neurodevelopmental disorders).ti,ab,kf. OR (Serious adj2 neurodevelopmental disorders).ti,ab,kf. OR (Severe adj2 neurodevelopmental disorders).ti,ab,kf.

OR (Complex adj2 **cognitive handicap***).ti,ab,kf. OR (Multiple adj2 cognitive handicap*).ti,ab,kf. OR (Profound adj2 cognitive handicap*).ti,ab,kf. OR (Serious adj2 cognitive handicap*).ti,ab,kf. OR (Severe adj2 cognitive handicap*).ti,ab,kf.

OR (Complex adj2 **developmental handicap***).ti,ab,kf. OR (Multiple adj2 developmental handicap*).ti,ab,kf. OR (Profound adj2 developmental handicap*).ti,ab,kf. OR (Serious adj2 developmental handicap*).ti,ab,kf. OR (Severe adj2 developmental handicap*).ti,ab,kf.

OR (Complex adj2 **intellectual handicap***).ti,ab,kf. OR (Multiple adj2 intellectual handicap*).ti,ab,kf. OR (Profound adj2 intellectual handicap*).ti,ab,kf. OR (Serious adj2 intellectual handicap*).ti,ab,kf. OR (Severe adj2 intellectual handicap*).ti,ab,kf.

OR (Complex adj2 **mental handicap***).ti,ab,kf. OR (Multiple adj2 mental handicap*).ti,ab,kf. OR (Profound adj2 mental handicap*).ti,ab,kf. OR (Serious adj2 mental handicap*).ti,ab,kf. OR (Severe adj2 mental handicap*).ti,ab,kf. OR (Trainable adj2 mental handicap*).ti,ab,kf.

OR (Complex adj2 **neurodevelopmental handicap***).ti,ab,kf. OR (Multiple adj2 neurodevelopmental handicap*).ti,ab,kf. OR (Profound adj2 neurodevelopmental handicap*).ti,ab,kf. OR (Serious adj2 neurodevelopmental handicap*).ti,ab,kf. OR (Severe adj2 neurodevelopmental handicap*).ti,ab,kf.

OR (Multiply adj2 **cognitively handicapped**).ti,ab,kf. OR (Profoundly adj2 cognitively handicapped).ti,ab,kf. OR (Seriously adj2 cognitively handicapped).ti,ab,kf. OR (Severely adj2 cognitively handicapped).ti,ab,kf.

OR (Multiply adj2 **developmentally handicapped**).ti,ab,kf. OR (Profoundly adj2 developmentally handicapped).ti,ab,kf. OR (Seriously adj2 developmentally handicapped).ti,ab,kf. OR (Severely adj2 developmentally handicapped).ti,ab,kf.

OR (**Multiply adj2 intellectually handicapped**).ti,ab,kf. OR (Profoundly adj2 intellectually handicapped).ti,ab,kf. OR (Seriously adj2 intellectually handicapped).ti,ab,kf. OR (Severely adj2 intellectually handicapped).ti,ab,kf.

OR (Multiply adj2 mentally handicapped).ti,ab,kf. OR (Profoundly adj2 **mentally handicapped**).ti,ab,kf. OR (Seriously adj2 mentally handicapped).ti,ab,kf. OR (Severely adj2 mentally handicapped).ti,ab,kf.

OR (Multiply adj2 **neurodevelopmentally handicapped**).ti,ab,kf. OR (Profoundly adj2 neurodevelopmentally handicapped).ti,ab,kf. OR (Seriously adj2 neurodevelopmentally handicapped).ti,ab,kf. OR (Severely adj2 neurodevelopmentally handicapped).ti,ab,kf. OR (Multiply adj2 handicapped).ti,ab,kf. OR (Trainable adj2 mentally handicapped).ti,ab,kf.

OR (Multiply adj2 **developmentally impaired**).ti,ab,kf. OR (Profoundly adj2 developmentally impaired).ti,ab,kf. OR (Seriously adj2 developmentally impaired).ti,ab,kf. OR (Severely adj2 developmentally impaired).ti,ab,kf.

OR (Multiply adj2 **intellectually impaired**).ti,ab,kf. OR (Profoundly adj2 intellectually impaired).ti,ab,kf. OR (Seriously adj2 intellectually impaired).ti,ab,kf. OR (Severely adj2 intellectually impaired).ti,ab,kf.

OR (Profoundly adj2 **mentally impaired**).ti,ab,kf. OR (Seriously adj2 mentally impaired).ti,ab,kf. OR (Severely adj2 mentally impaired).ti,ab,kf.

OR (Multiply adj2 **neurodevelopmentally impaired**).ti,ab,kf. OR (Profoundly adj2 neurodevelopmentally impaired).ti,ab,kf. OR (Seriously adj2 neurodevelopmentally impaired).ti,ab,kf. OR (Severely adj2 neurodevelopmentally impaired).ti,ab,kf. OR (Trainable adj2 mentally impaired).ti,ab,kf. OR (Complex adj2 developmental impairment*).ti,ab,kf.

OR (Multiple adj2 **developmental impairment***).ti,ab,kf. OR (Profound adj2 developmental impairment*).ti,ab,kf. OR (Serious adj2 developmental impairment*).ti,ab,kf. OR (Severe adj2 developmental impairment*).ti,ab,kf.

OR (Complex adj2 **intellectual impairment***).ti,ab,kf. OR (Multiple adj2 intellectual impairment*).ti,ab,kf. OR (Profound adj2 intellectual impairment*).ti,ab,kf. OR (Serious adj2 intellectual impairment*).ti,ab,kf. OR (Severe adj2 intellectual impairment*).ti,ab,kf.

OR (Complex adj2 **mental impairment***).ti,ab,kf. OR (Multiple adj2 mental impairment*).ti,ab,kf. OR (Profound adj2 mental impairment*).ti,ab,kf. OR (Serious adj2 mental impairment*).ti,ab,kf. OR (Severe adj2 mental impairment*).ti,ab,kf.

OR (Complex adj2 **neurodevelopmental impairment***).ti,ab,kf. OR (Multiple adj2 neurodevelopmental impairment*).ti,ab,kf. OR (Profound adj2 neurodevelopmental impairment*).ti,ab,kf. OR (Serious adj2 neurodevelopmental impairment*).ti,ab,kf. OR (Severe adj2 neurodevelopmental impairment*).ti,ab,kf. OR (Trainable adj2 mental impairment*).ti,ab,kf.

OR (Complex adj2 **cognitive incapacit***).ti,ab,kf. OR (Multiple adj2 cognitive incapacit*).ti,ab,kf. OR (Profound adj2 cognitive incapacit*).ti,ab,kf. OR (Serious adj2 cognitive incapacit*).ti,ab,kf. OR (Severe adj2 cognitive incapacit*).ti,ab,kf.

OR (Complex adj2 **developmental incapacit***).ti,ab,kf. OR (Multiple adj2 developmental incapacit*).ti,ab,kf. OR (Profound adj2 developmental incapacit*).ti,ab,kf. OR (Serious adj2 developmental incapacit*).ti,ab,kf. OR (Severe adj2 developmental incapacit*).ti,ab,kf.

OR (**Complex adj2 intellectual incapacit***).ti,ab,kf. OR (Multiple adj2 intellectual incapacit*).ti,ab,kf. OR (Profound adj2 intellectual incapacit*).ti,ab,kf. OR (Serious adj2 intellectual incapacit*).ti,ab,kf. OR (Severe adj2 intellectual incapacit*).ti,ab,kf.

OR (Complex adj2 **mental incapacit***).ti,ab,kf. OR (Multiple adj2 mental incapacit*).ti,ab,kf. OR (Profound adj2 mental incapacit*).ti,ab,kf. OR (Serious adj2 mental incapacit*).ti,ab,kf. OR (Severe adj2 mental incapacit*).ti,ab,kf.

OR (Complex adj2 **neurodevelopmental incapacit***).ti,ab,kf. OR (Multiple adj2 neurodevelopmental incapacit*).ti,ab,kf. OR (Profound adj2 neurodevelopmental incapacit*).ti,ab,kf. OR (Serious adj2 neurodevelopmental incapacit*).ti,ab,kf. OR (Severe adj2 neurodevelopmental incapacit*).ti,ab,kf.

OR (Multiply adj2 **cognitively incapacitated**).ti,ab,kf. OR (Profoundly adj2 cognitively incapacitated).ti,ab,kf. OR (Seriously adj2 cognitively incapacitated).ti,ab,kf. OR (Severely adj2 cognitively incapacitated).ti,ab,kf.

OR (Multiply adj2 **developmentally incapacitated**).ti,ab,kf. OR (Profoundly adj2 developmentally incapacitated).ti,ab,kf. OR (Seriously adj2 developmentally incapacitated).ti,ab,kf. OR (Severely adj2 developmentally incapacitated).ti,ab,kf.

OR (Multiply adj2 **intellectually incapacitated**).ti,ab,kf. OR (Profoundly adj2 intellectually incapacitated).ti,ab,kf. OR (Seriously adj2 intellectually incapacitated).ti,ab,kf. OR (Severely adj2 intellectually incapacitated).ti,ab,kf.

OR (Multiply adj2 **mentally incapacitated**).ti,ab,kf. OR (Profoundly adj2 mentally incapacitated).ti,ab,kf. OR (Seriously adj2 mentally incapacitated).ti,ab,kf. OR (Severely adj2 mentally incapacitated).ti,ab,kf.

OR (Multiply adj2 **neurodevelopmentally incapacitated**).ti,ab,kf. OR (Profoundly adj2 neurodevelopmentally incapacitated).ti,ab,kf. OR (Seriously adj2 neurodevelopmentally incapacitated).ti,ab,kf. OR (Severely adj2 neurodevelopmentally incapacitated).ti,ab,kf.

OR (Polyhandicap).ti,ab,kf. OR (Polyhandicaps).ti,ab,kf. OR (Trainable adj2 mentally retardate*).ti,ab,kf.

OR (Complex adj2 **cognitive retardation**).ti,ab,kf. OR (Multiple adj2 cognitive retardation).ti,ab,kf. OR (Profound adj2 cognitive retardation).ti,ab,kf. OR (Serious adj2 cognitive retardation).ti,ab,kf. OR (Severe adj2 cognitive retardation).ti,ab,kf.

OR (Complex adj2 **developmental retardation**).ti,ab,kf. OR (Multiple adj2 developmental retardation).ti,ab,kf. OR (Profound adj2 developmental retardation).ti,ab,kf. OR (Serious adj2 developmental retardation).ti,ab,kf. OR (Severe adj2 developmental retardation).ti,ab,kf.

OR (Complex adj2 **intellectual retardation**).ti,ab,kf. OR (Multiple adj2 intellectual retardation).ti,ab,kf. OR (Profound adj2 intellectual retardation).ti,ab,kf. OR (Serious adj2 intellectual retardation).ti,ab,kf. OR (Severe adj2 intellectual retardation).ti,ab,kf.

OR (Complex adj2 **mental retardation**).ti,ab,kf. OR (Multiple adj2 mental retardation).ti,ab,kf. OR (Profound adj2 mental retardation).ti,ab,kf. OR (Serious adj2 mental retardation).ti,ab,kf. OR (Severe adj2 mental retardation).ti,ab,kf. OR (Trainable adj2 mental retardation).ti,ab,kf.

OR (Complex adj2 **neurodevelopmental retardation**).ti,ab,kf. OR (Multiple adj2 neurodevelopmental retardation).ti,ab,kf. OR (Profound adj2 neurodevelopmental retardation).ti,ab,kf. OR (Serious adj2 neurodevelopmental retardation).ti,ab,kf. OR (Severe adj2 neurodevelopmental retardation).ti,ab,kf.

OR (Multiply adj2 **cognitively retarded**).ti,ab,kf. OR (Profoundly adj2 cognitively retarded).ti,ab,kf. OR (Seriously adj2 cognitively retarded).ti,ab,kf. OR (Severely adj2 cognitively retarded).ti,ab,kf.

OR (Multiply adj2 **developmentally retarded**).ti,ab,kf. OR (Profoundly adj2 developmentally retarded).ti,ab,kf. OR (Seriously adj2 developmentally retarded).ti,ab,kf. OR (Severely adj2 developmentally retarded).ti,ab,kf.

OR (**Multiply adj2 intellectually retarded**).ti,ab,kf. OR (Profoundly adj2 intellectually retarded).ti,ab,kf. OR (Seriously adj2 intellectually retarded).ti,ab,kf. OR (Severely adj2 intellectually retarded).ti,ab,kf.

OR (Multiply adj2 **mentally retarded**).ti,ab,kf. OR (Profoundly adj2 mentally retarded).ti,ab,kf. OR (Seriously adj2 mentally retarded).ti,ab,kf. OR (Severely adj2 mentally retarded).ti,ab,kf.

OR (Multiply adj2 **neurodevelopmentally retarded**).ti,ab,kf. OR (Profoundly adj2 neurodevelopmentally retarded).ti,ab,kf. OR (Seriously adj2 neurodevelopmentally retarded).ti,ab,kf. OR (Severely adj2 neurodevelopmentally retarded).ti,ab,kf. OR (Trainable adj2 mentally retarded).ti,ab,kf. OR (Trainable adj2 retarded).ti,ab,kf.)

AND

(exp Patient Acuity/ OR (patient adj2 acuity).ti,ab,kf. OR (Severity adj2 illness).ti,ab,kf. OR (illness adj2 severit*).ti,ab,kf. OR (disease adj2 severit*).ti,ab,kf. OR (sickness).ti,ab,kf. OR exp Patient Reported Outcome Measures/ OR (patient adj1 reported adj1 outcome*).ti,ab,kf.

OR exp Pain/ OR pain*.ti,ab,kf. OR exp Comorbidity/ OR comorbidit*.ti,ab,kf. OR (medical adj2 condition*).ti,ab,kf. OR (clinical adj2 characteristic*).ti,ab,kf. OR (health adj2 status).ti,ab,kf. OR (physical adj2 condition*).ti,ab,kf. OR (chronic adj2 disease*).ti,ab,kf. OR (health adj2 problem*).ti,ab,kf. OR (health adj2 indicator*).ti,ab,kf. OR (health adj2 issue*).ti,ab,kf. OR (secondary adj2 condition*).ti,ab,kf. OR (physical adj2 health*).ti,ab,kf.

OR exp Enteral nutrition/ OR (enteral adj2 nutrition).ti,ab,kf. OR (nutritional adj2 deficienc*).ti,ab,kf. OR (tube feed*).ti,ab,kf. OR (tube fed*).ti,ab,kf. OR (enteral feeding).ti,ab,kf. OR exp gastrostomy/ OR gastrostom*.ti,ab,kf.

OR exp musculoskeletal diseases/ OR (orthopedic adj2 disorder*).ti,ab,kf. OR (orthopaedic adj2 disorder*).ti,ab,kf. OR exp scoliosis/ OR (scoliosis).ti,ab,kf.

OR exp Epilepsy/ OR (epilepsy).ti,ab,kf. OR (seizures).ti,ab,kf. OR exp osteoporosis/ OR osteoporos*.ti,ab,kf. OR exp dyssomnias/ OR (sleep adj2 problem*).ti,ab,kf. OR (sleep adj2 disorder*).ti,ab,kf. OR exp Vision Disorders/ OR (visual adj2 impairment).ti,ab,kf. OR exp Hearing Loss/ OR (hearing adj2 impairment).ti,ab,kf. OR (Hearing adj2 disorder*).ti,ab,kf. OR exp Dental Care/ OR (dental adj2 care).ti,ab,kf. OR (dental adj2 health).ti,ab,kf. OR (dental adj2 problem*).ti,ab,kf. OR exp Urinary Incontinence/ OR (urine adj2 incontinence).ti,ab,kf. OR exp Constipation/ OR (constipation).ti,ab,kf.

OR exp Respiratory Tract Infections/ OR (respiratory adj2 infection*).ti,ab,kf. OR exp Body Size/ OR underweight.ti,ab,kf. OR overweight.ti,ab,kf. OR exp Pressure Ulcer/ OR decubitus.ti,ab,kf. OR (pressure adj2 ulcer).ti,ab,kf. OR exp Muscle Spasticity/ OR (muscle adj2 spasticit*).ti,ab,kf. OR (muscle adj2 spasm*).ti,ab,kf.

OR exp gastroesophageal reflux/ OR (gastroesophageal adj2 reflux) .ti,ab,kf. OR dysphagia.ti,ab,kf. OR exp Sialorrhea/ OR Sialorrhea.ti,ab,kf. OR drooling.ti,ab,kf. OR exp Hypertension/ OR hypertension.ti,ab,kf.

OR exp Endocrine System Diseases/ OR (endocrine adj2 disorder*).ti,ab,kf. OR exp Metabolic Syndrome/ OR (metabolic syndrome).ti,ab,kf. OR exp Menstrual Cycle/ OR (menstrual cycle).ti,ab,kf. OR exp Heart Defects, Congenital/ OR (congenital adj2 heart defect*).ti,ab,kf.)

**Pubmed Search**

(Complex **developmental abnormalit**y [tiab] OR Multiple developmental abnormality [tiab] OR Profound developmental abnormality [tiab] OR Serious developmental abnormality [tiab] OR Severe developmental abnormality [tiab] OR Complex **developmental abnormalit**ies [tiab] OR Multiple developmental abnormalities [tiab] OR Profound developmental abnormalities [tiab] OR Serious developmental abnormalities [tiab] OR severe developmental abnormalities [tiab]

OR **Complex intellectual abnormality** [tiab] OR Multiple intellectual abnormality [tiab] OR Profound intellectual abnormality [tiab] OR Serious intellectual abnormality [tiab] OR Severe intellectual abnormality [tiab] OR **Complex intellectual abnormalities** [tiab] OR Multiple intellectual abnormalities [tiab] OR Profound intellectual abnormalities [tiab] OR Serious intellectual abnormalities [tiab] OR Severe intellectual abnormalities [tiab]

OR Complex **mental abnormality** [tiab] OR Multiple mental abnormality [tiab] OR Profound mental abnormality [tiab] OR Serious mental abnormality [tiab] OR Severe mental abnormality [tiab] OR Complex **mental abnormalities** [tiab] OR Multiple mental abnormalities [tiab] OR Profound mental abnormalities [tiab] OR Serious mental abnormalities [tiab] OR Severe mental abnormalities [tiab]

OR Complex **neurodevelopmental abnormality** [tiab] OR Multiple neurodevelopmental abnormality [tiab] OR Profound neurodevelopmental abnormality [tiab] OR Serious neurodevelopmental abnormality [tiab] OR Severe neurodevelopmental abnormality [tiab] OR Complex **neurodevelopmental abnormalities** [tiab] OR Multiple neurodevelopmental abnormalities [tiab] OR Profound neurodevelopmental abnormalities [tiab] OR Serious neurodevelopmental abnormalities[tiab] OR Severe neurodevelopmental abnormalities [tiab]

OR Complex **cognitive defect** [tiab] OR Multiple cognitive defect [tiab] OR Profound cognitive defect [tiab] OR Serious cognitive defect [tiab] OR Severe cognitive defect [tiab] OR Complex **cognitive defect**s [tiab] OR Multiple cognitive defects [tiab] OR Profound cognitive defects [tiab] OR Serious cognitive defects [tiab] OR Severe cognitive defects [tiab]

OR Complex **developmental defect** [tiab] OR Multiple developmental defect [tiab] OR Profound developmental defect [tiab] OR Serious developmental defect [tiab] OR Severe developmental defect* [tiab] OR Complex **developmental defects** [tiab] OR Multiple developmental defects [tiab] OR Profound developmental defects [tiab] OR Serious developmental defects [tiab] OR Severe developmental defects [tiab]

OR Complex **intellectual defect** [tiab] OR Multiple intellectual defect [tiab] OR Profound intellectual defect [tiab] OR Serious intellectual defect [tiab] OR Severe intellectual defect [tiab] OR Complex **intellectual defects** [tiab] OR Multiple intellectual defects [tiab] OR Profound intellectual defects [tiab] OR Serious intellectual defects [tiab] OR Severe intellectual defects [tiab]

OR Complex **mental defect** [tiab] OR Multiple mental defect [tiab] OR Profound mental defect [tiab] OR Serious mental defect [tiab] OR Severe mental defect [tiab] OR Complex **mental defects** [tiab] OR Multiple mental defects [tiab] OR Profound mental defects [tiab] OR Serious mental defects [tiab] OR Severe mental defects [tiab]

OR Complex **neurodevelopmental defect** [tiab] OR Multiple neurodevelopmental defect [tiab] OR Profound neurodevelopmental defect [tiab] OR Serious neurodevelopmental defect [tiab] OR Severe neurodevelopmental defect [tiab] OR Complex **neurodevelopmental defect**s [tiab] OR Multiple neurodevelopmental defects [tiab] OR Profound neurodevelopmental defects [tiab] OR Serious neurodevelopmental defects [tiab] OR Severe neurodevelopmental defects [tiab]

OR Multiply **cognitively defective** [tiab] OR Profoundly cognitively defective [tiab] OR Seriously cognitively defective [tiab] OR Severely cognitively defective [tiab]

OR Multiply **developmentally defective** [tiab] OR Profoundly developmentally defective [tiab] OR Seriously developmentally defective [tiab] OR Severely developmentally defective [tiab]

OR Multiply **intellectually defective** [tiab] OR Profoundly intellectually defective [tiab] OR Seriously intellectually defective [tiab] OR Severely intellectually defective [tiab]

OR Profoundly **mentally defective** [tiab] OR Seriously mentally defective [tiab] OR Severely mentally defective [tiab]

OR Multiply **neurodevelopmentally defectiv**e [tiab] OR Profoundly neurodevelopmentally defective [tiab] OR Seriously neurodevelopmentally defective [tiab] OR Severely neurodevelopmentally defective [tiab]

OR Complex **cognitive deficienc*** [tiab] OR Multiple cognitive deficienc* [tiab] OR Profound cognitive deficienc* [tiab] OR Serious cognitive deficienc* [tiab] OR Severe cognitive deficienc* [tiab]

OR Complex **developmental deficienc*** [tiab] OR Multiple developmental deficienc* [tiab] OR Profound developmental deficienc* [tiab] OR Serious developmental deficienc* [tiab] OR Severe developmental deficienc* [tiab]

OR Complex **intellectual deficienc*** [tiab] OR Multiple intellectual deficienc* [tiab] OR Profound intellectual deficienc* [tiab] OR Serious intellectual deficienc* [tiab] OR Severe intellectual deficienc* [tiab]

OR Complex **mental deficienc*** [tiab] OR Multiple mental deficienc* [tiab] OR Profound mental deficienc* [tiab] OR Serious mental deficienc* [tiab] OR Severe mental deficienc* [tiab]

OR Complex **neurodevelopmental deficienc*** [tiab] OR Multiple neurodevelopmental deficienc* [tiab] OR Profound neurodevelopmental deficienc* [tiab] OR Serious neurodevelopmental deficienc* [tiab] OR Severe neurodevelopmental deficienc* [tiab]

OR Multiply **cognitively deficient** [tiab] OR Profoundly cognitively deficient [tiab] OR Seriously cognitively deficient [tiab] OR Severely cognitively deficient [tiab]

OR Multiply **developmentally deficient** [tiab] OR Profoundly developmentally deficient [tiab] OR Seriously developmentally deficient [tiab] OR Severely developmentally deficient [tiab]

OR Multiply **intellectually deficient** [tiab] OR Profoundly intellectually deficient [tiab] OR Seriously intellectually deficient [tiab] OR Severely intellectually deficient [tiab]

OR Multiply **mentally deficient** [tiab] OR Profoundly mentally deficient [tiab] OR Seriously mentally deficient [tiab] OR Severely mentally deficient [tiab]

OR Multiply **neurodevelopmentally deficient** [tiab] OR Profoundly neurodevelopmentally deficient [tiab] OR Seriously neurodevelopmentally deficient [tiab] OR Severely neurodevelopmentally deficient [tiab]

OR **Complex developmental deficit*** [tiab] OR Multiple developmental deficit* [tiab] OR Profound developmental deficit* [tiab] OR Serious developmental deficit* [tiab] OR Severe developmental deficit* [tiab]

OR Complex **intellectual deficit*** [tiab] OR Multiple intellectual deficit* [tiab] OR Profound intellectual deficit* [tiab] OR Serious intellectual deficit* [tiab] OR Severe intellectual deficit* [tiab]

OR Complex **mental deficit*** [tiab] OR Multiple mental deficit* [tiab] OR Profound mental deficit* [tiab] OR Serious mental deficit* [tiab] OR Severe mental deficit* [tiab]

OR Complex **neurodevelopmental deficit*** [tiab] OR Multiple neurodevelopmental deficit* [tiab] OR Profound neurodevelopmental deficit* [tiab] OR Serious neurodevelopmental deficit* [tiab] OR Severe neurodevelopmental deficit* [tiab]

OR Complex **cognitive delay** [tiab] OR Multiple cognitive delay [tiab] OR Profound cognitive delay [tiab] OR Serious cognitive delay [tiab] OR Severe cognitive delay [tiab]

OR Complex **developmental delay** [tiab] OR Multiple developmental delay [tiab] OR Profound developmental delay [tiab] OR Serious developmental delay [tiab] OR Severe developmental delay [tiab]

OR Complex **intellectual delay** [tiab] OR Multiple intellectual delay [tiab] OR Profound intellectual delay [tiab] OR Serious intellectual delay [tiab] OR Severe intellectual delay [tiab]

OR Complex **mental delay** [tiab] OR Multiple mental delay [tiab] OR Profound mental delay [tiab] OR Serious mental delay [tiab] OR Severe mental delay [tiab]

OR Complex **neurodevelopmental delay** [tiab] OR Multiple neurodevelopmental delay [tiab] OR Profound neurodevelopmental delay [tiab] OR Serious neurodevelopmental delay [tiab] OR Severe neurodevelopmental delay [tiab]

OR Multiply **cognitively delayed** [tiab] OR Profoundly cognitively delayed [tiab] OR Seriously cognitively delayed [tiab] OR Severely cognitively delayed [tiab]

OR Multiply **developmentally delayed** [tiab] OR Profoundly developmentally delayed [tiab] OR Seriously developmentally delayed [tiab] OR Severely developmentally delayed [tiab]

OR Multiply **intellectually delayed** [tiab] OR Profoundly intellectually delayed [tiab] OR Seriously intellectually delayed [tiab] OR Severely intellectually delayed [tiab]

OR Multiply **mentally delayed [**tiab] OR Profoundly mentally delayed [tiab] OR Seriously mentally delayed [tiab] OR Severely mentally delayed [tiab]

OR Multiply **neurodevelopmentally delayed** [tiab] OR Profoundly neurodevelopmentally delayed [tiab] OR Seriously neurodevelopmentally delayed [tiab] OR Severely neurodevelopmentally delayed [tiab]

OR Multiply **differently-abled** [tiab] OR Profoundly differently-abled [tiab] OR Seriously differently-abled [tiab] OR Severely differently-abled [tiab]

OR Multiple **developmental difficult**y [tiab] OR Complex developmental difficulty [tiab] OR Profound developmental difficulty [tiab] OR Serious developmental difficulty [tiab] OR Severe developmental difficulty [tiab] OR Multiple **developmental difficult**ies [tiab] OR Complex developmental difficulties [tiab] OR Profound developmental difficulties [tiab] OR Serious developmental difficulties [tiab] OR Severe developmental difficulties [tiab]

OR **Multiple intellectual difficulty** [tiab] OR Complex intellectual difficulty [tiab] OR Profound intellectual difficulty [tiab] OR Serious intellectual difficulty [tiab] OR Severe intellectual difficulty [tiab] OR **Multiple intellectual difficulties** [tiab] OR Complex intellectual difficulties [tiab] OR Profound intellectual difficulties [tiab] OR Serious intellectual difficulties [tiab] OR Severe intellectual difficulties [tiab]

OR Multiple **mental difficulty** [tiab] OR Complex mental difficulty [tiab] OR Profound mental difficulty [tiab] OR Serious mental difficulty [tiab] OR Severe mental difficulty [tiab] OR Multiple **mental difficulties** [tiab] OR Complex mental difficulties [tiab] OR Profound mental difficulties [tiab] OR Serious mental difficulties [tiab] OR Severe mental difficulties [tiab]

OR Complex **neurodevelopmental difficulty** [tiab] OR Multiple neurodevelopmental difficulty [tiab] OR Profound neurodevelopmental difficulty [tiab] OR Serious neurodevelopmental difficulty [tiab] OR Severe neurodevelopmental difficulty [tiab] OR Complex **neurodevelopmental difficulties** [tiab] OR Multiple neurodevelopmental difficulties [tiab] OR Profound neurodevelopmental difficulties [tiab] OR Serious neurodevelopmental difficulties [tiab] OR Severe neurodevelopmental difficulties [tiab]

OR Complex **cognitive disability** [tiab] OR Multiple cognitive disability [tiab] OR Profound cognitive disability [tiab] OR Serious cognitive disability [tiab] OR Severe cognitive disability [tiab] OR Complex **cognitive disabilities** [tiab] OR Multiple cognitive disabilities [tiab] OR Profound cognitive disabilities [tiab] OR Serious cognitive disabilities [tiab] OR Severe cognitive disabilities [tiab]

OR Complex **developmental disability** [tiab] OR Multiple developmental disability [tiab] OR Multiple developmental disability [tiab] OR Serious developmental disability [tiab] OR Severe developmental disability [tiab] OR Complex **developmental disabilities** [tiab] OR Multiple developmental disabilities [tiab] OR Multiple developmental disabilities [tiab] OR Serious developmental disabilities [tiab] OR Severe developmental disabilities [tiab]

OR Trainable **intellectual disabilit**y [tiab] OR Complex intellectual disability [tiab] OR Multiple intellectual disability [tiab] OR Profound intellectual disability [tiab] OR Serious intellectual disability [tiab] OR Severe intellectual disability [tiab] OR Trainable **intellectual disabilit**ies [tiab] OR Complex intellectual disabilities [tiab] OR Multiple intellectual disabilities [tiab] OR Profound intellectual disabilities [tiab] OR Serious intellectual disabilities [tiab] OR Severe intellectual disabilities [tiab]

OR **Severe profound intellectual motor disability** [tiab] OR **Severe profound intellectual motor disabilities** [tiab]

OR Complex **mental disabilit**y [tiab] OR Multiple mental disability [tiab] OR Profound mental disability [tiab] OR Serious mental disability [tiab] OR Severe mental disability [tiab] OR Trainable mental disability [tiab] OR Complex **mental disabilit**ies [tiab] OR Multiple mental disabilities [tiab] OR Profound mental disabilities [tiab] OR Serious mental disabilities [tiab] OR Severe mental disabilities [tiab] OR Trainable mental disabilities [tiab]

OR Complex **neurodevelopmental disabilit**y [tiab] OR Multiple neurodevelopmental disability [tiab] OR Profound neurodevelopmental disability [tiab] OR Serious neurodevelopmental disability [tiab] OR Severe neurodevelopmental disability [tiab] OR Complex **neurodevelopmental disabilit**ies [tiab] OR Multiple neurodevelopmental disabilities [tiab] OR Profound neurodevelopmental disabilities [tiab] OR Serious neurodevelopmental disabilities [tiab] OR Severe neurodevelopmental disabilities [tiab]

OR Multiple disability [tiab] OR Multiple disabilities [tiab]

OR Multiply **cognitively disabled** [tiab] OR Profoundly cognitively disabled [tiab] OR Seriously cognitively disabled [tiab] OR Severely cognitively disabled [tiab]

OR Multiply **developmentally disabled** [tiab] OR Profoundly developmentally disabled [tiab] OR Seriously developmentally disabled [tiab] OR Severely developmentally disabled [tiab] OR Trainable developmentally disabled [tiab] O

R Multiply **intellectually disabled** [tiab] OR Profoundly intellectually disabled [tiab] OR Seriously intellectually disabled [tiab] OR Severely intellectually disabled [tiab] OR Trainable intellectually disabled [tiab]

OR Multiply **mentally disabled** [tiab] OR Profoundly mentally disabled [tiab] OR Seriously mentally disabled [tiab] OR Severely mentally disabled [tiab]

OR Multiply **neurodevelopmentally disabled** [tiab] OR Profoundly neurodevelopmentally disabled [tiab] OR Seriously neurodevelopmentally disabled [tiab] OR Severely neurodevelopmentally disabled [tiab] OR Multiply disabled [tiab] OR Trainable **mentally disabled** [tiab]

OR Complex **developmental disorder*** [tiab] OR Multiple developmental disorder* [tiab] OR Profound developmental disorder* [tiab] OR Serious developmental disorder* [tiab] OR Severe developmental disorder* [tiab]

OR Complex **intellectual disorder** [tiab] OR Multiple intellectual disorder [tiab] OR Profound intellectual disorder [tiab] OR Serious intellectual disorder [tiab] OR Severe intellectual disorder [tiab] OR Complex **intellectual disorders** [tiab] OR Multiple intellectual disorders [tiab] OR Profound intellectual disorders [tiab] OR Serious intellectual disorders [tiab] OR Severe intellectual disorders [tiab]

OR Complex **neurodevelopmental disorder** [tiab] OR Multiple neurodevelopmental disorder [tiab] OR Profound neurodevelopmental disorder [tiab] OR Serious neurodevelopmental disorder [tiab] OR Severe neurodevelopmental disorder [tiab] OR Complex **neurodevelopmental disorder**s [tiab] OR Multiple neurodevelopmental disorders [tiab] OR Profound neurodevelopmental disorders [tiab] OR Serious neurodevelopmental disorders [tiab] OR Severe neurodevelopmental disorders [tiab]

OR Complex **cognitive handicap*** [tiab] OR Multiple cognitive handicap* [tiab] OR Profound cognitive handicap* [tiab] OR Serious cognitive handicap* [tiab] OR Severe cognitive handicap* [tiab]

OR Complex **developmental handicap*** [tiab] OR Multiple developmental handicap* [tiab] OR Profound developmental handicap* [tiab] OR Serious developmental handicap* [tiab] OR Severe developmental handicap* [tiab]

OR Complex **intellectual handicap*** [tiab] OR Multiple intellectual handicap* [tiab] OR Profound intellectual handicap* [tiab] OR Serious intellectual handicap* [tiab] OR Severe intellectual handicap* [tiab]

OR Complex **mental handicap*** [tiab] OR Multiple mental handicap* [tiab] OR Profound mental handicap* [tiab] OR Serious mental handicap* [tiab] OR Severe mental handicap* [tiab] OR Trainable mental handicap* [tiab]

OR Complex **neurodevelopmental handicap*** [tiab] OR Multiple neurodevelopmental handicap* [tiab] OR Profound neurodevelopmental handicap* [tiab] OR Serious neurodevelopmental handicap* [tiab] OR Severe neurodevelopmental handicap* [tiab]

OR Multiply **cognitively handicapped** [tiab] OR Profoundly cognitively handicapped [tiab] OR Seriously cognitively handicapped [tiab] OR Severely cognitively handicapped [tiab]

OR Multiply **developmentally handicapped** [tiab] OR Profoundly developmentally handicapped [tiab] OR Seriously developmentally handicapped [tiab] OR Severely developmentally handicapped [tiab]

OR **Multiply intellectually handicapped** [tiab] OR Profoundly intellectually handicapped [tiab] OR Seriously intellectually handicapped [tiab] OR Severely intellectually handicapped [tiab]

OR Multiply mentally handicapped [tiab] OR Profoundly **mentally handicapped** [tiab] OR Seriously mentally handicapped [tiab] OR Severely mentally handicapped [tiab]

OR Multiply **neurodevelopmentally handicapped** [tiab] OR Profoundly neurodevelopmentally handicapped [tiab] OR Seriously neurodevelopmentally handicapped [tiab] OR Severely neurodevelopmentally handicapped [tiab] OR Multiply handicapped [tiab] OR Trainable mentally handicapped [tiab]

OR Multiply **developmentally impaired** [tiab] OR Profoundly developmentally impaired [tiab] OR Seriously developmentally impaired [tiab] OR Severely developmentally impaired [tiab]

OR Multiply **intellectually impaired** [tiab] OR Profoundly intellectually impaired [tiab] OR Seriously intellectually impaired [tiab] OR Severely intellectually impaired [tiab]

OR Profoundly **mentally impaired** [tiab] OR Seriously mentally impaired [tiab] OR Severely mentally impaired [tiab]

OR Multiply **neurodevelopmentally impaired** [tiab] OR Profoundly neurodevelopmentally impaired [tiab] OR Seriously neurodevelopmentally impaired [tiab] OR Severely neurodevelopmentally impaired [tiab] OR Trainable mentally impaired [tiab] OR Complex developmental impairment* [tiab]

OR Multiple **developmental impairment*** [tiab] OR Profound developmental impairment* [tiab] OR Serious developmental impairment* [tiab] OR Severe developmental impairment* [tiab]

OR Complex **intellectual impairment*** [tiab] OR Multiple intellectual impairment* [tiab] OR Profound intellectual impairment* [tiab] OR Serious intellectual impairment* [tiab] OR Severe intellectual impairment* [tiab]

OR Complex **mental impairment*** [tiab] OR Multiple mental impairment* [tiab] OR Profound mental impairment* [tiab] OR Serious mental impairment* [tiab] OR Severe mental impairment* [tiab]

OR Complex **neurodevelopmental impairment*** [tiab] OR Multiple neurodevelopmental impairment* [tiab] OR Profound neurodevelopmental impairment* [tiab] OR Serious neurodevelopmental impairment* [tiab] OR Severe neurodevelopmental impairment* [tiab] OR Trainable mental impairment* [tiab]

OR Complex **cognitive incapacit*** [tiab] OR Multiple cognitive incapacit* [tiab] OR Profound cognitive incapacit* [tiab] OR Serious cognitive incapacit* [tiab] OR Severe cognitive incapacit* [tiab]

OR Complex **developmental incapacit*** [tiab] OR Multiple developmental incapacit* [tiab] OR Profound developmental incapacit* [tiab] OR Serious developmental incapacit* [tiab] OR Severe developmental incapacit* [tiab]

OR **Complex intellectual incapacit*** [tiab] OR Multiple intellectual incapacit* [tiab] OR Profound intellectual incapacit* [tiab] OR Serious intellectual incapacit* [tiab] OR Severe intellectual incapacit* [tiab]

OR Complex **mental incapacit*** [tiab] OR Multiple mental incapacit* [tiab] OR Profound mental incapacit* [tiab] OR Serious mental incapacit* [tiab] OR Severe mental incapacit* [tiab]

OR Complex **neurodevelopmental incapacit*** [tiab] OR Multiple neurodevelopmental incapacit* [tiab] OR Profound neurodevelopmental incapacit* [tiab] OR Serious neurodevelopmental incapacit* [tiab] OR Severe neurodevelopmental incapacit* [tiab]

OR Multiply **cognitively incapacitated** [tiab] OR Profoundly cognitively incapacitated [tiab] OR Seriously cognitively incapacitated [tiab] OR Severely cognitively incapacitated [tiab]

OR Multiply **developmentally incapacitated** [tiab] OR Profoundly developmentally incapacitated [tiab] OR Seriously developmentally incapacitated [tiab] OR Severely developmentally incapacitated [tiab]

OR Multiply **intellectually incapacitated** [tiab] OR Profoundly intellectually incapacitated [tiab] OR Seriously intellectually incapacitated [tiab] OR Severely intellectually incapacitated [tiab]

OR Multiply **mentally incapacitated** [tiab] OR Profoundly mentally incapacitated [tiab] OR Seriously mentally incapacitated [tiab] OR Severely mentally incapacitated [tiab]

OR Multiply **neurodevelopmentally incapacitated** [tiab] OR Profoundly neurodevelopmentally incapacitated [tiab] OR Seriously neurodevelopmentally incapacitated [tiab] OR Severely neurodevelopmentally incapacitated [tiab]

OR Polyhandicap [tiab] OR Polyhandicaps [tiab] OR Trainable mentally retardate* [tiab]

OR Complex **cognitive retardation** [tiab] OR Multiple cognitive retardation [tiab] OR Profound cognitive retardation [tiab] OR Serious cognitive retardation [tiab] OR Severe cognitive retardation [tiab]

OR Complex **developmental retardation** [tiab] OR Multiple developmental retardation [tiab] OR Profound developmental retardation [tiab] OR Serious developmental retardation [tiab] OR Severe developmental retardation [tiab]

OR Complex **intellectual retardation** [tiab] OR Multiple intellectual retardation [tiab] OR Profound intellectual retardation [tiab] OR Serious intellectual retardation [tiab] OR Severe intellectual retardation [tiab]

OR Complex **mental retardation** [tiab] OR Multiple mental retardation [tiab] OR Profound mental retardation [tiab] OR Serious mental retardation [tiab] OR Severe mental retardation [tiab] OR Trainable mental retardation [tiab]

OR Complex **neurodevelopmental retardation** [tiab] OR Multiple neurodevelopmental retardation [tiab] OR Profound neurodevelopmental retardation [tiab] OR Serious neurodevelopmental retardation [tiab] OR Severe neurodevelopmental retardation [tiab]

OR Multiply **cognitively retarded** [tiab] OR Profoundly cognitively retarded [tiab] OR Seriously cognitively retarded [tiab] OR Severely cognitively retarded [tiab]

OR Multiply **developmentally retarded** [tiab] OR Profoundly developmentally retarded [tiab] OR Seriously developmentally retarded [tiab] OR Severely developmentally retarded [tiab]

OR **Multiply intellectually retarded** [tiab] OR Profoundly intellectually retarded [tiab] OR Seriously intellectually retarded [tiab] OR Severely intellectually retarded [tiab]

OR Multiply **mentally retarded** [tiab] OR Profoundly mentally retarded [tiab] OR Seriously mentally retarded [tiab] OR Severely mentally retarded [tiab]

OR Multiply **neurodevelopmentally retarded** [tiab] OR Profoundly neurodevelopmentally retarded [tiab] OR Seriously neurodevelopmentally retarded [tiab] OR Severely neurodevelopmentally retarded [tiab] OR Trainable mentally retarded [tiab] OR Trainable retarded [tiab])

AND

(“patient acuity”[MeSH] OR “patient acuity”[tiab] OR “Severity of illness”[tiab] “illness severit*”[tiab] or “disease severit*”[tiab]OR “sickness”[tiab] OR “Patient Reported Outcome Measures”[MeSH] OR “patient reported outcome*”[tiab]

OR “Pain”[MeSH] OR “pain*”[tiab] OR “Comorbidity”[MeSH Terms] OR “comorbidit*”[tiab] OR “medical condition*”[tiab] OR “clinical characteristic*”[tiab] OR “health status”[tiab] OR “physical condition*”[tiab] OR “chronic disease*”[tiab] OR "health problem*"[tiab] OR "health indicator*"[tiab] OR "health issue*"[tiab] OR "secondary condition*"[tiab] OR "physical health*"[tiab] OR

“Enteral nutrition”[MeSH] OR “enteral nutrition”[tiab] OR “nutritional deficienc*”[tiab] OR “tube feed*”[tiab] OR “tube fed*”[tiab] OR “enteral feeding”[tiab] OR “gastrostomy”[MeSH] OR “gastrostom*”[tiab]

OR “musculoskeletal diseases”[MeSH] OR “orthopedic disorder*”[tiab] OR “orthopaedic disorder*”[tiab] OR “scoliosis”[MeSH] OR “scoliosis”[tiab]

OR “Epilepsy”[MeSH] OR “epilepsy”[tiab] OR “seizures”[tiab] OR “osteoporosis”[MeSH] OR “osteoporos*”[tiab] OR “dyssomnias”[MeSH] OR “sleep problem*”[tiab] OR “sleep disorder*”[tiab] OR "Vision Disorders"[Mesh] OR “visual impairment”[tiab] OR "Hearing Loss"[Mesh] OR “hearing impairment”[tiab] OR “hearing disorder*”[tiab] OR “Dental Care”[MeSH] OR “dental care”[tiab] OR “dental health”[tiab] OR “dental problem*”[tiab] OR "Urinary Incontinence"[Mesh] OR “urine incontinence”[tiab] OR "Constipation"[Mesh] OR “constipation”[tiab]

OR “Respiratory Tract Infections”[MeSH] OR “Respiratory infection*”[tiab] OR “respiratory tract infection*”[tiab] OR “Body Size”[MeSH] OR underweight[tiab] OR overweight[tiab] OR Pressure Ulcer[MeSH] OR decubitus[tiab] OR “pressure ulcer”[tiab] OR Muscle Spasticity[MeSH] OR “muscle spasticit*”[tiab] OR “muscle spasm*”[tiab]

OR “gastroesophageal reflux”[MeSH] OR “gastroesophageal reflux”[tiab] OR dysphagia[tiab] OR Sialorrhea[Mesh] OR Sialorrhea[tiab] OR drooling[tiab] OR Hypertension[MeSH] OR hypertension[tiab]

OR Endocrine System Diseases[MeSH] OR “endocrine disorder*”[tiab] OR “Endocrine disease*”[tiab] OR Metabolic Syndrome[MeSH] OR “metabolic syndrome”[tiab] OR Menstrual Cycle[MeSH] OR “menstrual cycle”[tiab] OR "Heart Defects, Congenital"[Mesh] OR “congenital heart defect*”[tiab])

**PsychInfo Search**

((Complex adj2 **developmental abnormalit**y).ti,ab,id. OR (Multiple adj2 developmental abnormality).ti,ab,id. OR (Profound adj2 developmental abnormality).ti,ab,id. OR (Serious adj2 developmental abnormality).ti,ab,id. OR (Severe adj2 developmental abnormality).ti,ab,id. OR (Complex adj2 **developmental abnormalit**ies).ti,ab,id. OR (Multiple adj2 developmental abnormalities).ti,ab,id. OR (Profound adj2 developmental abnormalities).ti,ab,id. OR (Serious adj2 developmental abnormalities).ti,ab,id. OR (Severe adj2 developmental abnormalities).ti,ab,id.

OR (**Complex adj2 intellectual abnormality**).ti,ab,id. OR (Multiple adj2 intellectual abnormality).ti,ab,id. OR (Profound adj2 intellectual abnormality).ti,ab,id. OR (Serious adj2 intellectual abnormality).ti,ab,id. OR (Severe adj2 intellectual abnormality).ti,ab,id. OR (**Complex adj2 intellectual abnormalities**).ti,ab,id. OR (Multiple adj2 intellectual abnormalities).ti,ab,id. OR (Profound adj2 intellectual abnormalities).ti,ab,id. OR (Serious adj2 intellectual abnormalities).ti,ab,id. OR (Severe adj2 intellectual abnormalities).ti,ab,id.

OR (Complex adj2 **mental abnormality**).ti,ab,id. OR (Multiple adj2 mental abnormality).ti,ab,id. OR (Profound adj2 mental abnormality).ti,ab,id. OR (Serious adj2 mental abnormality).ti,ab,id. OR (Severe adj2 mental abnormality).ti,ab,id. OR (Complex adj2 **mental abnormalities**).ti,ab,id. OR (Multiple adj2 mental abnormalities).ti,ab,id. OR (Profound adj2 mental abnormalities).ti,ab,id. OR (Serious adj2 mental abnormalities).ti,ab,id. OR (Severe adj2 mental abnormalities).ti,ab,id.

OR (Complex adj2 **neurodevelopmental abnormality**).ti,ab,id. OR (Multiple adj2 neurodevelopmental abnormality).ti,ab,id. OR (Profound adj2 neurodevelopmental abnormality).ti,ab,id. OR (Serious adj2 neurodevelopmental abnormality).ti,ab,id. OR (Severe adj2 neurodevelopmental abnormality).ti,ab,id. OR (Complex adj2 **neurodevelopmental abnormalities**).ti,ab,id. OR (Multiple adj2 neurodevelopmental abnormalities).ti,ab,id. OR (Profound adj2 neurodevelopmental abnormalities).ti,ab,id. OR (Serious adj2 neurodevelopmental abnormalities).ti,ab,id. OR (Severe adj2 neurodevelopmental abnormalities).ti,ab,id.

OR (Complex adj2 **cognitive defect**).ti,ab,id. OR (Multiple adj2 cognitive defect).ti,ab,id. OR (Profound adj2 cognitive defect).ti,ab,id. OR (Serious adj2 cognitive defect).ti,ab,id. OR (Severe adj2 cognitive defect).ti,ab,id. OR (Complex adj2 **cognitive defect**s).ti,ab,id. OR (Multiple adj2 cognitive defects).ti,ab,id. OR (Profound adj2 cognitive defects).ti,ab,id. OR (Serious adj2 cognitive defects).ti,ab,id. OR (Severe adj2 cognitive defects).ti,ab,id.

OR (Complex adj2 **developmental defect**).ti,ab,id. OR (Multiple adj2 developmental defect).ti,ab,id. OR (Profound adj2 developmental defect).ti,ab,id. OR (Serious adj2 developmental defect).ti,ab,id. OR (Severe adj2 developmental defect*).ti,ab,id. OR (Complex adj2 **developmental defects**).ti,ab,id. OR (Multiple adj2 developmental defects).ti,ab,id. OR (Profound adj2 developmental defects).ti,ab,id. OR (Serious adj2 developmental defects).ti,ab,id. OR (Severe adj2 developmental defects).ti,ab,id.

OR (Complex adj2 **intellectual defect**).ti,ab,id. OR (Multiple adj2 intellectual defect).ti,ab,id. OR (Profound adj2 intellectual defect).ti,ab,id. OR (Serious adj2 intellectual defect).ti,ab,id. OR (Severe adj2 intellectual defect).ti,ab,id. OR (Complex adj2 **intellectual defects**).ti,ab,id. OR (Multiple adj2 intellectual defects).ti,ab,id. OR (Profound adj2 intellectual defects).ti,ab,id. OR (Serious adj2 intellectual defects).ti,ab,id. OR (Severe adj2 intellectual defects).ti,ab,id.

OR (Complex adj2 **mental defect**).ti,ab,id. OR (Multiple adj2 mental defect).ti,ab,id. OR (Profound adj2 mental defect).ti,ab,id. OR (Serious adj2 mental defect).ti,ab,id. OR (Severe adj2 mental defect).ti,ab,id. OR (Complex adj2 **mental defects**).ti,ab,id. OR (Multiple adj2 mental defects).ti,ab,id. OR (Profound adj2 mental defects).ti,ab,id. OR (Serious adj2 mental defects).ti,ab,id. OR (Severe adj2 mental defects).ti,ab,id.

OR (Complex adj2 **neurodevelopmental defect**).ti,ab,id. OR (Multiple adj2 neurodevelopmental defect).ti,ab,id. OR (Profound adj2 neurodevelopmental defect).ti,ab,id. OR (Serious adj2 neurodevelopmental defect).ti,ab,id. OR (Severe adj2 neurodevelopmental defect).ti,ab,id. OR (Complex adj2 **neurodevelopmental defect**s).ti,ab,id. OR (Multiple adj2 neurodevelopmental defects).ti,ab,id. OR (Profound adj2 neurodevelopmental defects).ti,ab,id. OR (Serious adj2 neurodevelopmental defects).ti,ab,id. OR (Severe adj2 neurodevelopmental defects).ti,ab,id.

OR (Multiply adj2 **cognitively defective**).ti,ab,id. OR (Profoundly adj2 cognitively defective).ti,ab,id. OR (Seriously adj2 cognitively defective).ti,ab,id. OR (Severely adj2 cognitively defective).ti,ab,id.

OR (Multiply adj2 **developmentally defective**).ti,ab,id. OR (Profoundly adj2 developmentally defective).ti,ab,id. OR (Seriously adj2 developmentally defective).ti,ab,id. OR (Severely adj2 developmentally defective).ti,ab,id.

OR (Multiply adj2 **intellectually defective**).ti,ab,id. OR (Profoundly adj2 intellectually defective).ti,ab,id. OR (Seriously adj2 intellectually defective).ti,ab,id. OR (Severely adj2 intellectually defective).ti,ab,id.

OR (Profoundly adj2 **mentally defective**).ti,ab,id. OR (Seriously adj2 mentally defective).ti,ab,id. OR (Severely adj2 mentally defective).ti,ab,id.

OR (Multiply adj2 **neurodevelopmentally defectiv**e).ti,ab,id. OR (Profoundly adj2 neurodevelopmentally defective).ti,ab,id. OR (Seriously adj2 neurodevelopmentally defective).ti,ab,id. OR (Severely adj2 neurodevelopmentally defective).ti,ab,id.

OR (Complex adj2 **cognitive deficienc***).ti,ab,id. OR (Multiple adj2 cognitive deficienc*).ti,ab,id. OR (Profound adj2 cognitive deficienc*).ti,ab,id. OR (Serious adj2 cognitive deficienc*).ti,ab,id. OR (Severe adj2 cognitive deficienc*).ti,ab,id.

OR (Complex adj2 **developmental deficienc***).ti,ab,id. OR (Multiple adj2 developmental deficienc*).ti,ab,id. OR (Profound adj2 developmental deficienc*).ti,ab,id. OR (Serious adj2 developmental deficienc*).ti,ab,id. OR (Severe adj2 developmental deficienc*).ti,ab,id.

OR (Complex adj2 **intellectual deficienc***).ti,ab,id. OR (Multiple adj2 intellectual deficienc*).ti,ab,id. OR (Profound adj2 intellectual deficienc*).ti,ab,id. OR (Serious adj2 intellectual deficienc*).ti,ab,id. OR (Severe adj2 intellectual deficienc*).ti,ab,id.

OR (Complex adj2 **mental deficienc***).ti,ab,id. OR (Multiple adj2 mental deficienc*).ti,ab,id. OR (Profound adj2 mental deficienc*).ti,ab,id. OR (Serious adj2 mental deficienc*).ti,ab,id. OR (Severe adj2 mental deficienc*).ti,ab,id.

OR (Complex adj2 **neurodevelopmental deficienc***).ti,ab,id. OR (Multiple adj2 neurodevelopmental deficienc*).ti,ab,id. OR (Profound adj2 neurodevelopmental deficienc*).ti,ab,id. OR (Serious adj2 neurodevelopmental deficienc*).ti,ab,id. OR (Severe adj2 neurodevelopmental deficienc*).ti,ab,id.

OR (Multiply adj2 **cognitively deficient**).ti,ab,id. OR (Profoundly adj2 cognitively deficient).ti,ab,id. OR (Seriously adj2 cognitively deficient).ti,ab,id. OR (Severely adj2 cognitively deficient).ti,ab,id.

OR (Multiply adj2 **developmentally deficient).ti,ab,id. OR (**Profoundly adj2 developmentally deficient).ti,ab,id. OR (Seriously adj2 developmentally deficient).ti,ab,id. OR (Severely adj2 developmentally deficient).ti,ab,id.

OR (Multiply adj2 **intellectually deficient**).ti,ab,id. OR (Profoundly adj2 intellectually deficient).ti,ab,id. OR (Seriously adj2 intellectually deficient).ti,ab,id. OR (Severely adj2 intellectually deficient).ti,ab,id.

OR (Multiply adj2 **mentally deficient**).ti,ab,id. OR (Profoundly adj2 mentally deficient).ti,ab,id. OR (Seriously adj2 mentally deficient).ti,ab,id. OR (Severely adj2 mentally deficient).ti,ab,id.

OR (Multiply adj2 **neurodevelopmentally deficient**).ti,ab,id. OR (Profoundly adj2 neurodevelopmentally deficient).ti,ab,id. OR (Seriously adj2 neurodevelopmentally deficient).ti,ab,id. OR (Severely adj2 neurodevelopmentally deficient).ti,ab,id.

OR (**Complex adj2 developmental deficit***).ti,ab,id. OR (Multiple adj2 developmental deficit*).ti,ab,id. OR (Profound adj2 developmental deficit*).ti,ab,id. OR (Serious adj2 developmental deficit*).ti,ab,id. OR (Severe adj2 developmental deficit*).ti,ab,id.

OR (Complex adj2 **intellectual deficit***).ti,ab,id. OR (Multiple adj2 intellectual deficit*).ti,ab,id. OR (Profound adj2 intellectual deficit*).ti,ab,id. OR (Serious adj2 intellectual deficit*).ti,ab,id. OR (Severe adj2 intellectual deficit*).ti,ab,id.

OR (Complex adj2 **learning deficit***).ti,ab,id. OR (Multiple adj2 learning deficit*).ti,ab,id. OR (Profound adj2 learning deficit*).ti,ab,id. OR (Serious adj2 learning deficit*).ti,ab,id. OR (Severe adj2 learning deficit*).ti,ab,id.

OR (Complex adj2 **mental deficit*).ti,ab,id. OR (**Multiple adj2 mental deficit*).ti,ab,id. OR (Profound adj2 mental deficit*).ti,ab,id. OR (Serious adj2 mental deficit*).ti,ab,id. OR (Severe adj2 mental deficit*).ti,ab,id.

OR (Complex adj2 **neurodevelopmental deficit***).ti,ab,id. OR (Multiple adj2 neurodevelopmental deficit*).ti,ab,id. OR (Profound adj2 neurodevelopmental deficit*).ti,ab,id. OR (Serious adj2 neurodevelopmental deficit*).ti,ab,id. OR (Severe adj2 neurodevelopmental deficit*).ti,ab,id.

OR (Complex adj2 **cognitive delay**).ti,ab,id. OR (Multiple adj2 cognitive delay).ti,ab,id. OR (Profound adj2 cognitive delay).ti,ab,id. OR (Serious adj2 cognitive delay).ti,ab,id. OR (Severe adj2 cognitive delay).ti,ab,id.

OR (Complex adj2 **developmental delay**).ti,ab,id. OR (Multiple adj2 developmental delay).ti,ab,id. OR (Profound adj2 developmental delay).ti,ab,id. OR (Serious adj2 developmental delay).ti,ab,id. OR (Severe adj2 developmental delay).ti,ab,id.

OR (Complex adj2 **intellectual delay**).ti,ab,id. OR (Multiple adj2 intellectual delay).ti,ab,id. OR (Profound adj2 intellectual delay).ti,ab,id. OR (Serious adj2 intellectual delay).ti,ab,id. OR (Severe adj2 intellectual delay).ti,ab,id.

OR (Complex adj2 **mental delay**).ti,ab,id. OR (Multiple adj2 mental delay).ti,ab,id. OR (Profound adj2 mental delay).ti,ab,id. OR (Serious adj2 mental delay).ti,ab,id. OR (Severe adj2 mental delay).ti,ab,id.

OR (Complex adj2 **neurodevelopmental delay**).ti,ab,id. OR (Multiple adj2 neurodevelopmental delay).ti,ab,id. OR (Profound adj2 neurodevelopmental delay).ti,ab,id. OR (Serious adj2 neurodevelopmental delay).ti,ab,id. OR (Severe adj2 neurodevelopmental delay).ti,ab,id.

OR (Multiply adj2 **cognitively delayed**).ti,ab,id. OR (Profoundly adj2 cognitively delayed).ti,ab,id. OR (Seriously adj2 cognitively delayed).ti,ab,id. OR (Severely adj2 cognitively delayed).ti,ab,id.

OR (Multiply adj2 **developmentally delayed**).ti,ab,id. OR (Profoundly adj2 developmentally delayed).ti,ab,id. OR (Seriously adj2 developmentally delayed).ti,ab,id. OR (Severely adj2 developmentally delayed).ti,ab,id.

OR (Multiply adj2 **intellectually delayed**).ti,ab,id. OR (Profoundly adj2 intellectually delayed).ti,ab,id. OR (Seriously adj2 intellectually delayed).ti,ab,id. OR (Severely adj2 intellectually delayed).ti,ab,id.

OR (Multiply adj2 **mentally delayed).ti,ab,id. OR (**Profoundly adj2 mentally delayed).ti,ab,id. OR (Seriously adj2 mentally delayed).ti,ab,id. OR (Severely adj2 mentally delayed).ti,ab,id.

OR (Multiply adj2 **neurodevelopmentally delayed).ti,ab,id. OR (**Profoundly adj2 neurodevelopmentally delayed).ti,ab,id. OR (Seriously adj2 neurodevelopmentally delayed).ti,ab,id. OR (Severely adj2 neurodevelopmentally delayed).ti,ab,id.

OR (Multiply adj2 **differently-abled**).ti,ab,id. OR (Profoundly adj2 differently-abled).ti,ab,id. OR (Seriously adj2 differently-abled).ti,ab,id. OR (Severely adj2 differently-abled).ti,ab,id.

OR (Multiple adj2 **developmental difficult**y).ti,ab,id. OR (Complex adj2 developmental difficulty).ti,ab,id. OR (Profound adj2 developmental difficulty).ti,ab,id. OR (Serious adj2 developmental difficulty).ti,ab,id. OR (Severe adj2 developmental difficulty).ti,ab,id. OR (Multiple adj2 **developmental difficult**ies).ti,ab,id. OR (Complex adj2 developmental difficulties).ti,ab,id. OR (Profound adj2 developmental difficulties).ti,ab,id. OR (Serious adj2 developmental difficulties).ti,ab,id. OR (Severe adj2 developmental difficulties).ti,ab,id.

OR (**Multiple adj2 intellectual difficulty**).ti,ab,id. OR (Complex adj2 intellectual difficulty).ti,ab,id. OR (Profound adj2 intellectual difficulty).ti,ab,id. OR (Serious adj2 intellectual difficulty).ti,ab,id. OR (Severe adj2 intellectual difficulty).ti,ab,id. OR (**Multiple adj2 intellectual difficulties**).ti,ab,id. OR (Complex adj2 intellectual difficulties).ti,ab,id. OR (Profound adj2 intellectual difficulties).ti,ab,id. OR (Serious adj2 intellectual difficulties).ti,ab,id. OR (Severe adj2 intellectual difficulties).ti,ab,id.

OR (Multiple adj2 **mental difficulty**).ti,ab,id. OR (Complex adj2 mental difficulty).ti,ab,id. OR (Profound adj2 mental difficulty).ti,ab,id. OR (Serious adj2 mental difficulty).ti,ab,id. OR (Severe adj2 mental difficulty).ti,ab,id. OR (Multiple adj2 **mental difficulties**).ti,ab,id. OR (Complex adj2 mental difficulties).ti,ab,id. OR (Profound adj2 mental difficulties).ti,ab,id. OR (Serious adj2 mental difficulties).ti,ab,id. OR (Severe adj2 mental difficulties).ti,ab,id.

OR (Complex adj2 **neurodevelopmental difficulty**).ti,ab,id. OR (Multiple adj2 neurodevelopmental difficulty).ti,ab,id. OR (Profound adj2 neurodevelopmental difficulty).ti,ab,id. OR (Serious adj2 neurodevelopmental difficulty).ti,ab,id. OR (Severe adj2 neurodevelopmental difficulty).ti,ab,id. OR (Complex adj2 **neurodevelopmental difficulties**).ti,ab,id. OR (Multiple adj2 neurodevelopmental difficulties).ti,ab,id. OR (Profound adj2 neurodevelopmental difficulties).ti,ab,id. OR (Serious adj2 neurodevelopmental difficulties).ti,ab,id. OR (Severe adj2 neurodevelopmental difficulties).ti,ab,id.

OR (Complex adj2 **cognitive disability**).ti,ab,id. OR (Multiple adj2 cognitive disability).ti,ab,id. OR (Profound adj2 cognitive disability).ti,ab,id. OR (Serious adj2 cognitive disability).ti,ab,id. OR (Severe adj2 cognitive disability).ti,ab,id. OR (Complex adj2 **cognitive disabilities**).ti,ab,id. OR (Multiple adj2 cognitive disabilities).ti,ab,id. OR (Profound adj2 cognitive disabilities).ti,ab,id. OR (Serious adj2 cognitive disabilities).ti,ab,id. OR (Severe adj2 cognitive disabilities).ti,ab,id.

OR (Complex adj2 **developmental disability**).ti,ab,id. OR (Multiple adj2 developmental disability).ti,ab,id. OR (Multiple adj2 developmental disability).ti,ab,id. OR (Serious adj2 developmental disability).ti,ab,id. OR (Severe adj2 developmental disability).ti,ab,id. OR (Complex adj2 **developmental disabilities**).ti,ab,id. OR (Multiple adj2 developmental disabilities).ti,ab,id. OR (Multiple adj2 developmental disabilities).ti,ab,id. OR (Serious adj2 developmental disabilities).ti,ab,id. OR (Severe adj2 developmental disabilities).ti,ab,id.

OR (Trainable adj2 **intellectual disabilit**y).ti,ab,id. OR (Complex adj2 intellectual disability).ti,ab,id. OR (Multiple adj2 intellectual disability).ti,ab,id. OR (Profound adj2 intellectual disability).ti,ab,id. OR (Serious adj2 intellectual disability).ti,ab,id. OR (Severe adj2 intellectual disability).ti,ab,id. OR (Trainable adj2 **intellectual disabilit**ies).ti,ab,id. OR (Complex adj2 intellectual disabilities).ti,ab,id. OR (Multiple adj2 intellectual disabilities).ti,ab,id. OR (Profound adj2 intellectual disabilities).ti,ab,id. OR (Serious adj2 intellectual disabilities).ti,ab,id. OR (Severe adj2 intellectual disabilities).ti,ab,id.

OR (**Severe adj2 Profound adj2 intellectual motor disability**).ti,ab,id. OR (**Severe adj2 Profound adj2 intellectual motor disabilities)**.ti,ab,id.

OR (Complex adj2 **mental disabilit**y).ti,ab,id. OR (Multiple adj2 mental disability).ti,ab,id. OR (Profound adj2 mental disability).ti,ab,id. OR (Serious adj2 mental disability).ti,ab,id. OR (Severe adj2 mental disability).ti,ab,id. OR (Trainable adj2 mental disability).ti,ab,id. OR (Complex adj2 **mental disabilit**ies).ti,ab,id. OR (Multiple adj2 mental disabilities).ti,ab,id. OR (Profound adj2 mental disabilities).ti,ab,id. OR (Serious adj2 mental disabilities).ti,ab,id. OR (Severe adj2 mental disabilities).ti,ab,id. OR (Trainable adj2 mental disabilities).ti,ab,id.

OR (Complex adj2 **neurodevelopmental disabilit**y).ti,ab,id. OR (Multiple adj2 neurodevelopmental disability).ti,ab,id. OR (Profound adj2 neurodevelopmental disability).ti,ab,id. OR (Serious adj2 neurodevelopmental disability).ti,ab,id. OR (Severe adj2 neurodevelopmental disability).ti,ab,id. OR (Complex adj2 **neurodevelopmental disabilit**ies).ti,ab,id. OR (Multiple adj2 neurodevelopmental disabilities).ti,ab,id. OR (Profound adj2 neurodevelopmental disabilities).ti,ab,id. OR (Serious adj2 neurodevelopmental disabilities).ti,ab,id. OR (Severe adj2 neurodevelopmental disabilities).ti,ab,id.

OR (Multiple adj2 disability).ti,ab,id. OR (Multiple adj2 disabilities).ti,ab,id.

OR (Multiply adj2 **cognitively disabled**).ti,ab,id. OR (Profoundly adj2 cognitively disabled).ti,ab,id. OR (Seriously adj2 cognitively disabled).ti,ab,id. OR (Severely adj2 cognitively disabled).ti,ab,id.

OR (Multiply adj2 **developmentally disabled**).ti,ab,id. OR (Profoundly adj2 developmentally disabled).ti,ab,id. OR (Seriously adj2 developmentally disabled).ti,ab,id. OR (Severely adj2 developmentally disabled).ti,ab,id. OR (Trainable adj2 developmentally disabled).ti,ab,id.

OR (Multiply adj2 **intellectually disabled**).ti,ab,id. OR (Profoundly adj2 intellectually disabled).ti,ab,id. OR (Seriously adj2 intellectually disabled).ti,ab,id. OR (Severely adj2 intellectually disabled).ti,ab,id. OR (Trainable adj2 intellectually disabled).ti,ab,id.

OR (Multiply adj2 **mentally disabled**).ti,ab,id. OR (Profoundly adj2 mentally disabled).ti,ab,id. OR (Seriously adj2 mentally disabled).ti,ab,id. OR (Severely adj2 mentally disabled).ti,ab,id.

OR (Multiply adj2 **neurodevelopmentally disabled**).ti,ab,id. OR (Profoundly adj2 neurodevelopmentally disabled).ti,ab,id. OR (Seriously adj2 neurodevelopmentally disabled).ti,ab,id. OR (Severely adj2 neurodevelopmentally disabled).ti,ab,id. OR (Multiply adj2 disabled).ti,ab,id. OR (Trainable adj2 **mentally disabled)**.ti,ab,id.

OR (Complex adj2 **developmental disorder***).ti,ab,id. OR (Multiple adj2 developmental disorder*).ti,ab,id. OR (Profound adj2 developmental disorder*).ti,ab,id. OR (Serious adj2 developmental disorder*).ti,ab,id. OR (Severe adj2 developmental disorder*).ti,ab,id.

OR (Complex adj2 **intellectual disorder**).ti,ab,id. OR (Multiple adj2 intellectual disorder).ti,ab,id. OR (Profound adj2 intellectual disorder).ti,ab,id. OR (Serious adj2 intellectual disorder).ti,ab,id. OR (Severe adj2 intellectual disorder).ti,ab,id. OR (Complex adj2 **intellectual disorders**).ti,ab,id. OR (Multiple adj2 intellectual disorders).ti,ab,id. OR (Profound adj2 intellectual disorders).ti,ab,id. OR (Serious adj2 intellectual disorders).ti,ab,id. OR (Severe adj2 intellectual disorders).ti,ab,id.

OR (Complex adj2 **neurodevelopmental disorder**).ti,ab,id. OR (Multiple adj2 neurodevelopmental disorder).ti,ab,id. OR (Profound adj2 neurodevelopmental disorder).ti,ab,id. OR (Serious adj2 neurodevelopmental disorder).ti,ab,id. OR (Severe adj2 neurodevelopmental disorder).ti,ab,id. OR (Complex adj2 **neurodevelopmental disorder**s).ti,ab,id. OR (Multiple adj2 neurodevelopmental disorders).ti,ab,id. OR (Profound adj2 neurodevelopmental disorders).ti,ab,id. OR (Serious adj2 neurodevelopmental disorders).ti,ab,id. OR (Severe adj2 neurodevelopmental disorders).ti,ab,id.

OR (Complex adj2 **cognitive handicap***).ti,ab,id. OR (Multiple adj2 cognitive handicap*).ti,ab,id. OR (Profound adj2 cognitive handicap*).ti,ab,id. OR (Serious adj2 cognitive handicap*).ti,ab,id. OR (Severe adj2 cognitive handicap*).ti,ab,id.

OR (Complex adj2 **developmental handicap***).ti,ab,id. OR (Multiple adj2 developmental handicap*).ti,ab,id. OR (Profound adj2 developmental handicap*).ti,ab,id. OR (Serious adj2 developmental handicap*).ti,ab,id. OR (Severe adj2 developmental handicap*).ti,ab,id.

OR (Complex adj2 **intellectual handicap***).ti,ab,id. OR (Multiple adj2 intellectual handicap*).ti,ab,id. OR (Profound adj2 intellectual handicap*).ti,ab,id. OR (Serious adj2 intellectual handicap*).ti,ab,id. OR (Severe adj2 intellectual handicap*).ti,ab,id.

OR (Complex adj2 **mental handicap***).ti,ab,id. OR (Multiple adj2 mental handicap*).ti,ab,id. OR (Profound adj2 mental handicap*).ti,ab,id. OR (Serious adj2 mental handicap*).ti,ab,id. OR (Severe adj2 mental handicap*).ti,ab,id. OR (Trainable adj2 mental handicap*).ti,ab,id.

OR (Complex adj2 **neurodevelopmental handicap***).ti,ab,id. OR (Multiple adj2 neurodevelopmental handicap*).ti,ab,id. OR (Profound adj2 neurodevelopmental handicap*).ti,ab,id. OR (Serious adj2 neurodevelopmental handicap*).ti,ab,id. OR (Severe adj2 neurodevelopmental handicap*).ti,ab,id.

OR (Multiply adj2 **cognitively handicapped**).ti,ab,id. OR (Profoundly adj2 cognitively handicapped).ti,ab,id. OR (Seriously adj2 cognitively handicapped).ti,ab,id. OR (Severely adj2 cognitively handicapped).ti,ab,id.

OR (Multiply adj2 **developmentally handicapped**).ti,ab,id. OR (Profoundly adj2 developmentally handicapped).ti,ab,id. OR (Seriously adj2 developmentally handicapped).ti,ab,id. OR (Severely adj2 developmentally handicapped).ti,ab,id.

OR (**Multiply adj2 intellectually handicapped**).ti,ab,id. OR (Profoundly adj2 intellectually handicapped).ti,ab,id. OR (Seriously adj2 intellectually handicapped).ti,ab,id. OR (Severely adj2 intellectually handicapped).ti,ab,id.

OR (Multiply adj2 mentally handicapped).ti,ab,id. OR (Profoundly adj2 **mentally handicapped**).ti,ab,id. OR (Seriously adj2 mentally handicapped).ti,ab,id. OR (Severely adj2 mentally handicapped).ti,ab,id.

OR (Multiply adj2 **neurodevelopmentally handicapped**).ti,ab,id. OR (Profoundly adj2 neurodevelopmentally handicapped).ti,ab,id. OR (Seriously adj2 neurodevelopmentally handicapped).ti,ab,id. OR (Severely adj2 neurodevelopmentally handicapped).ti,ab,id. OR (Multiply adj2 handicapped).ti,ab,id. OR (Trainable adj2 mentally handicapped).ti,ab,id.

OR (Multiply adj2 **developmentally impaired**).ti,ab,id. OR (Profoundly adj2 developmentally impaired).ti,ab,id. OR (Seriously adj2 developmentally impaired).ti,ab,id. OR (Severely adj2 developmentally impaired).ti,ab,id.

OR (Multiply adj2 **intellectually impaired**).ti,ab,id. OR (Profoundly adj2 intellectually impaired).ti,ab,id. OR (Seriously adj2 intellectually impaired).ti,ab,id. OR (Severely adj2 intellectually impaired).ti,ab,id.

OR (Profoundly adj2 **mentally impaired**).ti,ab,id. OR (Seriously adj2 mentally impaired).ti,ab,id. OR (Severely adj2 mentally impaired).ti,ab,id.

OR (Multiply adj2 **neurodevelopmentally impaired**).ti,ab,id. OR (Profoundly adj2 neurodevelopmentally impaired).ti,ab,id. OR (Seriously adj2 neurodevelopmentally impaired).ti,ab,id. OR (Severely adj2 neurodevelopmentally impaired).ti,ab,id. OR (Trainable adj2 mentally impaired).ti,ab,id. OR (Complex adj2 developmental impairment*).ti,ab,id.

OR (Multiple adj2 **developmental impairment***).ti,ab,id. OR (Profound adj2 developmental impairment*).ti,ab,id. OR (Serious adj2 developmental impairment*).ti,ab,id. OR (Severe adj2 developmental impairment*).ti,ab,id.

OR (Complex adj2 **intellectual impairment***).ti,ab,id. OR (Multiple adj2 intellectual impairment*).ti,ab,id. OR (Profound adj2 intellectual impairment*).ti,ab,id. OR (Serious adj2 intellectual impairment*).ti,ab,id. OR (Severe adj2 intellectual impairment*).ti,ab,id.

OR (Complex adj2 **mental impairment***).ti,ab,id. OR (Multiple adj2 mental impairment*).ti,ab,id. OR (Profound adj2 mental impairment*).ti,ab,id. OR (Serious adj2 mental impairment*).ti,ab,id. OR (Severe adj2 mental impairment*).ti,ab,id.

OR (Complex adj2 **neurodevelopmental impairment***).ti,ab,id. OR (Multiple adj2 neurodevelopmental impairment*).ti,ab,id. OR (Profound adj2 neurodevelopmental impairment*).ti,ab,id. OR (Serious adj2 neurodevelopmental impairment*).ti,ab,id. OR (Severe adj2 neurodevelopmental impairment*).ti,ab,id. OR (Trainable adj2 mental impairment*).ti,ab,id.

OR (Complex adj2 **cognitive incapacit***).ti,ab,id. OR (Multiple adj2 cognitive incapacit*).ti,ab,id. OR (Profound adj2 cognitive incapacit*).ti,ab,id. OR (Serious adj2 cognitive incapacit*).ti,ab,id. OR (Severe adj2 cognitive incapacit*).ti,ab,id.

OR (Complex adj2 **developmental incapacit***).ti,ab,id. OR (Multiple adj2 developmental incapacit*).ti,ab,id. OR (Profound adj2 developmental incapacit*).ti,ab,id. OR (Serious adj2 developmental incapacit*).ti,ab,id. OR (Severe adj2 developmental incapacit*).ti,ab,id.

OR (**Complex adj2 intellectual incapacit***).ti,ab,id. OR (Multiple adj2 intellectual incapacit*).ti,ab,id. OR (Profound adj2 intellectual incapacit*).ti,ab,id. OR (Serious adj2 intellectual incapacit*).ti,ab,id. OR (Severe adj2 intellectual incapacit*).ti,ab,id.

OR (Complex adj2 **mental incapacit***).ti,ab,id. OR (Multiple adj2 mental incapacit*).ti,ab,id. OR (Profound adj2 mental incapacit*).ti,ab,id. OR (Serious adj2 mental incapacit*).ti,ab,id. OR (Severe adj2 mental incapacit*).ti,ab,id.

OR (Complex adj2 **neurodevelopmental incapacit***).ti,ab,id. OR (Multiple adj2 neurodevelopmental incapacit*).ti,ab,id. OR (Profound adj2 neurodevelopmental incapacit*).ti,ab,id. OR (Serious adj2 neurodevelopmental incapacit*).ti,ab,id. OR (Severe adj2 neurodevelopmental incapacit*).ti,ab,id.

OR (Multiply adj2 **cognitively incapacitated**).ti,ab,id. OR (Profoundly adj2 cognitively incapacitated).ti,ab,id. OR (Seriously adj2 cognitively incapacitated).ti,ab,id. OR (Severely adj2 cognitively incapacitated).ti,ab,id.

OR (Multiply adj2 **developmentally incapacitated**).ti,ab,id. OR (Profoundly adj2 developmentally incapacitated).ti,ab,id. OR (Seriously adj2 developmentally incapacitated).ti,ab,id. OR (Severely adj2 developmentally incapacitated).ti,ab,id.

OR (Multiply adj2 **intellectually incapacitated**).ti,ab,id. OR (Profoundly adj2 intellectually incapacitated).ti,ab,id. OR (Seriously adj2 intellectually incapacitated).ti,ab,id. OR (Severely adj2 intellectually incapacitated).ti,ab,id.

OR (Multiply adj2 **mentally incapacitated**).ti,ab,id. OR (Profoundly adj2 mentally incapacitated).ti,ab,id. OR (Seriously adj2 mentally incapacitated).ti,ab,id. OR (Severely adj2 mentally incapacitated).ti,ab,id.

OR (Multiply adj2 **neurodevelopmentally incapacitated**).ti,ab,id. OR (Profoundly adj2 neurodevelopmentally incapacitated).ti,ab,id. OR (Seriously adj2 neurodevelopmentally incapacitated).ti,ab,id. OR (Severely adj2 neurodevelopmentally incapacitated).ti,ab,id.

OR (Polyhandicap).ti,ab,id. OR (Polyhandicaps).ti,ab,id. OR (Trainable adj2 mentally retardate*).ti,ab,id.

OR (Complex adj2 **cognitive retardation**).ti,ab,id. OR (Multiple adj2 cognitive retardation).ti,ab,id. OR (Profound adj2 cognitive retardation).ti,ab,id. OR (Serious adj2 cognitive retardation).ti,ab,id. OR (Severe adj2 cognitive retardation).ti,ab,id.

OR (Complex adj2 **developmental retardation**).ti,ab,id. OR (Multiple adj2 developmental retardation).ti,ab,id. OR (Profound adj2 developmental retardation).ti,ab,id. OR (Serious adj2 developmental retardation).ti,ab,id. OR (Severe adj2 developmental retardation).ti,ab,id.

OR (Complex adj2 **intellectual retardation**).ti,ab,id. OR (Multiple adj2 intellectual retardation).ti,ab,id. OR (Profound adj2 intellectual retardation).ti,ab,id. OR (Serious adj2 intellectual retardation).ti,ab,id. OR (Severe adj2 intellectual retardation).ti,ab,id.

OR (Complex adj2 **mental retardation**).ti,ab,id. OR (Multiple adj2 mental retardation).ti,ab,id. OR (Profound adj2 mental retardation).ti,ab,id. OR (Serious adj2 mental retardation).ti,ab,id. OR (Severe adj2 mental retardation).ti,ab,id. OR (Trainable adj2 mental retardation).ti,ab,id.

OR (Complex adj2 **neurodevelopmental retardation**).ti,ab,id. OR (Multiple adj2 neurodevelopmental retardation).ti,ab,id. OR (Profound adj2 neurodevelopmental retardation).ti,ab,id. OR (Serious adj2 neurodevelopmental retardation).ti,ab,id. OR (Severe adj2 neurodevelopmental retardation).ti,ab,id.

OR (Multiply adj2 **cognitively retarded**).ti,ab,id. OR (Profoundly adj2 cognitively retarded).ti,ab,id. OR (Seriously adj2 cognitively retarded).ti,ab,id. OR (Severely adj2 cognitively retarded).ti,ab,id.

OR (Multiply adj2 **developmentally retarded**).ti,ab,id. OR (Profoundly adj2 developmentally retarded).ti,ab,id. OR (Seriously adj2 developmentally retarded).ti,ab,id. OR (Severely adj2 developmentally retarded).ti,ab,id.

OR (**Multiply adj2 intellectually retarded**).ti,ab,id. OR (Profoundly adj2 intellectually retarded).ti,ab,id. OR (Seriously adj2 intellectually retarded).ti,ab,id. OR (Severely adj2 intellectually retarded).ti,ab,id.

OR (Multiply adj2 **mentally retarded**).ti,ab,id. OR (Profoundly adj2 mentally retarded).ti,ab,id. OR (Seriously adj2 mentally retarded).ti,ab,id. OR (Severely adj2 mentally retarded).ti,ab,id.

OR (Multiply adj2 **neurodevelopmentally retarded**).ti,ab,id. OR (Profoundly adj2 neurodevelopmentally retarded).ti,ab,id. OR (Seriously adj2 neurodevelopmentally retarded).ti,ab,id. OR (Severely adj2 neurodevelopmentally retarded).ti,ab,id. OR (Trainable adj2 mentally retarded).ti,ab,id. OR (Trainable adj2 retarded).ti,ab,id.)

AND

((patient adj2 acuity).ti,ab,id. OR (Severity adj2 illness).ti,ab,id. OR (illness adj2 severit*).ti,ab,id. OR (disease adj2 severit*).ti,ab,id. OR (sickness).ti,ab,id. OR exp Patient Reported Outcome Measures/ OR (patient adj1 reported adj1 outcome*).ti,ab,id.

OR exp Pain/ OR pain*.ti,ab,id. OR exp Comorbidity/ OR comorbidit*.ti,ab,id. OR (medical adj2 condition*).ti,ab,id. OR (clinical adj2 characteristic*).ti,ab,id. OR (health adj2 status).ti,ab,id. OR (physical adj2 condition*).ti,ab,id. OR (chronic adj2 disease*).ti,ab,id. OR (health adj2 problem*).ti,ab,id. OR (health adj2 indicator*).ti,ab,id. OR (health adj2 issue*).ti,ab,id. OR (secondary adj2 condition*).ti,ab,id. OR (physical adj2 health*).ti,ab,id.

OR exp Nutritional deficiencies/ OR (nutritional adj2 deficienc*).ti,ab,id. OR (enteral adj2 nutrition).ti,ab,id. OR (tube feed*).ti,ab,id. OR (tube fed*).ti,ab,id. OR (enteral feeding).ti,ab,id. OR gastrostom*.ti,ab,id.

OR exp musculoskeletal disorders/ OR (orthopedic adj2 disorder*).ti,ab,id. OR (orthopaedic adj2 disorder*).ti,ab,id. OR (scoliosis).ti,ab,id.

OR exp Epilepsy/ OR (epilepsy).ti,ab,id. OR (seizures).ti,ab,id. OR exp osteoporosis/ OR osteoporos*.ti,ab,id. OR exp Sleep wake Disorder/ OR (sleep adj2 disorder*).ti,ab,id. OR (sleep adj2 problem*).ti,ab,id. OR exp Vision Disorders/ OR (visual adj2 impairment).ti,ab,id. OR exp Hearing Disorders/ OR (hearing adj2 impairment).ti,ab,id. OR (Hearing adj2 disorder*).ti,ab,id. OR exp Dental Health/ OR (dental adj2 health).ti,ab,id. OR (dental adj2 care).ti,ab,id. OR (dental adj2 problem*).ti,ab,id. OR exp Urinary Incontinence/ OR (urine adj2 incontinence).ti,ab,id. OR exp Constipation/ OR (constipation).ti,ab,id.

OR exp Respiratory Tract Disorders/ OR exp Infectious Disorders/ OR (respiratory adj2 infection*).ti,ab,id. OR exp Body Size/ OR underweight.ti,ab,id. OR overweight.ti,ab,id. OR decubitus.ti,ab,id. OR (pressure adj2 ulcer).ti,ab,id. OR exp Muscle Spasms/ OR (muscle adj2 spasticit*).ti,ab,id. OR (muscle adj2 spasm*).ti,ab,id.

OR exp Dysphagia/ OR (gastroesophageal adj2 reflux) .ti,ab,id. OR dysphagia.ti,ab,id. OR Sialorrhea.ti,ab,id. OR drooling.ti,ab,id. OR exp Hypertension/ OR hypertension.ti,ab,id.

OR exp Endocrine Disorders/ OR (endocrine adj2 disorder*).ti,ab,id. OR (metabolic syndrome).ti,ab,id. OR exp Menstrual Cycle/ OR (menstrual cycle).ti,ab,id. OR (congenital adj2 heart defect*).ti,ab,id.)

**Web of Science Search**

(“Complex developmental abnormalit*” OR “Multiple developmental abnormalit*” OR “Profound developmental abnormalit*” OR “Serious developmental abnormalit*” OR “Severe developmental abnormalit*” OR “Complex intellectual abnormalit*” OR “Multiple intellectual abnormalit*” OR “Profound intellectual abnormalit*” OR “Serious intellectual abnormalit*” OR “Severe intellectual abnormalit*” OR “Complex mental abnormalit*” OR “Multiple mental abnormalit*” OR “Profound mental abnormalit*” OR “Serious mental abnormalit*” OR “Severe mental abnormalit*” OR “Complex neurodevelopmental abnormalit*” OR “Multiple neurodevelopmental abnormalit*” OR “Profound neurodevelopmental abnormalit*” OR “Serious neurodevelopmental abnormalit*” OR “Severe neurodevelopmental abnormalit*” OR “Complex cognitive defect*” OR “Multiple cognitive defect*” OR “Profound cognitive defect*” OR “Serious cognitive defect*” OR “Severe cognitive defect*” OR “Complex developmental defect*” OR “Multiple developmental defect*” OR “Profound developmental defect*” OR “Serious developmental defect*” OR “Severe developmental defect*” OR “Complex intellectual defect*” OR “Multiple intellectual defect*” OR “Profound intellectual defect*” OR “Serious intellectual defect*” OR “Severe intellectual defect*” OR “Complex mental defect*” OR “Multiple mental defect*” OR “Profound mental defect*” OR “Serious mental defect*” OR “Severe mental defect*” OR “Complex neurodevelopmental defect*” OR “Multiple neurodevelopmental defect*” OR “Profound neurodevelopmental defect*” OR “Serious neurodevelopmental defect*” OR “Severe neurodevelopmental defect*” OR “Multiply cognitively defective” OR “Profoundly cognitively defective” OR “Seriously cognitively defective” OR “Severely cognitively defective” OR “Multiply developmentally defective” OR “Profoundly developmentally defective” OR “Seriously developmentally defective” OR “Severely developmentally defective” OR “Multiply intellectually defective” OR “Profoundly intellectually defective” OR “Seriously intellectually defective” OR “Severely intellectually defective” OR “Multiply mentally defective” OR “Profoundly mentally defective” OR “Seriously mentally defective” OR “Severely mentally defective” OR “Multiply neurodevelopmentally defective” OR “Profoundly neurodevelopmentally defective” OR “Seriously neurodevelopmentally defective” OR “Severely neurodevelopmentally defective” OR “Complex cognitive deficienc*” OR “Multiple cognitive deficienc*” OR “Profound cognitive deficienc*” OR “Serious cognitive deficienc*” OR “Severe cognitive deficienc*” OR “Complex developmental deficienc*” OR “Multiple developmental deficienc*” OR “Profound developmental deficienc*” OR “Serious developmental deficienc*” OR “Severe developmental deficienc*” OR “Complex intellectual deficienc*” OR “Multiple intellectual deficienc*” OR “Profound intellectual deficienc*” OR “Serious intellectual deficienc*” OR “Severe intellectual deficiencOR “Complex mental deficienc*” OR “Multiple mental deficienc*” OR “Profound mental deficienc*” OR “Serious mental deficienc*” OR “Severe mental deficienc*” OR “Complex neurodevelopmental deficienc*” OR “Multiple neurodevelopmental deficienc*” OR “Profound neurodevelopmental deficienc*” OR “Serious neurodevelopmental deficienc*” OR “Severe neurodevelopmental deficienc*” OR “Multiply cognitively deficient” OR “Profoundly cognitively deficient” OR “Seriously cognitively deficient” OR “Severely cognitively deficient” OR “Multiply developmentally deficient” OR “Profoundly developmentally deficient” OR “Seriously developmentally deficient” OR “Severely developmentally deficient” OR “Multiply intellectually deficient” OR “Profoundly intellectually deficient” OR “Seriously intellectually deficient” OR “Severely intellectually deficient” OR “Multiply mentally deficient” OR “Profoundly mentally deficient” OR “Seriously mentally deficient” OR “Severely mentally deficient” OR “Multiply neurodevelopmentally deficient” OR “Profoundly neurodevelopmentally deficient” OR “Seriously neurodevelopmentally deficient” OR “Severely neurodevelopmentally deficient” OR “Complex developmental deficit*” OR “Multiple developmental deficit*” OR “Profound developmental deficit*” OR “Serious developmental deficit*” OR “Severe developmental deficit*” OR “Complex intellectual deficit*” OR “Multiple intellectual deficit*” OR “Profound intellectual deficit*” OR “Serious intellectual deficit*” OR “Severe intellectual deficit*” OR “Complex mental deficit*” OR “Multiple mental deficit*” OR “Profound mental deficit*” OR “Serious mental deficit*” OR “Severe mental deficit*” OR “Complex neurodevelopmental deficit*” OR “Multiple neurodevelopmental deficit*” OR “Profound neurodevelopmental deficit*” OR “Serious neurodevelopmental deficit*” OR “Severe neurodevelopmental deficit*” OR “Complex cognitive delay” OR “Multiple cognitive delay” OR “Profound cognitive delay” OR “Serious cognitive delay” OR “Severe cognitive delay” OR “Complex developmental delay” OR “Multiple developmental delay” OR “Profound developmental delay” OR “Serious developmental delay” OR “Severe developmental delay” OR “Complex intellectual delay” OR “Multiple intellectual delay” OR “Profound intellectual delay” OR “Serious intellectual delay” OR “Severe intellectual delay” OR “Complex mental delay” OR “Multiple mental delay” OR “Profound mental delay” OR “Serious mental delay” OR “Severe mental delay” OR “Complex neurodevelopmental delay” OR “Multiple neurodevelopmental delay” OR “Profound neurodevelopmental delay” OR “Serious neurodevelopmental delay” OR “Severe neurodevelopmental delay” OR “Multiply cognitively delayed” OR “Profoundly cognitively delayed” OR “Seriously cognitively delayed” OR “Severely cognitively delayed” OR “Multiply developmentally delayed” OR “Profoundly developmentally delayed” OR “Seriously developmentally delayed” OR “Severely developmentally delayed” OR “Multiply intellectually delayed” OR “Profoundly intellectually delayed” OR “Seriously intellectually delayed” OR “Severely intellectually delayed” OR “Multiply mentally delayed” OR “Profoundly mentally delayed” OR “Seriously mentally delayed” OR “Severely mentally delayed” OR “Multiply neurodevelopmentally delayed” OR “Profoundly neurodevelopmentally delayed” OR “Seriously neurodevelopmentally delayed” OR “Severely neurodevelopmentally delayed” OR “Multiply differently-abled” OR “Profoundly differently-abled” OR “Seriously differently-abled” OR “Severely differently-abled” OR “Multiple developmental difficult*” OR “Complex developmental difficult*” OR “Profound developmental difficult*” OR “Serious developmental difficult*” OR “Severe developmental difficult*” OR “Multiple intellectual difficult*” OR “Complex intellectual difficult*” OR “Profound intellectual difficult*” OR “Serious intellectual difficult*” OR “Severe intellectual difficult*” OR “Multiple mental difficult*” OR “Complex mental difficult*” OR “Profound mental difficult*” OR “Serious mental difficult*” OR “Severe mental difficult*” OR “Complex neurodevelopmental difficult*” OR “Multiple neurodevelopmental difficult*” OR “Profound neurodevelopmental difficult*” OR “Serious neurodevelopmental difficult*” OR “Severe neurodevelopmental difficult*” OR “Complex cognitive disabilit*” OR “Multiple cognitive disabilit*” OR “Profound cognitive disabilit*” OR “Serious cognitive disabilit*” OR “Severe cognitive disabilit*” OR “Complex developmental disabilit*” OR “Multiple developmental disabilit*” OR “Multiple developmental disabilit*” OR “Serious developmental disabilit*” OR “Severe developmental disabilit*” OR “Trainable intellectual disabilit*” OR “Complex intellectual disabilit*” OR “Multiple intellectual disabilit*” OR “Profound intellectual disabilit*” OR “Serious intellectual disabilit*” OR “Severe intellectual disabilit*” OR “Severe profound intellectual motor disabilit*” OR “Complex mental disabilit*” OR “Multiple mental disabilit*” OR “Profound mental disabilit*” OR “Serious mental disabilit*” OR “Severe mental disabilit*” OR “Trainable mental disabilit*” OR “Complex neurodevelopmental disabilit*” OR “Multiple neurodevelopmental disabilit*” OR “Profound neurodevelopmental disabilit*” OR “Serious neurodevelopmental disabilit*” OR “Severe neurodevelopmental disabilit*” OR “Multiple disabilit*” OR “Multiply cognitively disabled” OR “Profoundly cognitively disabled” OR “Seriously cognitively disabled” OR “Severely cognitively disabled” OR “Multiply developmentally disabled” OR “Profoundly developmentally disabled” OR “Seriously developmentally disabled” OR “Severely developmentally disabled” OR “Trainable developmentally disabled” OR “Multiply intellectually disabled” OR “Profoundly intellectually disabled” OR “Seriously intellectually disabled” OR “Severely intellectually disabled” OR “Trainable intellectually disabled” OR “Multiply mentally disabled” OR “Profoundly mentally disabled” OR “Seriously mentally disabled” OR “Severely mentally disabled” OR “Multiply neurodevelopmentally disabled” OR “Profoundly neurodevelopmentally disabled” OR “Seriously neurodevelopmentally disabled” OR “Severely neurodevelopmentally disabled” OR “Multiply disabled” OR “Trainable mentally disabled” OR “Complex developmental disorder*” OR “Multiple developmental disorder*” OR “Profound developmental disorder*” OR “Serious developmental disorder*” OR “Severe developmental disorder*” OR “Complex intellectual disorder*” OR “Multiple intellectual disorder*” OR “Profound intellectual disorder*” OR “Serious intellectual disorder*” OR “Severe intellectual disorder*” OR “Complex neurodevelopmental disorder*” OR “Multiple neurodevelopmental disorder*” OR “Profound neurodevelopmental disorder*” OR “Serious neurodevelopmental disorder*” OR “Severe neurodevelopmental disorder*” OR “Complex cognitive handicap*” OR “Multiple cognitive handicap*” OR “Profound cognitive handicap*” OR “Serious cognitive handicap*” OR “Severe cognitive handicap*” OR “Complex developmental handicap*” OR “Multiple developmental handicap*” OR “Profound developmental handicap*” OR “Serious developmental handicap*” OR “Severe developmental handicap*” OR “Complex intellectual handicap*” OR “Multiple intellectual handicap*” OR “Profound intellectual handicap*” OR “Serious intellectual handicap*” OR “Severe intellectual handicap*” OR “Complex mental handicap*” OR “Multiple mental handicap*” OR “Profound mental handicap*” OR “Serious mental handicap*” OR “Severe mental handicap*” OR “Trainable mental handicap*” OR “Complex neurodevelopmental handicap*” OR “Multiple neurodevelopmental handicap*” OR “Profound neurodevelopmental handicap*” OR “Serious neurodevelopmental handicap*” OR “Severe neurodevelopmental handicap*” OR “Multiply cognitively handicapped” OR “Profoundly cognitively handicapped” OR “Seriously cognitively handicapped” OR “Severely cognitively handicapped” OR “Multiply developmentally handicapped” OR “Profoundly developmentally handicapped” OR “Seriously developmentally handicapped” OR “Severely developmentally handicapped” OR “Multiply intellectually handicapped” OR “Multiply intellectually handicapped” OR “Profoundly intellectually handicapped” OR “Profoundly intellectually handicapped” OR “Seriously intellectually handicapped” OR “Seriously intellectually handicapped” OR “Severely intellectually handicapped” OR “Severely intellectually handicapped” OR “Multiply mentally handicapped” OR “Profoundly mentally handicapped” OR “Seriously mentally handicapped” OR “Severely mentally handicapped” OR “Multiply neurodevelopmentally handicapped” OR “Profoundly neurodevelopmentally handicapped” OR “Seriously neurodevelopmentally handicapped” OR “Severely neurodevelopmentally handicapped” OR “Multiply handicapped” OR “Trainable mentally handicapped” OR “Multiply developmentally impaired” OR “Profoundly developmentally impaired” OR “Seriously developmentally impaired” OR “Severely developmentally impaired” OR “Multiply intellectually impaired” OR “Profoundly intellectually impaired” OR “Seriously intellectually impaired” OR “Severely intellectually impairedOR “Multiply mentally impaired” OR “Profoundly mentally impaired” OR “Seriously mentally impaired” OR “Severely mentally impaired” OR “Multiply neurodevelopmentally impaired” OR “Profoundly neurodevelopmentally impaired” OR “Seriously neurodevelopmentally impaired” OR “Severely neurodevelopmentally impaired” OR “Trainable mentally impaired” OR “Complex developmental impairment*” OR “Multiple developmental impairment*” OR “Profound developmental impairment*” OR “Serious developmental impairment*” OR “Severe developmental impairment*” OR “Complex intellectual impairment*” OR “Multiple intellectual impairment*” OR “Profound intellectual impairment*” OR “Serious intellectual impairment*” OR “Severe intellectual impairment*” OR “Complex mental impairment*” OR “Multiple mental impairment*” OR “Profound mental impairment*” OR “Serious mental impairment*” OR “Severe mental impairment*” OR “Complex neurodevelopmental impairment*” OR “Multiple neurodevelopmental impairment*” OR “Profound neurodevelopmental impairment*” OR “Serious neurodevelopmental impairment*” OR “Severe neurodevelopmental impairment*” OR “Trainable mental impairment*” OR “Complex cognitive incapacit*” OR “Multiple cognitive incapacit*” OR “Profound cognitive incapacit*” OR “Serious cognitive incapacit*” OR “Severe cognitive incapacit*” OR “Complex developmental incapacit*” OR “Multiple developmental incapacit*” OR “Profound developmental incapacit*” OR “Serious developmental incapacit*” OR “Severe developmental incapacit*” OR “Complex intellectual incapacit*” OR “Multiple intellectual incapacit*” OR “Profound intellectual incapacit*” OR “Serious intellectual incapacit*” OR “Severe intellectual incapacit*” OR “Complex mental incapacit*” OR “Multiple mental incapacit*” OR “Profound mental incapacit*” OR “Serious mental incapacit*” OR “Severe mental incapacit*” OR “Complex neurodevelopmental incapacit*” OR “Multiple neurodevelopmental incapacit*” OR “Profound neurodevelopmental incapacit*” OR “Serious neurodevelopmental incapacit*” OR “Severe neurodevelopmental incapacit*” OR “Multiply cognitively incapacitated” OR “Profoundly cognitively incapacitated” OR “Seriously cognitively incapacitated” OR “Severely cognitively incapacitated” OR “Multiply developmentally incapacitated” OR “Profoundly developmentally incapacitated” OR “Seriously developmentally incapacitated” OR “Severely developmentally incapacitated” OR “Multiply intellectually incapacitated” OR “Profoundly intellectually incapacitated” OR “Seriously intellectually incapacitated” OR “Severely intellectually incapacitated” OR “Multiply mentally incapacitated” OR “Profoundly mentally incapacitated” OR “Seriously mentally incapacitated” OR “Severely mentally incapacitated” OR “Multiply neurodevelopmentally incapacitated” OR “Profoundly neurodevelopmentally incapacitated” OR “Seriously neurodevelopmentally incapacitated” OR “Severely neurodevelopmentally incapacitated” OR “Polyhandicap*” OR “Trainable mentally retardate*” OR “Complex cognitive retardation” OR “Multiple cognitive retardation” OR “Profound cognitive retardation” OR “Serious cognitive retardation” OR “Severe cognitive retardation” OR “Complex developmental retardation” OR “Multiple developmental retardation” OR “Profound developmental retardation” OR “Serious developmental retardation” OR “Severe developmental retardation” OR “Complex intellectual retardation” OR “Multiple intellectual retardation” OR “Profound intellectual retardation” OR “Serious intellectual retardation” OR “Severe intellectual retardation” OR “Complex mental retardation” OR “Multiple mental retardation” OR “Profound mental retardation” OR “Serious mental retardation” OR “Severe mental retardation” OR “Trainable mental retardation” OR “Complex neurodevelopmental retardation” OR “Multiple neurodevelopmental retardation” OR “Profound neurodevelopmental retardation” OR “Serious neurodevelopmental retardation” OR “Severe neurodevelopmental retardation” OR “Multiply cognitively retarded” OR “Profoundly cognitively retarded” OR “Seriously cognitively retarded” OR “Severely cognitively retarded” OR “Multiply developmentally retarded” OR “Profoundly developmentally retarded” OR “Seriously developmentally retarded” OR “Severely developmentally retarded” OR “Multiply intellectually retarded” OR “Profoundly intellectually retarded” OR “Seriously intellectually retarded” OR “Severely intellectually retarded” OR “Multiply mentally retarded” OR “Profoundly mentally retarded” OR “Seriously mentally retarded” OR “Severely mentally retarded” OR “Multiply neurodevelopmentally retarded” OR “Profoundly neurodevelopmentally retarded” OR “Seriously neurodevelopmentally retarded” OR “Severely neurodevelopmentally retarded” OR “Trainable mentally retarded” OR “Trainable retarded”)

(“patient acuity” OR “Severity of illness” “illness severit*” or “disease severit*”OR “sickness” OR “patient reported outcome*”

OR “pain*” OR “comorbidit*” OR “medical condition*” OR “clinical characteristic*” OR “health status” OR “physical condition*” OR “chronic disease*” OR "health problem*" OR "health indicator*" OR "health issue*" OR "secondary condition*" OR "physical health*" OR

“enteral nutrition” OR “nutritional deficienc*” OR “tube feed*” OR “tube fed*” OR “enteral feeding” OR “gastrostom*”

OR “musculoskeletal diseases” OR “orthopedic disorder*” OR “orthopaedic disorder*” OR “scoliosis”

OR “Epilepsy” OR “seizures” OR “osteoporos*” OR “dyssomnias” OR “sleep problem*” OR “sleep disorder*” OR "Vision Disorders" OR “visual impairment” OR "Hearing Loss" OR “hearing impairment” OR “hearing disorder*” OR “Dental Care” OR “dental health” OR “dental problem*” OR "Urinary Incontinence" OR “urine incontinence” OR "Constipation"

OR “Respiratory infection*” OR “respiratory tract infection*” OR “Body Size” OR underweight OR overweight OR decubitus OR “pressure ulcer” OR “muscle spasticit*” OR “muscle spasm*”

OR “gastroesophageal reflux” OR dysphagia OR Sialorrhea OR drooling OR hypertension

OR “Endocrine System Diseases” OR “endocrine disorder*” OR “Endocrine disease*” OR “metabolic syndrome” OR “menstrual cycle” OR “congenital heart defect*”)
